# Tuaimenals B–H, Merosesquiterpenes from the Irish Deep-Sea Soft Coral *Duva florida* with Bioactivity against Cervical Cancer Cell Lines

**DOI:** 10.1021/acs.jnatprod.2c00898

**Published:** 2022-12-29

**Authors:** Joshua T. Welsch, Tracess B. Smalley, Jenet K. Matlack, Nicole E. Avalon, Jennifer M. Binning, Mark P. Johnson, A. Louise Allcock, Bill J. Baker

**Affiliations:** † Department of Chemistry, University of South Florida, 4202 E. Fowler Avenue, CHE205, Tampa, Florida 33620, United States; ‡ Department of Molecular Oncology, H. Lee Moffitt Cancer Center and Research Institute, Tampa, Florida 33612, United States; § School of Natural Sciences and Ryan Institute, University of Galway, University Road, Galway, H91 TK33, Ireland

## Abstract

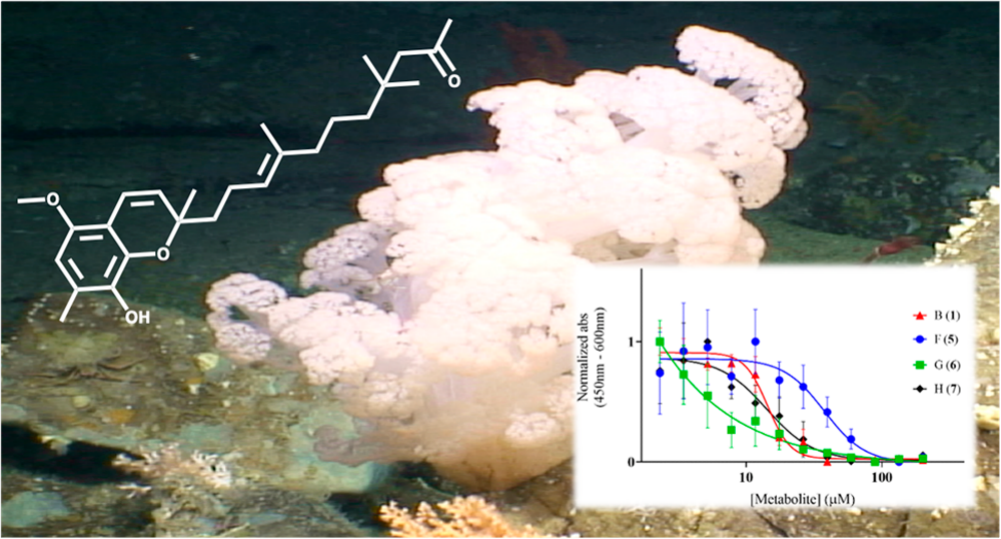

Previous chemical investigation of the Irish deep-sea soft coral *Duva florida* led to the identification of tuaimenal A (**10**), a new merosesquiterpene containing a highly substituted chromene core and modest cytotoxicity against cervical cancer. Further MS/MS and NMR-guided investigation of this octocoral has resulted in the isolation and characterization of seven additional tuaimenal analogs, B–H (**1**–**7**), as well as two known A-ring aromatized steroids (**8**, **9**), and additional tuaimenal A (**10**). Tuaimenals B, F, and G (**1**, **5**, **6**), bearing an oxygen at the C_5_ position, as well as monocyclic tuaimenal H (**7**), show increased cervical cancer inhibition profiles in comparison to that of **10**. Tuaimenal G further displayed potent, selective cytotoxicity with an EC_50_ value of 0.04 μM against the C33A cell line compared to the CaSki cell line (EC_50_ 20 μM). These data reveal the anticancer properties of tuaimenal analogs and suggest unique antiproliferation mechanisms across these secondary metabolites.

Analysis of all the newly approved drugs spanning the past four decades has shown that roughly 60% have been inspired by nature’s secondary metabolites, with that number soaring over 80% for small-molecule anticancer drugs.^1^ These metabolites are chemically diverse and innately specific compounds often produced as chemical defenses against neighboring organisms to increase the survivability of a species. The ocean’s deep seas may well represent the richest remaining source of new bioactive natural products capable of inspiring the next generation of cytotoxic therapeutic agents against diseases such as cervical cancer. With three-quarters of the earth’s surface covered in oceans, of which 95% exists below 1000 m, and advances such as remotely operated vehicles (ROVs) allowing for the targeted collections of deep-sea organisms only appearing in recent decades, the biotherapeutic potential of soft-bodied corals uniquely adapted to life in extreme conditions is only beginning to be explored.^2^ As the mysteries of the deep continue to be probed, the perception of a barren, lifeless wasteland existing within ocean trenches has given way to the realization that biological, and potentially chemical, diversity exists comparable to that found in tropical rain forests.^3^ While only around 2% of all natural products emanate from the deep seas, analysis of these secondary metabolites has revealed that nearly 75% possess bioactivity, further enhancing the urgency to explore the deep.^4^

Human papillomavirus (HPV) is a ubiquitous virus of the Papovaviridae family linked to 99.7% of cervical squamous cell cancer cases worldwide.^5^ Simultaneous binding of the HPV oncoproteins E6 and E7 to the p53 tumor suppressor protein and the pRB retinoblastoma tumor suppressor protein, respectively, allows for both the inhibition of typical apoptosis and a heightened rate of cell proliferation, ultimately cascading into a deadly cancer if left untreated.^6^ While a tremendous effort has been undertaken to provide HPV vaccinations as a primary prevention and comprehensive screenings as secondary prevention, cervical cancer remains the fourth most common cancer among women worldwide, with approximately 570 000 new cases and 311 000 deaths in 2018 across both HPV-positive and HPV-negative incidences.^7,8^ These rates are exceptionally higher in low- to middle-income, resource-poor nations where education, vaccinations, and screenings are less accessible, accounting for roughly 85% of all cervical cancer deaths.^6^

Previous chemical investigation into a collection of four specimens of the deep-sea Irish soft coral *Duva florida* afforded tuaimenal A (**10**). A merosesquiterpene with a highly substituted chromene core, **10** bears structural similarities to numerous terrestrial and marine secondary metabolites containing a “tocopherol-esque” backbone, perhaps most notably comparable to the two formylated tocotrienols 5-formyl-δ-tocotrienol and 7-formyl-δ-tocotrienol isolated from the stem bark of *Garcinia virgata*, with all three compounds possessing a benzaldehyde moiety.^9^ Justification for this unique substitution pattern about the chromene core was posed by Avalon et al. as resulting from a divergence from the typical tocopherol biosynthetic pathway at the homogentisate phytyltransferase step.^10^ Further, tuaimenal A showed promising potential in inhibiting the main protease of SARS-CoV-2 *in silico* as well as weak activity as a cytotoxic agent against cervical cancer.^10^ Herein we report further tandem mass spectrometry (MS/MS) and nuclear magnetic resonance (NMR) guided investigation into analogous metabolites resulting in the isolation, characterization, and cytotoxic evaluation of seven additional tuaimenals, B–H (**1**–**7**), two known A-ring-aromatized steroids (**8**, **9**), and tuaimenal A (**10**).
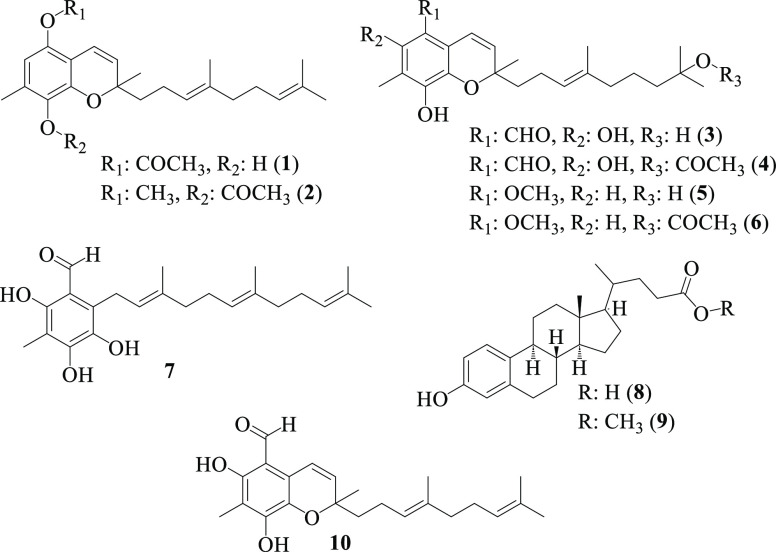


Tuaimenal B (**1**) was isolated as a pale orange oil. A molecular formula of C_24_H_32_O_4_ was established by HRESIMS ([M – H]^−^: *m*/*z* 383.2241, calcd 383.2228), corroborated by signals in the ^1^H and ^13^C NMR spectra (Table 1). Key ^1^H NMR signals (Figure 1) included a broad olefinic singlet H-4 (δ_H_ 6.32) demonstrating coupling in the COSY spectrum through C-3 (δ_C_ 118.0) to the benzylic methyl protons of H_3_-22 (δ_H_ 2.04), as well as HMBC correlations to three fully substituted carbons, C-2 (δ_C_ 147.1), C-3, and C-5 (δ_C_ 138.6), and additional four-bond correlations to C-1 (δ_C_ 137.8) and the olefinic carbon C-7 (δ_C_ 122.1). The singlet methyl protons H_3_-22 also displayed HMBC correlations to C-2 and C-3. The vinylic protons H-7 and H-8 (δ_H_ 6.24 and 5.57, respectively) were both observed as doublets with *J*-values of 9.8 Hz showing a COSY correlation to each other. The positions of H-7 and H-8 were assigned using HMBC correlations of H-7 to carbons C-5 and C-6 (δ_C_ 119.7), as well as the quaternary carbon C-9 (δ_C_ 78.6), in conjunction with HMBC correlations of H-8 to C-6, C-9, methylene carbon C-10 (δ_C_ 41.0), and methyl C-21 (δ_C_ 25.8). The singlet methyl protons on H_3_-21 (δ_H_ 1.33) also displayed HMBC correlations for C-9 and C-10. A triplet methylene H_2_-10 (δ_H_ 1.67) correlated in the HMBC spectrum to C-9, the adjacent methylene C-11 (δ_C_ 22.4) bearing two nonequivalent protons H_2_-11 (δ_H_ 2.07 and 2.13), as well the olefinic C-12 (δ_C_ 124.0) bearing triplet H-12 (δ_H_ 5.13). H_2_-11 additionally displayed correlations to C-12 as well as C-13 (δ_C_ 135.3). The olefinic proton H-12 displayed both COSY and HMBC correlations to the methyl protons H_3_-20 (δ_H_ 1.59) and carbon C-20 (δ_C_ 15.9) and an additional HMBC correlation to the methylene carbon C-14 (δ_C_ 39.7). The methylene triplet H_2_-14 (δ_H_ 1.97) correlated in the HMBC spectrum to both C-13 and methylene C-15 (δ_C_ 26.7), the latter carbon bearing a multiplet H_2_-15 (δ_H_ 2.06). H-16 (δ_H_ 5.09) also appeared as a triplet partially overlapping with the other side chain olefinic proton H-12; however H_2_-16 was placed on the basis of HMBC correlations to the two terminal methyl groups C-18 and C-19 (δ_C_ 17.7 and 25.7, respectively). Additionally, H_2_-16 displayed COSY correlations in both directions of the side chain to H_2_-15 as well as through C-17 (δ_C_ 131.3) to H_3_-18 (δ_H_ 1.60) and H_3_-19 (δ_H_ 1.68). H_3_-18 and H_3_-19 both displayed HMBC correlations to each other, as well as to C-17. H_3_-24 (δ_H_ 2.34) appeared as a methyl singlet with a chemical shift indicative of an acetyl group further supported by a single HMBC correlation to an ester-type carbonyl C-23 (δ_C_ 168.9). The C-12 and C-13 olefinic configuration was determined to be *E* by a NOESY correlation between H-12 and H_2_-14 linking these two protons in close proximity through space (Figure 1).

**Figure 1:**
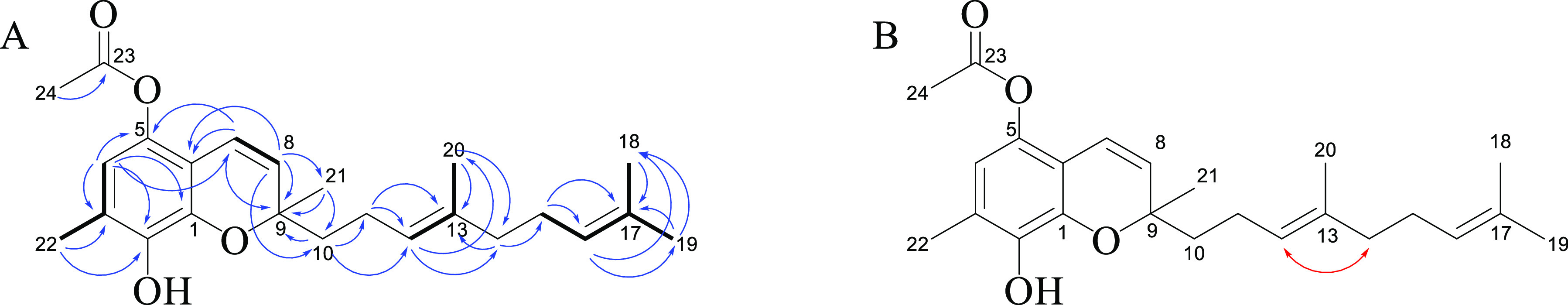
Key HMBC (→) and COSY (**―**) correlations (A) and key NOESY (↔) correlations (B) establishing the olefin configuration of tuaimenal B (**1**).

**Table 1:** NMR Data for Tuaimenal B (**1**)^a^

pos	δ_C_,^b^ type	δ_H_,^c^ (*J* in Hz)	HMBC^d^	COSY^d^	NOESY^d^
1	137.8, C				
2	147.1, C				
3	118.0, C				
4	109.8, CH	6.32, br s	1, 2, 3, 5, 7	22	22
5	138.6, C				
6	119.7, C				
7	122.1, CH	6.24, d (9.8)	4, 5, 6, 9	8	8
8	130.3, CH	5.57, d (9.8)	6, 9, 10, 21		21
9	78.6, C				
10	41.0, CH_2_	1.67, m	9, 11, 12, 21		
11	22.4, CH_2_	2.07, m	10, 12, 13		
		2.13, m	10, 12		
12	124.0, CH	5.13, t (6.54)	10, 11, 14, 20	20	14
13	135.3, C				
14	39.7, CH_2_	1.97, t (7.63)	12, 13, 15, 20		
15	26.7, CH_2_	2.06, m	13, 14, 16		
16	124.3, CH	5.09, t (7.08)	15, 18, 19	15, 18, 19	19
17	131.3, C				
18	17.7, CH_3_	1.60, s	16, 17, 19		
19	25.7, CH_3_	1.68, s	16, 17, 18		
20	15.9, CH_3_	1.59, s	12, 13, 14		
21	25.8, CH_3_	1.33, s	8, 9, 10		
22	9.3, CH_3_	2.04, s	2, 3, 4		
23	168.9, C				
24	20.4, CH_3_	2.34, s	23		

a CDCl_3_, ppm, type established by phase-sensitive HSQC.

b 150 MHz.

c 600 MHz.

d 500 MHz.

Tuaimenal C (**2**) was isolated as a pale yellow oil with spectral data similar to those of tuaimenal B (**1**). A molecular formula of C_25_H_34_O_4_ for **2** was established from HREIMS ([M^+^]: *m*/*z* 398.2452, calcd 398.2457), corroborated by ^1^H and ^13^C NMR spectra (Table 2). Tuaimenal C departed from the motif of **1** by the presence of a methoxy signal H_3_-23 (δ_H_ 3.84) in the ^1^H NMR spectrum, while retaining an acetyl group as indicated by H_3_-25 (δ_H_ 2.30). Assignment of the acetate on C-2 (δ_C_ 142.4) was determined through HMBC correlations of the singlet methyl H_3_-22 (δ_H_ 2.05) and a long-range correlation between the acetate protons H_3_-25 and the acetate bearing C-2. The assignment of the methoxy group on C-5 (δ_C_ 146.7) was confirmed by HMBC correlations between both H_3_-23 and the doublet olefinic signal H-7 (δ_C_ 6.27) to C-5. All remaining ^1^H and ^13^C NMR signals align with that of tuaimenal B including the aromatic proton H-4 (δ_H_ 6.45) on C-4 (δ_C_ 114.4) of the chromene core, as well as throughout the terpene side chain, and were confirmed through HMBC, COSY, and NOESY correlations.

**Table 2:** NMR Data for Tuaimenals C, D, E, F, G, and H (**2–7**)^a^

	tuaimenal C		tuaimenal D		tuaimenal E		tuaimenal F		tuaimenal G		tuaimenal H	
pos	*δ*_C_,^b^ type	*δ*_H_^c^ (*J* in Hz)	*δ*_C_,^b^ type	*δ*_H_^c^ (*J* in Hz)	*δ*_C_,^b^ type	*δ*_H_^c^ (*J* in Hz)	*δ*_C_,^b^ type	*δ*_H_^c^ (*J* in Hz)	*δ*_C_,^b^ type	*δ*_H_^c^ (*J* in Hz)	*δ*_C_,^b^ type	*δ*_H_^c^ (*J* in Hz)
1	143.7, C		138.6, C		132.2, C		139.5, C		147.3, C		134.2, C	
2	142.4, C		151.4, C		151.4, C		147.3, C		139.6, C		152.5, C	
3	124.5, C		111.8, C		111.8, C		118.2, C		118.1, C		109.6, C	
4	114.4, CH	6.45, s	158.2, C		158.2, C		107.5, CH	6.25, s	107.5, CH	6.26, s	159.5, C	
5	146.7, C		107.6, C		107.6, C		146.7, C		146.7, C		110.7, C	
6	119.9, C		118.6, C		118.7, C		119.8, C		119.8, C		126.9, C	
7	122.5, CH	6.27, d (9.8)	116.5, CH	6.87, d (10.2)	116.5, CH	6.86, d (10.2)	122.6, CH	6.24, ov d	122.6, CH	6.25, d, (9.8)	23.5, CH_2_	3.60, br s
8	129.6, CH	5.57, d (9.8)	132.4, CH	5.80, d (9.8)	132.4, CH	5.80, d (10.2)	129.7, CH	5.56, d (9.8)	129.7, CH	5.57, d (9.8)	121.1, CH	5.16, br t
9	79.0, C		79.2, C		79.1, C		78.1, C		78.1, C		139.2, C	
10	41.6, CH_2_	1.84, m	40.3, CH_2_	1.77, m	40.3, CH_2_	1.78, m	41.2, CH_2_	1.83, m	41.2, CH_2_	1.83, m	39.5, CH_2_	2.08, m
		1.67, m						1.67, m		1.68, m		
11	23.0, CH_2_	2.18, m	22.5, CH_2_	1.43, m	22.6, CH_2_	2.11, m	22.8, CH_2_	2.15, m	22.8, CH_2_	2.16, m	26.2, CH_2_	2.11, m
		2.12, m										
12	124.0, CH	5.13, t (6.47)	123.6, CH	5.12, t (7.08)	123.6, CH	5.11, t (6.99)	124.2, CH	5.14, t (7.08)	124.2, CH	5.13, t (6.9)	123.3, CH	5.04, t (6.36)
13	135.6, C		135.8, C		135.6, C		135.2, C		135.1, C		135.9, C	
14	39.8, CH_2_	1.97, t	39.9, CH_2_	1.97, t (6.18)	39.7, CH_2_	1.96, m	39.9, CH_2_	1.96, t (6.54)	39.7, CH_2_	1.95, t (7.63)	39.6, CH_2_	1.96, t (7.63)
15	26.8, CH_2_	2.06, m	22.6, CH_2_	2.13, m	22.0, CH_2_	1.68, m	22.6, CH_2_	1.44, m	22.1, CH_2_	1.40, m	26.6, CH_2_	2.03, m
						1.38, m						
16	124.4, CH	5.09, t (6.96)	43.4, CH_2_	1.43, m	40.2, CH_2_	1.69, m	43.3, CH_2_	1.42, m	40.4, CH_2_	1.67, m	124.2, CH	5.07, t (6.72)
17	131.5, C		70.1, C		82.4, C		71.1, C		82.4, C		131.4, C	
18	17.8, CH_3_	1.60, s	29.2, CH_3_	1.22, s	26.1, CH_3_	1.42, s	29.2, CH_3_	1.21, s	26.0, CH_3_	1.42, s	17.6, CH_3_	1.60, s
19	25.8, CH_3_	1.68, s	29.2, CH_3_	1.22, s	26.1, CH_3_	1.42, s	29.2, CH_3_	1.21, s	26.0, CH_3_	1.42, s	25.7, CH_3_	1.67, s
20	16.1, CH3	1.59, s	15.9, CH_3_	1.58, s	15.8, CH_3_	1.57, s	15.8, CH_3_	1.57, s	15.8, CH_3_	1.57, s	16.0, CH_3_	1.59, s
21	26.5, CH_3_	1.41, s	25.4, CH_3_	1.43, s	25.4, CH_3_	1.43, s	25.9, CH_3_	1.39, s	25.9, CH_3_	1.39, s	16.4, CH_3_	1.82, s
22	9.9, CH_3_	2.05, s	7.6, CH_3_	2.14, s	7.6, CH_3_	2.14, s	9.0, CH_3_	2.14, s	9.0, CH_3_	2.14, s	7.3, CH_3_	2.12, s
23	60.5, CH_3_	3.84, s	191.1, CH	10.09, s	191.0, CH	10.08, s	60.4, CH_3_	3.83, s	60.4, CH_3_	3.84, s	192.9, CH	9.98, s
24	169.8, C				170.5, C				170.6, C			
25	20.9, CH_3_	2.30, s			22.5, CH_3_	1.97, s			22.5, CH_3_	1.97, s		

a CDCl_3_, ppm.

b 150 MHz, type established by phase-sensitive HSQC.

c 600 MHz.

Tuaimenal D (**3**) was isolated as a pale orange oil with spectral data similar to that of tuaimenals B and C (**1**, **2**). A molecular formula of C_23_H_32_O_5_ for **3** was established from HRESIMS ([M – H]^−^: *m*/*z* 387.2197, calcd 387.2177), corroborated by ^1^H and ^13^C NMR spectra (Table 2). Tuaimenal D departed from the motif of **1** and **2** in four major ways observable in the ^1^H NMR spectrum: (1) the loss of the olefinic triplet at H-16, which now displayed as a methylene multiplet H_2_-16 (δ_H_ 1.43), resulting in the attached methyl groups H_3_-18 and H_3_-19 (δ_H_ 1.22) as newly equivalent, (2) a fifth oxygen noted in the chemical formula not accounted for in the ^1^H NMR spectrum when acquired in CDCl_3_, (3) a newly appearing downfield singlet H-23 (δ_H_ 10.09) indicative of the presence of an aldehyde, and (4) the loss of the aromatic proton signal H-4. The first two departures from the aforementioned motif were accounted for by the addition of a tertiary alcohol at the C-17 (δ_C_ 70.1) position on the basis of the chemical shift displaying congruence with that of a fully saturated, oxygen-bearing carbon. This placement also accounts for the loss of the C-16/C-17 olefin and would result in H_3_-18 and H_3_-19 appearing as overlapping equivalent signals in the ^1^H NMR spectrum, and it is not unexpected for this alcohol to be absent when acquiring NMR data with CDCl_3_. Additionally, HMBC correlations were observed from H_3_-18 to C-16 (δ_C_ 43.4), C-17, and C-19. The aldehyde moiety was determined to be at the aromatic C-5 (δ_C_ 107.6) position due to a single HMBC correlation for H-23 to C-5. The last departure from the motif of **1** and **2**, being the absence of an aromatic proton at the C-4 position, was justified by the placement of a phenol on C-4 (δ_C_ 158.2). This configuration was assigned on the basis of the downfield shift of this carbon indicating an oxygen-bearing aromatic position, HMBC correlations from the new alcohol proton to C-3 (δ_C_ 111.8), C-4, and C-5, and congruence with the chemical formula proposed from the HRESIMS data.

Tuaimenal E (**4**) was also isolated as a pale orange oil with spectral data similar to those of tuaimenal D (**3**). A molecular formula of C_25_H_34_O_6_ for **4** was established from HRESIMS ([M – H]^−^: *m*/*z* 429.2296, calcd 429.2283), corroborated by ^1^H and ^13^C NMR spectra (Table 2). Tuaimenal E departed from the motif of **3** by the presence of an acetyl methyl group H_3_-25 (δ_H_ 1.97) and additional deshielding of the two equivalent terminal side chain methyl groups H_3_-18 and H_3_-19 (δ_H_ 1.42). The additional two carbons, two protons, and one oxygen observed in **4** thus corresponded to an acetoxy group in place of the tertiary alcohol at the C-17 (δ_C_ 82.4), as observed for **3**. All ^1^H and ^13^C NMR chemical shifts, coupling, and integrations associated with the bicyclic chromene core for **4** aligned with that of **3**.

Tuaimenal F (**5**) was isolated as a yellow oil with spectral data similar to those of tuaimenal D (**3**). A molecular formula of C_23_H_34_O_4_ for **5** was established from HRESIMS ([M – H]^−^: *m*/*z* 373.2399, calcd 373.2384), corroborated by ^1^H and ^13^C NMR spectra (Table 2). Tuaimenal F departed from the motif of **3** by the loss of the deshielded phenol OH_a_ (δ_H_ 12.30) and aldehyde H-23 (δ_H_ 10.09), as well as the appearance of an aromatic singlet H-4 (δ_H_ 6.25) and methoxy singlet H_3_-23 (δ_H_ 3.83). The placement of H-4 and H_3_-23 was determined through HMBC correlations of the methyl singlet H_3_-22 (δ_H_ 2.14) to C-4 (δ_C_ 107.5) as well as both H_3_-23 and H-7 (δ_H_ 6.24) to C-5 (δ_C_ 146.7). The terminal tertiary alcohol of **3** was determined to be retained in **5** on the basis of ^1^H and ^13^C NMR shifts and HMBC correlations.

Tuaimenal G (**6**) was also isolated as a yellow oil with spectral data similar to those of tuaimenal F (**5**). A molecular formula of C_25_H_36_O_5_ for **6** was established from HRESIMS ([M + Na]^+^: *m*/*z* 439.2455, calcd 439.2455), corroborated by ^1^H and ^13^C NMR spectra (Table 2). Tuaimenal G departed from the motif of **5** by the presence of an acetyl methyl group H_3_-25 (δ_H_ 1.97) and additional deshielding of the two equivalent terminal side chain methyl groups H_3_-18 and H_3_-19 (δ_H_ 1.42). The additional two carbons, two protons, and one oxygen observed in **6** thus corresponded to an acetoxy group in place of the **5** tertiary alcohol at C-17 (δ_C_ 82.4). All ^1^H and ^13^C NMR chemical shifts, splitting patterns, and integrations associated with the bicyclic chromene core for **6** aligned with those of **5**.

Tuaimenal H (**7**) was isolated as a pale orange film with spectral data similar to those of tuaimenal D (**3**). A molecular formula of C_23_H_32_O_4_ for **7** was established from HRESIMS ([M – H]^−^: *m*/*z* 371.2245, calcd 371.2228), corroborated by ^1^H and ^13^C NMR spectra (Table 2). Tuaimenal H departed from the motif of **3** (Figure 2) by the loss of the olefinic doublets H-7 and H-8 as well as the presence of two additional olefinic triplets resulting in three side chain double bonds H-8 (δ_H_ 5.16), H-12 (δ_H_ 5.04), and H-16 (δ_H_ 5.07). Additionally, a broad deshielded singlet H_2_-7 (δ_H_ 3.60) integrating to 2, indicative of the presence of a methylene group allylic to two double bonds, was observed, resulting in the determination that the ether-containing ring in **7** was absent. HMBC correlations of H_3_-22 (δ_H_ 2.12) to the two phenol-bearing aromatic carbons C-2 (δ_C_ 152.5) and C-4 (δ_H_ 159.5), as well as from OH_a_ (δ_H_ 12.59) to C-3 (δ_C_ 109.6) and C-5 (δ_C_ 110.7), and the aldehyde singlet H-23 (δ_H_ 9.98) to C-4 and C-5 placed the substituents of the intact aromatic ring. H_2_-7 displayed COSY correlations through C-8 (δ_C_ 121.1) and C-9 (δ_C_ 139.2) to the singlet methyl H_3_-21 (δ_H_ 1.82) and triplet methylene H_2_-10 (δ_H_ 2.08), as well as HMBC correlations to the aromatic carbons C-1 (δ_C_ 134.2), C-5, and C-6 (δ_C_ 126.9) and olefinic C-8 and C-9. The olefinic singlet H-8 (δ_H_ 5.16) displayed HMBC correlations to the methyl C-21 (δ_C_ 16.4), as well as to the methylene C-10 (δ_C_ 39.5). H_2_-10 was shown to correlate in the HMBC to a methylene C-11 (δ_C_ 26.2), for which H-11 (δ_H_ 2.11) had a COSY correlation to the olefinic triplet H-12 (δ_H_ 5.04) and HMBC correlations to olefinic C-12 and C-13 (δ_C_ 135.9). The methyl C-20 (δ_C_ 16.0) was placed on C-13 due to correlations of H_3_-20 (δ_H_ 1.59) to C-13 and methylene C-14 (δ_C_ 39.6), for which the methylene triplet H_2_-14 (δ_H_ 1.96) displayed HMBC correlations to C-15 (δ_C_ 26.6) and C-16 (δ_C_ 124.2). The terminal end of the side chain was assigned on the basis of COSY correlations from H-16 (δ_H_ 5.07) to H_2_-15 (δ_H_ 2.03) and through C-17 (δ_C_ 131.4) to H_3_-18 and H_3_-19 (δ_H_ 1.60 and 1.67, respectively), as well as HMBC correlations from H_3_-18 to C-17 and C-19 (δ_C_ 25.7). The side chain double-bond configuration (Figure 2) was determined as 8*E*, 12*E* due to the observation of NOESY correlations of H_2_-7 to H_3_-21, H-8 to H_2_-10, H_2_-10 to H-12, and H-12 to H_2_-14.

**Figure 2:**
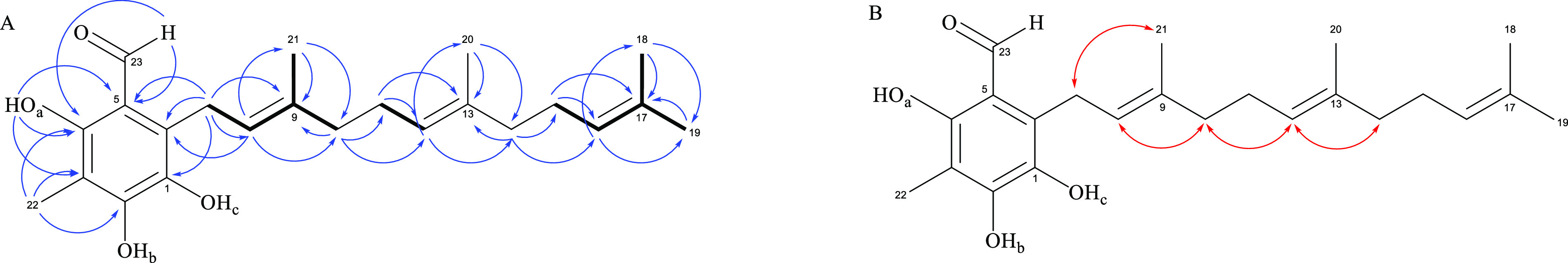
Key HMBC (→) and COSY (**―**) correlations (A) and key NOESY (↔) correlations (B) establishing the olefinic configurations of tuaimenal H (**7**).

Oxidized steroid **8** was isolated as a white, crystalline solid. A molecular formula of C_23_H_32_O_3_ was established from high-resolution electrospray ionization mass spectrometry (HRESIMS) ([M – H]^−^: *m*/*z* 355.2298, calcd 355.2279). Comparison of the HSQC using SMARTNMR, an online artificial intelligence-based database of natural products made available by the University of California San Diego, revealed a strong correlation (cos > 0.992) to that of 3-hydroxy-19-norchola-1,3,5^10^-trien-24-oic acid, a ring-A-aromatized bile acid originally reported from the marine sponge *Sollasella moretonensis*.^11,12^ Further comparison of the literature ^13^C NMR chemical shifts (Table S2) confirmed the structure of **8**. Absolute stereochemical determination on the gonane backbone of **8** was determined through X-ray crystallography (Figure S74).

Oxidized steroid **9** was isolated as a white, crystalline solid with spectral data similar to those of **8**. A molecular formula of C_24_H_34_O_3_ (**9**) was established from HRESIMS ([M + H]^+^: *m*/*z* 371.2589, calcd 375.2581), corroborated by the ^13^C NMR spectrum (Table S2). The presence of a methyl ester H_3_-25 (δ_H_ 3.69), correlating in the HMBC spectrum to the side chain carbonyl C-24 (δ_C_ 175.0), was the major departure of **9** from **8**.

Additional mass of tuaimenal A (**10**) was isolated from previously uninvestigated fractions of *D. florida* extract as a yellow oil matching the previously reported configuration of this metabolite on the basis of MS and NMR spectral data. A molecular formula of C_23_H_30_O_4_ for **10** was confirmed from HRESIMS ([M – H]^−^: *m*/*z* 369.2089, calcd 369.2071), corroborated by ^1^H and ^13^C NMR spectra. Interestingly, **10** was previously assigned by Avalon et al. (2022) as existing in the *R* configuration about the C-9 stereocenter on the basis of comparisons of experimental vibrational circular dichroism (VCD) data to computationally predicted values for each enantiomer.^10^ While significant overlap between experimental data and *in silico* predictions did exist indicating this assignment of absolute configuration, the observed VCD and optical rotation (OR) signals were both inconsistently low, incongruent with an enantiomerically pure metabolite. To determine if this trait was maintained for compounds **1**–**7**, optical rotation data were recorded at the maximal concentrations for measurement for each compound using a 50 μL cell, with the results displayed in Table 3.

**Table 3:** Measured Optical Rotations in CHCl_3_ of All Tuaimenals Bearing a Stereocenter at the C-9 Position

compound	concentration (g/mL)	optical rotation ([α]^25^_D_)
tuaimenal B (**1**)	0.058	0.0
tuaimenal C (**2**)	0.008	0.0
tuaimenal D (**3**)	0.018	1.1
tuaimenal E (**4**)	0.034	2.6
tuaimenal F (**5**)	0.059	1.5
tuaimenal G (**6**)	0.065	1.1
tuaimenal A (**10**)	0.065	0.5

The lack of significant optical rotation observed for all of the tuaimenals bearing the C-9 stereocenter indicated that these metabolites may in fact be present as racemic mixtures at the time of analysis, or at least with exceedingly low values for enantiomeric excess (ee). To confirm this finding, tuaimenal E (**4**), which showed, while still incredibly low, the highest value for optical rotation at [α]^25^_D_ 2.6, was selected to be analyzed with a chiral-phase HPLC column with integrations calculated for the area under the curve of the signal from each enantiomer.

The results of this analysis showed relative areas of 49.8% and 51.2% for the two enantiomers present in the sample, leading to the conclusion that the tuaimenals, including tuaimenal A (**10**), which was reisolated, exist with sufficiently low ee that any further attempts at deciphering the predominant configuration would yield inconclusive results, and thus should be considered racemic mixtures. Further support of this theory is drawn from the recent review of naturally occurring 6-hydroxy-chromanols and 6-hydroxy-chromenols, which cited 73% of marine-derived chromanols as having been isolated in the *R* configuration, while only 26% of chromenols displayed the same stereochemistry, indicating that the chromenols as described herein may originate via a cyclization reaction that is not biosynthetically enzymatically catalyzed.^13^

Compounds isolated with sufficient mass for biological evaluation were tested for antiproliferative effects *in vitro* against two cervical cancer cell lines (Figure 3) at metabolite concentrations ranging from 2 to 200 μM. While none of the tested compounds revealed <10 μM effects on the HPV-positive CaSki cell line, all new metabolites showed increased activity in comparison to tuaimenal A (**10**) for both cell lines. Tuaimenal G (**6**) displayed potent activity in the C33A cell line with an EC_50_ value of 0.04 μM (Table 4). In contrast to the other evaluated metabolites, tuaimenal G additionally showed high specificity toward the HPV-negative cell line with a 500-fold difference in EC_50_ values between the C33A and CaSki cell lines. The 2018 review by Birringer et al. of naturally occurring chromanols and chromenols analyzed the structural motifs of such meroterpenoids in relation to anticancer activity and drew several conclusions of importance to the current study: (1) the chromanol/chromenol core is indespensible for activity, (2) the degree of methylation about this core is inversely related to activity, and (3) side chain olefins at C-12 and C-16 have little influence on activity.^13^ To these points, we have found (1) the tuaimenal core is able to retain comparable levels of cytotoxicity against the cell line C33A despite decylcization of the dihydropyran ring, (2) there exists a great deal of variability in activity among metabolites with a singular methyl group on the aromatic ring, and (3) it is correct in assessing that the side chain olefins play little role in activity. Additionally, on the basis of a nearly 1000-fold increase in activity from tuaimenal G (**6**) to tuaimenal F (**5**), it may be concluded that functionalization at C-17 as well as C-4 plays a pivotal role in activity against this cancerous cell line. Positive controls using 50 μM etoposide and negative controls in the absence of metabolite were conducted simultaneously for both cell lines to ensure viability of the assays (Figure 3). This observed specificity should be further probed using a larger panel of cancerous cell lines. Additional testing revealed no reportable antifungal activity against seven strains of *Candida*, antibiotic activity against the ESKAPE pathogens and *Mycobacterium tuberculosis*, or antiviral activity against human respiratory syncytial virus, indicating selective cytotoxicity.

**Figure 3:**
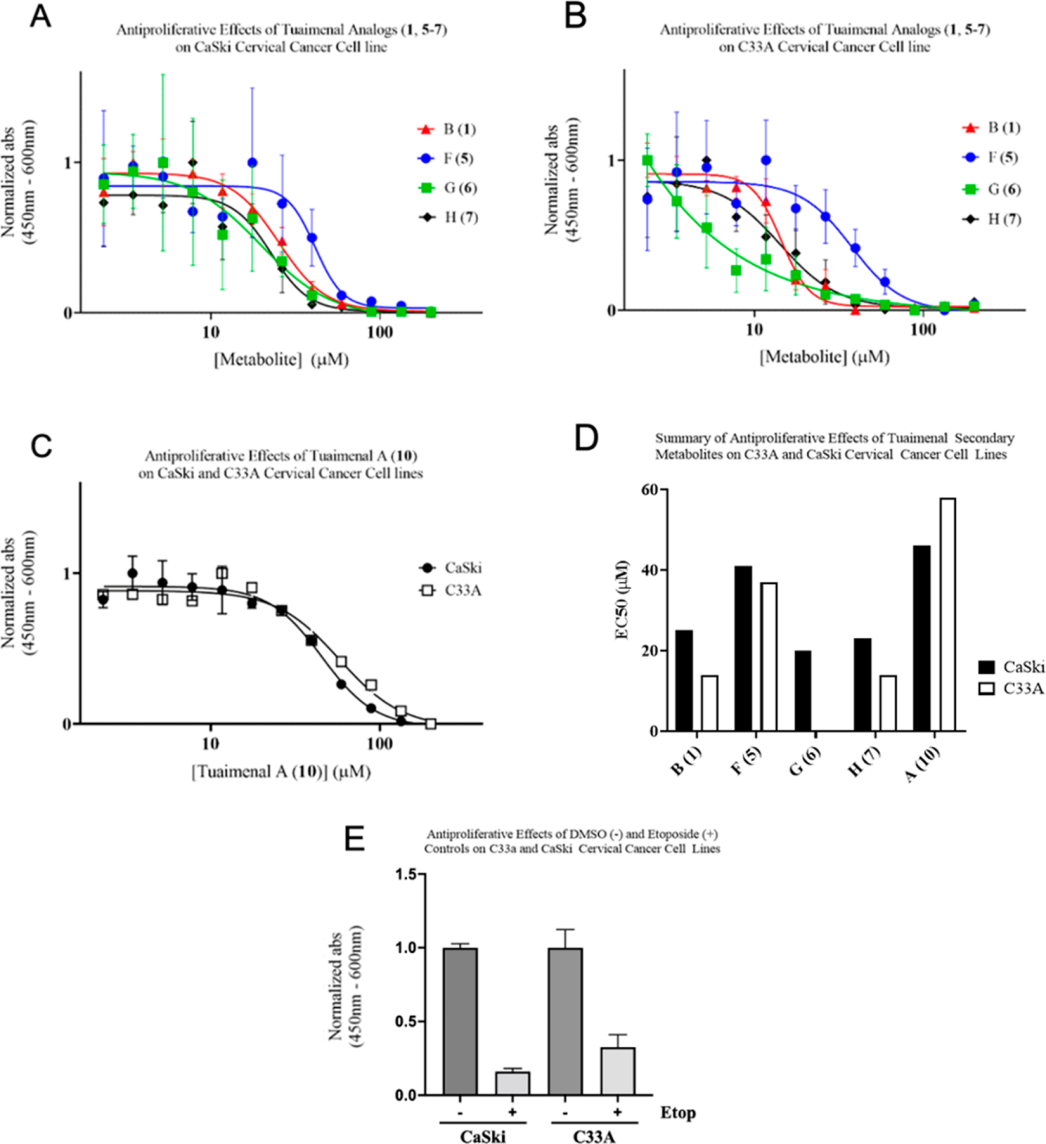
Antiproliferative effect of **1** and **5**–**7** on CaSki (A) and C33A cells (B) and **10** in CaSki and C33A cells (C), summary of antiproliferative effects of all tested tuaimenals (D), and DMSO (negative) and etoposide (positive) control data (E). All experiments were seeded at 1000–3000 cells per well in a 96-well plate and treated with varying concentrations of each metabolite (*N* = 3). At 48 h post-treatment proliferation was observed after a 2 h incubation with WST reagent (10 μL in 190 μL media/well). Nonlinear regression curves were fitted with GraphPad Prism software.

**Table 4:** Calculated EC_50_ Values for Antiproliferative Effect of Tuaimenals B, F, G, H, and A (**1**, **5**–**7**, **10**) against the Cervical Cancer CaSki and C33A Cell Lines

compound	CaSki EC_50_ (μM)	C33A EC_50_ (μM)
tuaimenal B (**1**)	25	14
tuaimenal F (**5**)	41	38
tuaimenal G (**6**)	20	0.04
tuaimenal H (**7**)	23	14
tuaimenal A (**10**)	46	58

This study represents the second chemical investigation into the secondary metabolome of the Irish deep-sea coral *D. florida* for which only tuaimenal A (**10**) was previously reported. We report the isolation, structural elucidation, and biological evaluation of seven new analogous tuaimenals, B–H (**1**–**7**), all of which display increased levels of antiproliferative effects against both HPV-positive and HPV-negative cervical cancer cell lines, with tuaimenal G possessing potent *in vitro* cytotoxicity and selectivity against HPV-negative cervical cancer cell lines.

## Experimental Section

### General Experimental Procedures

Solvents were obtained from Fisher Scientific Co. and were HPLC grade (>99% purity) unless otherwise stated. UV absorptions were measured with an Agilent Cary 60 UV–vis spectrophotometer with a 10 mm path length fiber optic probe in acetonitrile. IR spectra were recorded as a thin film with an Agilent Cary 630 FTIR. NMR spectra were acquired in CDCl_3_ with residual solvent referenced as the internal standard (δ_H_ 7.27; δ_C_ 77.0) for ^1^H and ^13^C NMR spectra, respectively. The ^1^H and ^13^C spectra were recorded on a Bruker 600 MHz broadband instrument with the ^13^C spectra recorded at 150 MHz. 2D NMR experiments were recorded on a Varian 500 MHz broadband instrument. GC/MS analysis was performed on an Agilent 7890A GC using a Zebron ZB-5HT Inferno (30 m × 0.25 mm, 0.25 μm film thickness) column coupled to an Agilent 7200 accurate-mass QToF with electron impact ionization. LC/MS analysis was performed on an Agilent 1260 Infinity LC coupled to an Agilent 6540 UHD accurate-mass QToF with electrospray ionization. MPLC fractionation and analysis was performed on a Teledyne-Isco CombiFlash Rf system equipped with built-in UV detection at 254 and 280 nm. HPLC fractionation and analysis were performed on a Shimadzu LC-20AR system equipped with a Shimadzu SPD-20A UV/vis detector using preparative silica or semipreparative C18 ((250 × 21.2 mm, 5 μm) or (250 × 10.0 mm, 5 μm)) conditions.

### Biological Materials

Four specimens of *Duva florida* (Cnidaria, Alcyonacea, Alcyoniina, Neptheidae) were collected at a depth of 823 m along the Irish continental margin (54.26007932 N, 11.58046619 W) during a 2018 cruise using the ROV *Holland I* deployed from the Irish national research vessel R/V *Celtic Explorer*. Specimens were stored in bioboxes on the ROV and immediately pooled, logged, labeled, and frozen at −80 °C when the ROV was recovered to the vessel. Specimens were freeze-dried on return to land and then stored until analysis at −20 °C.

### Extraction and Isolation

Following a Soxhlet extraction in CHCl_2_ of 88 g of four lyophilized organisms, 14.3 g of crude extract was obtained and underwent a liquid/liquid partition with CH_2_Cl_2_/H_2_O, resulting in 13.7 g of extract in the organic layer. A crude separation was achieved utilizing NP MPLC with a gradient from 1% to 100% EtOAc in hexanes over 30 min followed by a 20% MeOH in EtOAc wash following a period of 100% EtOAc for 8 min on a silica column. All fractions were dried under either passive air or nitrogen, and fractions 5–9, eluting from roughly 20–50% EtOAc, were prioritized for further purification based on comparisons of the ^1^H NMR spectra to that of the known tuaimenal A (**10**). Crude MPLC fractions of interest underwent iterative rounds of normal and reversed-phase HPLC until pure compounds were achieved as determined from ^1^H and ^13^C NMR spectra, as well as MS, analyses.

### Stereochemical Evaluation

Optical rotation data were acquired in MeOH or CHCl_3_ using an AutoPol IV polarimeter at a wavelength of 589 nm with a 10 mm path length cell. Chiral-phase chromatography was performed on a semipreparative Phenomenex Lux Amylose-3 column utilizing a gradient from 50% to 100% MeOH in H_2_O over 30 min with results recorded at a wavelength of 254 nm and integrations performed using Shimadzu LabSolutions software.

### X-ray Crystallography

Crystals of **8** were obtained from a concentrated solution of MeOH in a refrigerator with limited oxygen exchange into or out of the sample vial to slow the progression of crystal growth for a period of 5 days. A Bruker D8 Venture PHOTON II CPAD diffractometer with a Cu Kα INOCOATEC ImuS microfocus source was used to measure C-ray diffraction. The Difference Vectors method APEX3 was utilized for indexing with SaintPlus employed for data integration and reduction. Multiscan methods in SADABS were employed for absorption correction, and XPREP in APEX3 was used for space group determination. SHELXT was used to solve the structure with refinements from SHELXL-2018 in an OLEX2 interface program. A riding model with isotropic thermal parameters was utilized for the placement of hydrogen atoms prior to refinement in geometrically calculated positions.

Crystallographic data for the structure reported in this article (**8**) were deposited at the Cambridge Crystallographic Data Centre under the deposition number CCDC 2207359. These data can be obtained free of charge via www.ccdc.cam.ac.uk/data_request/cif.

### Cell Culture

The cervical cancer cell lines CaSki and C33A were cultured in Rosewell Park Memorial Institute (RPMI) 1640 (1×) complete media and Dulbecco’s Modified Eagle Medium (DMEM), respectively, supplemented with 10% fetal bovine serum (FBS), 100 units/mL of penicillin, 100 mg/mL of streptomycin, 2 mM l-glutamines, 1 mM sodium pyruvate, 0.1 mM nonessential amino acids (NEAA), 0.05 mM 2-mercaptoethanol, 0.5 mg/mL amphotericin B, and 0.5 mg/mL gentamycin. CaSki (ATCC, CRM-CRL-1550) and C33A (ATCC, HTB-31) cell cultures were kept at passages less than 10 and maintained in an incubator at 37 °C and 5% CO_2_ atmosphere.

### Analysis of Cellular Proliferation

Cells were seeded in a 96-well plate at 1000–3000 cells per well with 200 μL of media to achieve 50% confluency. Cells were treated with dimethyl sulfoxide (DMSO negative control, *N* = 3), 50 μM etoposide (positive control, *N* = 3), and serial dilutions of tuaimenal A (**10**) and its derivatives B, F, G, and H (**1**, **5**–**7**) (200, 133, 89, 59, 40, 26, 18, 12, 8, 5, 4, and 2 μM, *N* = 3) diluted in DMSO. After 48 h of treatment, the cell proliferation was analyzed using water-soluble tetrazolium (WST-1, Roche, 5015944001) at wavelengths of 450 and 600 nm. The plates were read on a BioTek SynergyHT microplate reader. End point absorbances (450–600 nm) were normalized, and nonlinear regression curves were plotted and fitted using GraphPad Prism software.

#### Tuaimenal B (**1**):

pale orange oil, [α]^25^_D_ 0.0 (*c* 0.021, CHCl_3_); UV (C_2_H_3_N) λ_max_ (log ε) 222 nm (6.45); IR ν (thin film) 3426, 2972, 2928, 2861, 1774, 1744, 1588, 1461, 1379, 1215, 1089, 1029 cm^–1^; ^1^H and ^13^C NMR data, see Table 1; HRESIMS *m*/*z* 383.2241 [M – H]^−^ (calcd for C_24_H_32_O_4_, 383.2228).

#### Tuaimenal C (**2**):

pale yellow oil, [α]^25^_D_ 0.0 (*c* 0.008, CHCl_3_); UV (C_2_H_3_N) λ_max_ (log ε) 227 nm (6.35); IR ν (thin film) 3508, 2928, 2861, 1766, 1580, 1461, 1379, 1215, 1096, 1029 cm^–1^; ^1^H and ^13^C NMR data, see Table 2; 70 eV HREIMS *m*/*z* 398.2452 [M]^+^ (calcd for C_25_H_34_O_4_, 398.2457).

#### Tuaimenal D (**3**):

pale orange oil, [α]^25^_D_ 1.1 (*c* 0.018, CHCl_3_); UV (C_2_H_3_N) λ_max_ (log ε) 250 nm (6.38); IR ν (thin film) 3397, 2972, 2935, 1729, 1617, 1429, 1379, 1342, 1290, 1171, 1126, 1059 cm^–1^; ^1^H and ^13^C NMR data, see Table 2; HRESIMS *m*/z 387.2197 [M – H]^−^ (calcd for C_23_H_32_O_5_, 387.2177).

#### Tuaimenal E (**4**):

pale orange oil, [α]^25^_D_ 2.6 (*c* 0.034, CHCl_3_); UV (C_2_H_3_N) λ_max_ (log ε) 250 nm (6.55); IR ν (thin film) 3419, 2980, 2935, 2876, 1729, 1640, 1491, 1439, 1379, 1342, 1282, 1171, 1134, 1059 cm^–1^; ^1^H and ^13^C NMR data, see Table 2; HRESIMS *m*/*z* 429.2296 [M – H]^−^ (calcd for C_25_H_34_O_6_, 429.2283).

#### Tuaimenal F (**5**):

yellow oil, [α]^25^_D_ 1.5 (*c* 0.059, CHCl_3_); UV (C_2_H_3_N) λ_max_ (log ε) 228 nm (6.53); IR ν (thin film) 3374, 2972, 2943, 1647, 1588, 1461, 1431, 1394, 1208, 1104, 1029 cm^–1^; ^1^H and ^13^C NMR data, see Table 2; HRESIMS *m*/*z* 373.2399 [M – H]^−^ (calcd for C_23_H_34_O_4_, 373.2384).

#### Tuaimenal G (**6**):

yellow oil, [α]^25^_D_ 1.1 (*c* 0.065, CHCl_3_); UV (C_2_H_3_N) λ_max_ (log ε) 230 nm (6.58); IR ν (thin film) 3426, 2972, 2935, 1737, 1707, 1588, 1461, 1431, 1372, 1260, 1208, 1104, 1029 cm^–1^; ^1^H and ^13^C NMR data, see Table 2; HRESIMS *m*/*z* 439.2455 [M – H]^−^ (calcd for C_25_H_36_O_5_, 439.2455).

#### Tuaimenal H (**7**):

pale orange film, UV (C_2_H_3_N) λ_max_ (log ε) 299 nm (6.44); IR ν (thin film) 3374, 2972, 2920, 2861, 1617, 1439, 1335, 1268, 1178, 1052 cm^–1^; ^1^H and ^13^C NMR data, see Table 2; HRESIMS *m*/*z* 371.2245 [M – H]^−^ (calcd for C_23_H_32_O_4_, 371.2228).

#### 3-Hydroxy-19-norchola-1,3,5^10^-trien-24-oic acid (**8**):

white crystalline solid, [α]^25^_D_ 44.4 (*c* 0.024, CHCl_3_); UV (C_2_H_3_N) λ_max_ (log ε) 281 nm (6.00); IR ν (thin film) 3255, 2935, 2868, 1707, 1617, 1506, 1454, 1387, 1290, 1245, 1186, 1104, 1029 cm^–1^; ^13^C NMR data, see Table S2; HRESIMS *m*/*z* 355.2298 [M – H]^−^ (calcd for C_23_H_32_O_3_, 355.2279).

#### Methyl 3-hydroxy-19-norchola-1,3,5^10^-trien-24-oic acid (**9**):

white crystalline solid, [α]^25^_D_ 45.1 (*c* 0.065, CHCl_3_); UV (C_2_H_3_N) λ_max_ (log ε) 280 nm (6.02); IR ν (thin film) 3426, 2943, 2868, 1744, 1617, 1506, 1446, 1387, 1290, 1253, 1186 cm^–1^; ^13^C NMR data, see Table S2; HRESIMS *m*/*z* 371.2589 [M + H]^+^ (calcd for C_24_H_34_O_3_, 371.2581).

#### Tuaimenal A (**10**):

yellow oil, [α]^25^_D_ 0.5 (*c* 0.058, CHCl_3_); UV (C_2_H_3_N) λ_max_ (log ε) 253 nm (6.37); IR ν (thin film) 3412, 2972, 2928, 1610, 1491, 1439, 1335, 1282, 1171, 1119, 1052 cm^–1^; ^1^H and ^13^C NMR data, see Table S1; HRESIMS *m*/*z* 369.2089 [M – H]^−^ (calcd for C_23_H_30_O_4_, 369.2071).

## Supporting Information Available

NMR spectra of tuaimenals B–H (**1**–**7**), 3-hydroxy-19-norchola-1,3,5^10^-trien-24-oic acid (**8**), methyl 3-hydroxy-19-norchola-1,3,5^10^-trien-24-oic acid (**9**), and tuaimenal A (**10**); HRESIMS of **1**, **3**–**10**; HREIMS of **2**; UV λ_max_ of **1**–**10**; IR spectra of **1**–**10**; X-ray crystallographic data for **8**; comparisons to literature ^1^H and ^13^C spectra of **8**–**10**, chiral-phase HPLC chromatogram of **4** (PDF)

## References

[ref1] Newman D. J.; Cragg G. M. J. Nat. Prod. 2020, 83, 770–803. 10.1021/acs.jnatprod.9b01285. 32162523

[ref2] Skropeta D. Nat. Prod. Rep. 2008, 25, 1131–166. 10.1039/b808743a. 19030606

[ref3] Travis J. Science 1993, 259, 1123–1124. 10.1126/science.259.5098.1123. 17794391

[ref4] Skropeta D.; Wei L. Nat. Prod. Rep. 2014, 31, 1–27. 10.1039/C3NP70118B. 24871201

[ref5] Burd E. M. Clin. Microbiol. Rev. 2003, 16, 1–17. 10.1128/CMR.16.1.1-17.2003. 12525422 PMC145302

[ref6] Bedell A. L; Goldstein L. S; Goldstein A. R; Goldstein A. T. Med. Rev. 2020, 8, 28–37. 10.1016/j.sxmr.2019.09.005. 31791846

[ref7] Canfell K. Papillomavirus Res. 2019, 8, 1–3. 10.1016/j.pvr.2019.100170. PMC6722296 31176807

[ref8] Arbyn M.; Weiderpass E.; Bruni L.; Sanjose S. D; Saraiya M.; Ferlay J.; Bray F. Lancet Glob. Health. 2020, 8, 191–203. 10.1016/S2214-109X(19)30482-6. PMC7025157 31812369

[ref9] Merza J.; Aumond M. C; Rondeau D.; Dumontet V.; Le Ray A. M; Seraphin D.; Richomme P. Phytochemistry 2004, 65, 2915–2920. 10.1016/j.phytochem.2004.06.037. 15501261

[ref10] Avalon N. E; Nafie J.; Verissimo C. D. M.; Warrensford L. C.; Dietrick S. G.; Pittman A. R.; Young R. M.; Kearns F. L.; Smalley T.; Binning J. M.; Dalton J. P.; Johnson M. P.; Woodcock H. L; Allcock A. L.; Baker B. J. J. Nat. Prod. 2022, 85, 1315–1323. 10.1021/acs.jnatprod.2c00054. 35549259 PMC9127705

[ref11] Reher R.; Kim H. W.; Zhang C.; Mao H. H.; Wang M.; Nothias L.-F.; Caraballo-Rodriguez A. M.; Glukhov E.; Teke B.; Leao T.; Alexander K. L.; Duggan B. M.; Van Everbroeck E. L.; Dorrestein P. C.; Cottrell G. W.; Gerwick W. H. J. Am. Chem. Soc. 2020, 142, 4114–4120. 10.1021/jacs.9b13786. 32045230 PMC7210566

[ref12] Lu Z.; Van Wagoner R. M.; Harper M. K.; Hooper J. N. A.; Ireland C. M. Nat. Prod. Commun. 2010, 5, 1571–1574. 10.1177/1934578X1000501011. 21121250 PMC3050653

[ref13] Birringer M.; Siems K.; Maxones A.; Frank J.; Lorkowski S. RSC Adv. 2018, 8, 4803–4841. 10.1039/C7RA11819H. 35539527 PMC9078042

