# Supplementary material for: Tuaimenals B–H, Merosesquiterpenes from the Irish Deep-Sea Soft Coral Duva florida with Bioactivity against Cervical Cancer Cell Lines
Source: J Nat Prod. 2022 Dec 29;86(1):182–90. doi: 10.1021/acs.jnatprod.2c00898 (PMC9887596; doi:10.1021/acs.jnatprod.2c00898)
Supplement: Supplementary file 1 — np2c00898_si_001.pdf [file np2c00898_si_001.pdf]

Supplemental Information for:

Tuaimenals B-H, Merosesquiterpenes from the Irish Deep-Sea Soft Coral *Duva florida* with Bioactivity Against Cervical Cancer Cell Lines

Joshua T. Welsch,[†] Tracess B. Smalley,[‡] Jenet K. Matlack,[‡] Nicole E. Avalon,[†] Jennifer M. Binning,[‡] Mark P. Johnson,[§] A. Louise Allcock,[§] Bill J. Baker[†]

[†] Department of Chemistry, University of South Florida, Tampa, FL, USA.

[‡] Molecular Oncology, Moffitt Cancer Research Center, Tampa, FL, USA.

[§] Department of Zoology, National University of Ireland Galway, Galway, Ireland.

	Page
Figure S1: Tuaimenal B (**1**) ^{1}H NMR spectrum (600 MHz, $CDCl_3$).	4
Figure S2: Tuaimenal B (**1**) ^{13}C NMR spectrum (150 MHz, $CDCl_3$).	5
Figure S3: Tuaimenal B (**1**) HSQC NMR spectrum (500 MHz, $CDCl_3$).	5
Figure S4: Tuaimenal B (**1**) HMBC NMR spectrum (500 MHz, $CDCl_3$).	6
Figure S5: Tuaimenal B (**1**) COSY NMR spectrum (500 MHz, $CDCl_3$).	6
Figure S6: Tuaimenal B (**1**) NOESY NMR spectrum (500 MHz, $CDCl_3$).	7
Figure S7: Tuaimenal B (**1**) zoomed NOESY NMR spectrum (500 MHz, $CDCl_3$).	7
Figure S8: Tuaimenal B (**1**) HRESIMS (neg).	8
Figure S9: Tuaimenal B (**1**) UV λ_{max} (C_2H_3N).	8
Figure S10: Tuaimenal B (**1**) IR spectrum (thin film).	9
Figure S11: Tuaimenal C (**2**) ^{1}H NMR spectrum (800 MHz, $CDCl_3$).	9
Figure S12: Tuaimenal C (**2**) ^{13}C NMR spectrum (200 MHz, $CDCl_3$).	10
Figure S13: Tuaimenal C (**2**) HSQC NMR spectrum (800 MHz, $CDCl_3$).	10
Figure S14: Tuaimenal C (**2**) HMBC NMR spectrum (800 MHz, $CDCl_3$).	11
Figure S15: Tuaimenal C (**2**) COSY NMR spectrum (800 MHz, $CDCl_3$).	11
Figure S16: Tuaimenal C (**2**) NOESY NMR spectrum (800 MHz, $CDCl_3$).	12

Figure S17: Tuaimenal C (**2**) zoomed NOESY NMR spectrum (800 MHz, $CDCl_3$). 12
Figure S18: Tuaimenal C (**2**) HREIMS. 13
Figure S19: Tuaimenal C (**2**) UV λ_{max} (C_2H_3N). 13
Figure S20: Tuaimenal C (**2**) IR spectrum (thin film). 14
Figure S21: Tuaimenal D (**3**) ^1H NMR spectrum (600 MHz, $CDCl_3$). 14
Figure S22: Tuaimenal D (**3**) ^{13}C NMR spectrum (150 MHz, $CDCl_3$). 15
Figure S23: Tuaimenal D (**3**) ^{13}C NMR spectrum zoomed (150 MHz, $CDCl_3$). 15
Figure S24: Tuaimenal D (**3**) HSCQ NMR spectrum (500 MHz, $CDCl_3$). 16
Figure S25: Tuaimenal D (**3**) HMBC NMR spectrum (500 MHz, $CDCl_3$). 16
Figure S26: Tuaimenal D (**3**) COSY NMR spectrum (500 MHz, $CDCl_3$). 17
Figure S27: Tuaimenal D (**3**) NOESY NMR spectrum (500 MHz, $CDCl_3$). 17
Figure S28: Tuaimenal D (**3**) zoomed NOESY NMR spectrum (500 MHz, $CDCl_3$). 18
Figure S29: Tuaimenal D (**3**) HRESIMS (neg). 18
Figure S30: Tuaimenal D (**3**) UV λ_{max} (C_2H_3N). 19
Figure S31: Tuaimenal D (**3**) IR spectrum (thin film). 19
Figure S32: Tuaimenal E (**4**) ^1H NMR spectrum (600 MHz, $CDCl_3$). 20
Figure S33: Tuaimenal E (**4**) ^{13}C NMR spectrum (150 MHz, $CDCl_3$). 20
Figure S34: Tuaimenal E (**4**) HSCQ NMR spectrum (500 MHz, $CDCl_3$). 21
Figure S35: Tuaimenal E (**4**) HMBC NMR spectrum (500 MHz, $CDCl_3$). 21
Figure S36: Tuaimenal E (**4**) COSY NMR spectrum (500 MHz, $CDCl_3$). 22
Figure S37: Tuaimenal E (**4**) NOESY NMR spectrum (500 MHz, $CDCl_3$). 22
Figure S38: Tuaimenal E (**4**) zoomed NOESY NMR spectrum (500 MHz, $CDCl_3$). 23
Figure S39: Tuaimenal E (**4**) HRESIMS (neg). 23
Figure S40: Tuaimenal E (**4**) UV λ_{max} (C_2H_3N). 24
Figure S41: Tuaimenal E (**4**) IR spectrum (thin film). 24
Figure S42: Tuaimenal F (**5**) ^1H NMR spectrum (600 MHz, $CDCl_3$). 25
Figure S43: Tuaimenal F (**5**) ^{13}C NMR spectrum (150 MHz, $CDCl_3$). 25
Figure S44: Tuaimenal F (**5**) HSCQ NMR spectrum (500 MHz, $CDCl_3$). 26
Figure S45: Tuaimenal F (**5**) HMBC NMR spectrum (500 MHz, $CDCl_3$). 26
Figure S46: Tuaimenal F (**5**) COSY NMR spectrum (500 MHz, $CDCl_3$). 27
Figure S47: Tuaimenal F (**5**) NOESY NMR spectrum (500 MHz, $CDCl_3$). 27
Figure S48: Tuaimenal F (**5**) zoomed NOESY NMR spectrum (500 MHz, $CDCl_3$). 28
Figure S49: Tuaimenal F (**5**) HRESIMS (neg). 28
Figure S50: Tuaimenal F (**5**) UV λ_{max} (C_2H_3N). 29
Figure S51: Tuaimenal F (**5**) IR spectrum (thin film). 29
Figure S52: Tuaimenal G (**6**) ^1H NMR spectrum (600 MHz, $CDCl_3$). 30
Figure S53: Tuaimenal G (**6**) ^{13}C NMR spectrum (150 MHz, $CDCl_3$). 30
Figure S54: Tuaimenal G (**6**) HSCQ NMR spectrum (500 MHz, $CDCl_3$). 31
Figure S55: Tuaimenal G (**6**) HMBC NMR spectrum (500 MHz, $CDCl_3$). 31
Figure S56: Tuaimenal G (**6**) COSY NMR spectrum (500 MHz, $CDCl_3$). 32
Figure S57: Tuaimenal G (**6**) NOESY NMR spectrum (500 MHz, $CDCl_3$). 32
Figure S58: Tuaimenal G (**6**) zoomed NOESY NMR spectrum (500 MHz, $CDCl_3$). 33
Figure S59: Tuaimenal G (**6**) HRESIMS (pos). 33
Figure S60: Tuaimenal G (**6**) UV λ_{max} (C_2H_3N). 34
Figure S61: Tuaimenal G (**6**) IR spectrum (thin film). 34
Figure S62: Tuaimenal H (**7**) ^1H NMR spectrum (600 MHz, $CDCl_3$). 35

Figure S63: Tuaimenal H (**7**) ^{13}C NMR spectrum (150 MHz, $CDCl_3$). 35
Figure S64: Tuaimenal H (**7**) HSCQ NMR spectrum (500 MHz, $CDCl_3$). 36
Figure S65: Tuaimenal H (**7**) HMBC NMR spectrum (500 MHz, $CDCl_3$). 36
Figure S66: Tuaimenal H (**7**) COSY NMR spectrum (500 MHz, $CDCl_3$). 37
Figure S67: Tuaimenal H (**7**) NOESY NMR spectrum (500 MHz, $CDCl_3$). 37
Figure S68: Tuaimenal H (**7**) zoomed NOESY NMR spectrum (500 MHz, $CDCl_3$). 38
Figure S69: Tuaimenal H (**7**) HRESIMS (neg). 38
Figure S70: Tuaimenal H (**7**) UV λ_{max} (C_2H_3N). 39
Figure S71: Tuaimenal H (**7**) IR spectrum (thin film). 39
Figure S72: 3-hydroxy-19-norchola-1,3,5(10)-trien-24-oic acid (**8**) ^{1}H NMR spectrum (600 MHz, MeOD). 40
Figure S73: 3-hydroxy-19-norchola-1,3,5(10)-trien-24-oic acid (**8**) ^{13}C NMR spectrum (150 MHz, MeOD). 40
Figure S74: 3-hydroxy-19-norchola-1,3,5(10)-trien-24-oic acid (**8**) HSCQ NMR spectrum (500 MHz, MeOD). 41
Figure S75: 3-hydroxy-19-norchola-1,3,5(10)-trien-24-oic acid (**8**) HMBC NMR spectrum (500 MHz, MeOD). 41
Figure S76: 3-hydroxy-19-norchola-1,3,5(10)-trien-24-oic acid (**8**) COSY NMR spectrum (500 MHz, MeOD). 42
Figure S77: 3-hydroxy-19-norchola-1,3,5(10)-trien-24-oic acid (**8**) NOESY NMR spectrum (500 MHz, MeOD). 42
Figure S78: 3-hydroxy-19-norchola-1,3,5(10)-trien-24-oic acid (**8**) HRESIMS (neg). 43
Figure S79: 3-hydroxy-19-norchola-1,3,5(10)-trien-24-oic acid (**8**) UV λ_{max} (C_2H_3N). 43
Figure S80: 3-hydroxy-19-norchola-1,3,5(10)-trien-24-oic acid (**8**) IR spectrum (thin film). 44
Figure S81: 3-hydroxy-19-norchola-1,3,5(10)-trien-24-oic acid (**8**) X-ray crystal structure. 44
Figure S82: 3-hydroxy-19-norchola-1,3,5(10)-trien-24-oic acid (**8**) X-ray crystallography metadata and ellipsoid plot with anisotropic displacement parameters drawn at 50% probability. 45
Figure S83: Methyl 3-hydroxy-19-norchola-1,3,5(10)-trien-24-oic acid (**9**) ^{1}H NMR spectrum (600 MHz, $CDCl_3$). 46
Figure S84: Methyl 3-hydroxy-19-norchola-1,3,5(10)-trien-24-oic acid (**9**) ^{13}C NMR spectrum (150 MHz, $CDCl_3$). 46
Figure S85: Methyl 3-hydroxy-19-norchola-1,3,5(10)-trien-24-oic acid (**9**) HSCQ NMR spectrum (500 MHz, $CDCl_3$). 47
Figure S86: Methyl 3-hydroxy-19-norchola-1,3,5(10)-trien-24-oic acid (**9**) HMBC NMR spectrum (500 MHz, $CDCl_3$). 47
Figure S87: Methyl 3-hydroxy-19-norchola-1,3,5(10)-trien-24-oic acid (**9**) COSY NMR spectrum (500 MHz, $CDCl_3$). 48
Figure S88: Methyl 3-hydroxy-19-norchola-1,3,5(10)-trien-24-oic acid (**9**) NOESY NMR spectrum (500 MHz, $CDCl_3$). 48
Figure S89: Methyl 3-hydroxy-19-norchola-1,3,5(10)-trien-24-oic acid (**9**) HRESIMS (pos). 49

Figure S90: 19-norchola-1,3,5(10)-trien-24-oic acid, 3-hydroxy-, methyl ester (**9**) UV λ_{max} (C_2H_3N). 49
Figure S91: 19-norchola-1,3,5(10)-trien-24-oic acid, 3-hydroxy-, methyl ester **9**) IR spectrum (thin film). 50
Figure S92: Tuaimenal A (**10**) ^{1}H NMR spectrum (600 MHz, $CDCl_3$). 50
Figure S93: Tuaimenal A (**10**) ^{13}C NMR spectrum (150 MHz, $CDCl_3$). 51
Figure S94: Tuaimenal A (**10**) HSCQ NMR spectrum (500 MHz, $CDCl_3$). 51
Figure S95: Tuaimenal A (**10**) HMBC NMR spectrum (500 MHz, $CDCl_3$). 52
Figure S96: Tuaimenal A (**10**) COSY NMR spectrum (500 MHz, $CDCl_3$). 52
Figure S97: Tuaimenal A (**10**) NOESY NMR spectrum (500 MHz, $CDCl_3$). 53
Figure S98: Tuaimenal A (**10**) zoomed NOESY NMR spectrum (500 MHz, $CDCl_3$). 53
Figure S99: Tuaimenal A (**10**) HRESIMS (neg). 54
Figure S100: Tuaimenal A (**10**) UV λ_{max} (C_2H_3N). 54
Figure S101: Tuaimenal A (**10**) IR spectrum (thin film). 55
Table S1: Comparison of tuaimenal A (**10**) ^{1}H and ^{13}C spectra to literature. 56
Table S2: Comparison of *Duva florida* steroids (**8** and **9**) ^{13}C NMR spectra to literature. 57
Figure S102: Integrated chromatogram displaying relative abundance of *R* and *S* enantiomers of tuaimenal E (**4**) separated utilizing a chiral HPLC column. 58

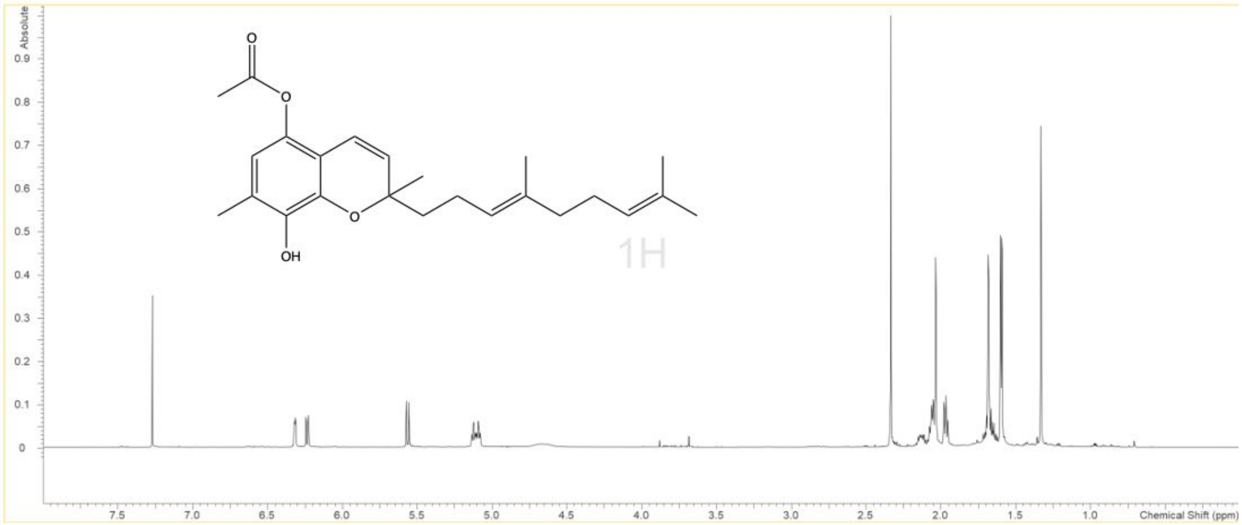

Figure S1: Tuaimenal B (**1**) ^{1}H NMR spectrum (600 MHz, $CDCl_3$).

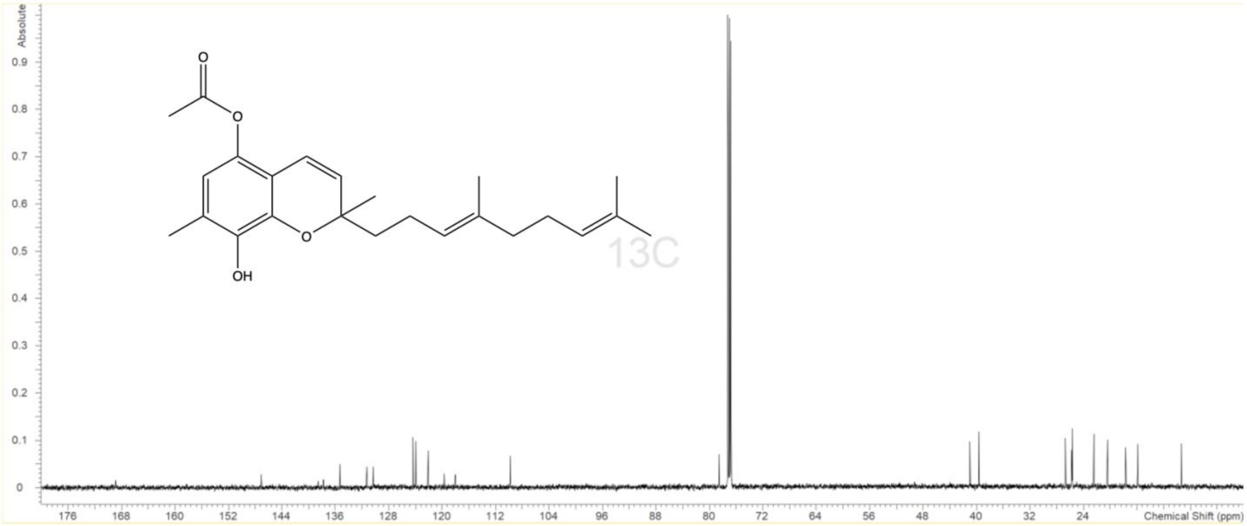

Figure S2: Tuaimenal B (**1**) ^{13}C NMR spectrum (150 MHz, $CDCl_3$).

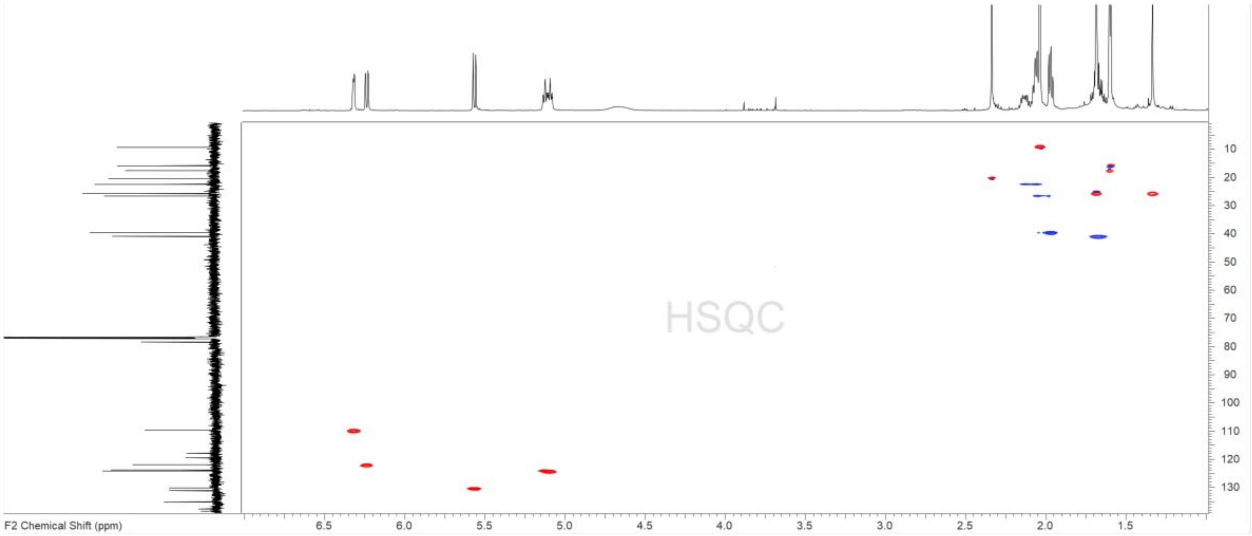

Figure S3: Tuaimenal B (**1**) HSQC NMR spectrum (500 MHz, $CDCl_3$).

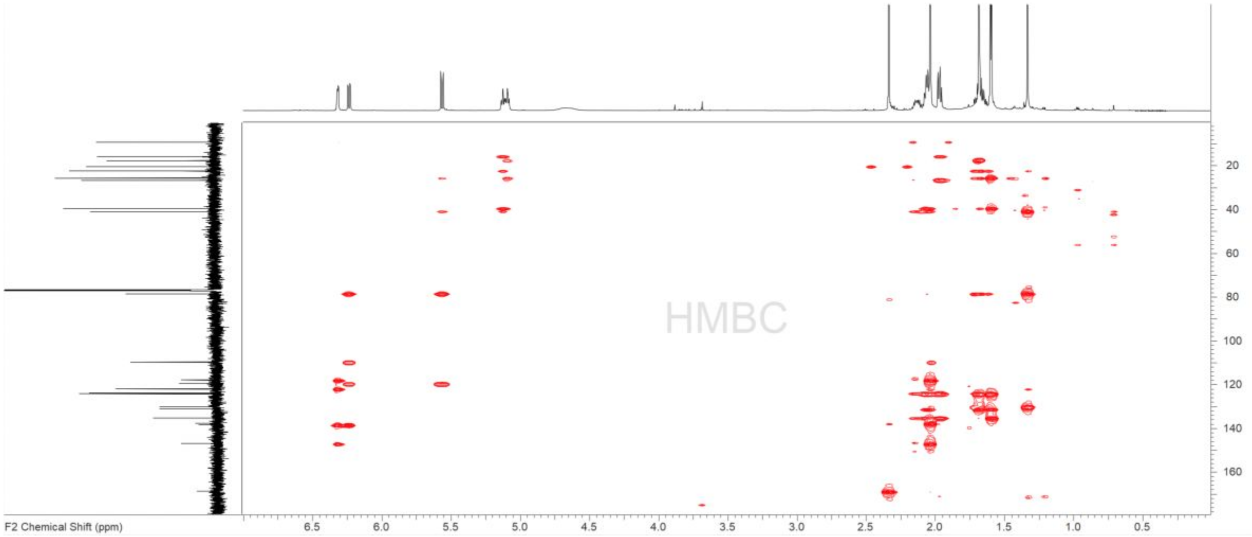

Figure S4: Tuaimenal B (**1**) HMBC NMR spectrum (500 MHz, $CDCl_3$).

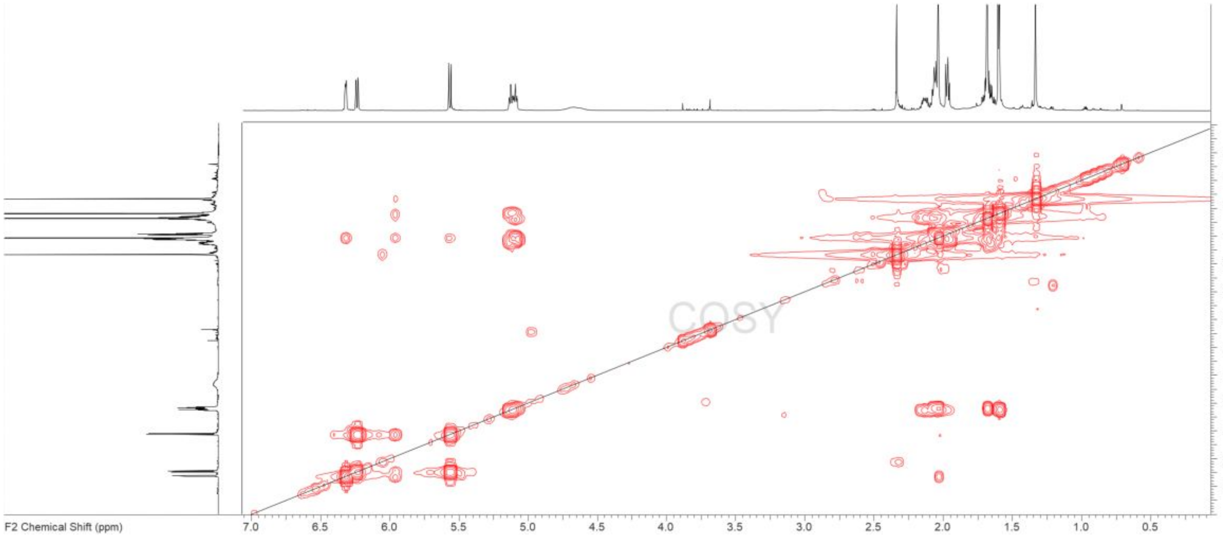

Figure S5: Tuaimenal B (**1**) COSY NMR spectrum (500 MHz, $CDCl_3$).

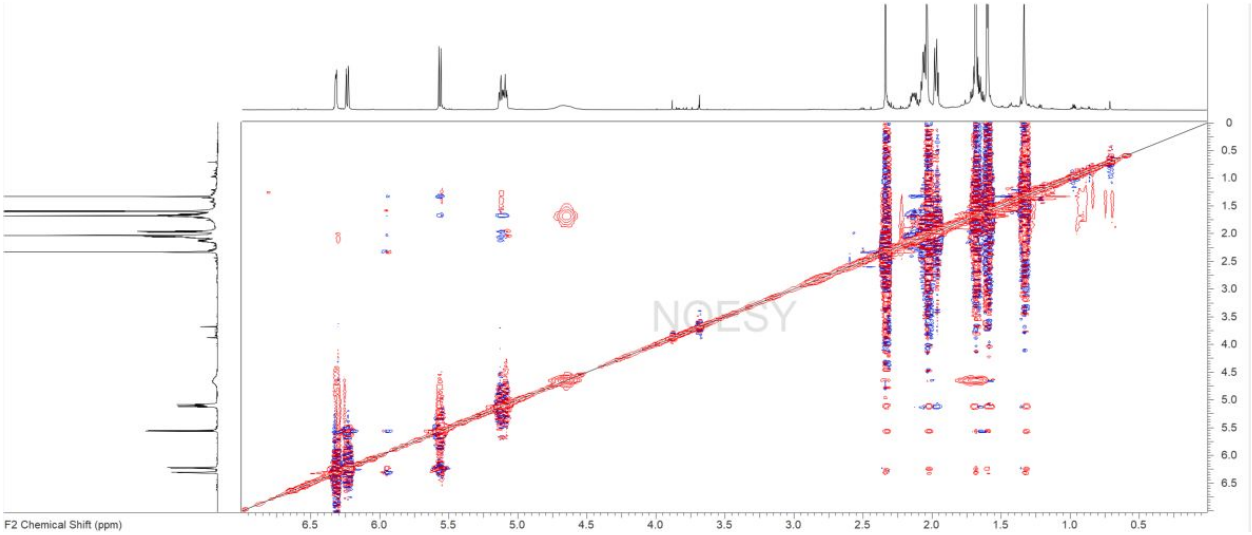

Figure S6: Tuaimenal B (**1**) NOESY NMR spectrum (500 MHz, $CDCl_3$).

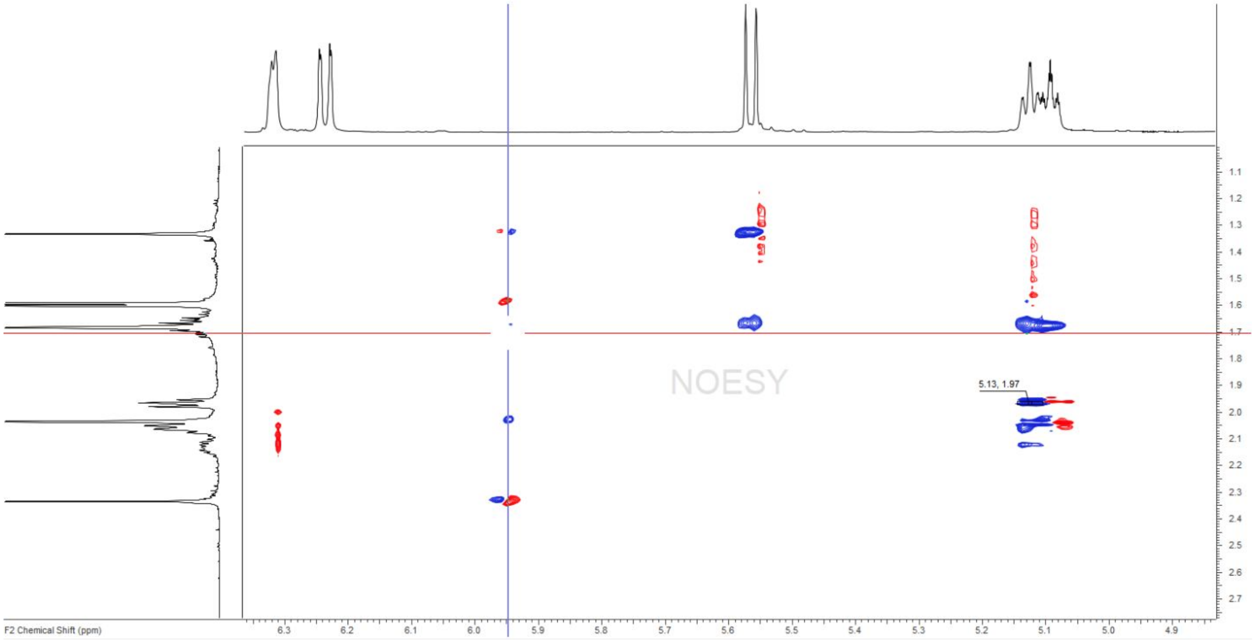

Figure S7: Tuaimenal B (**1**) zoomed NOESY NMR spectrum (500 MHz, $CDCl_3$).

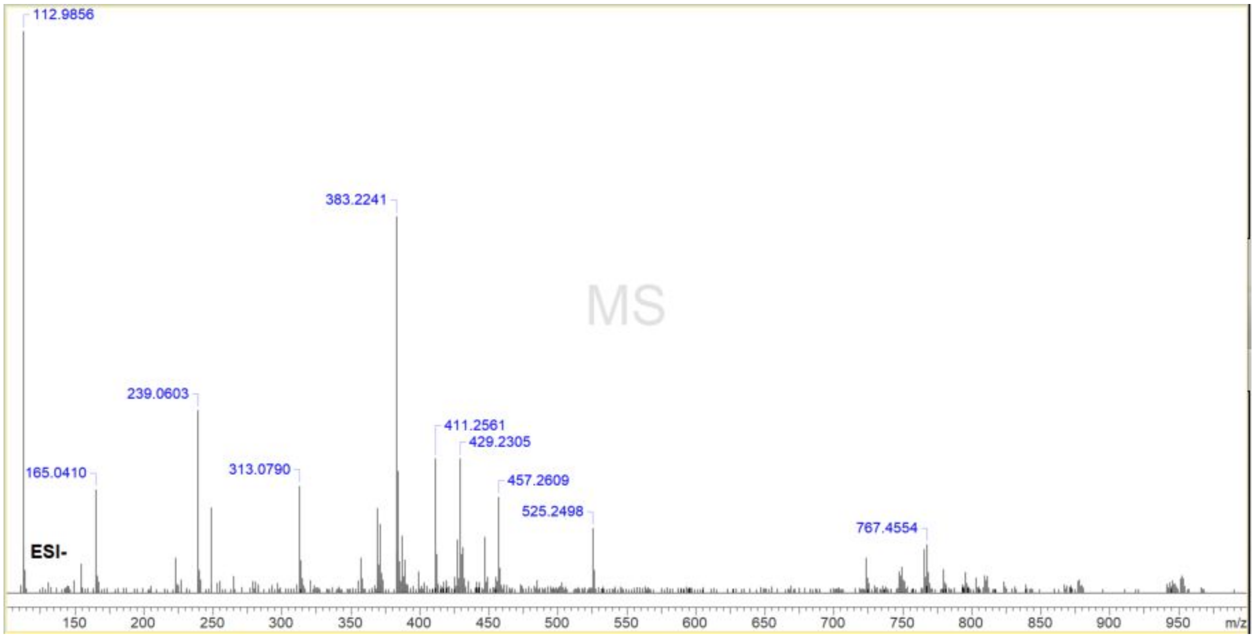

Figure S8: Tuaimenal B (**1**) HRESIMS (neg).

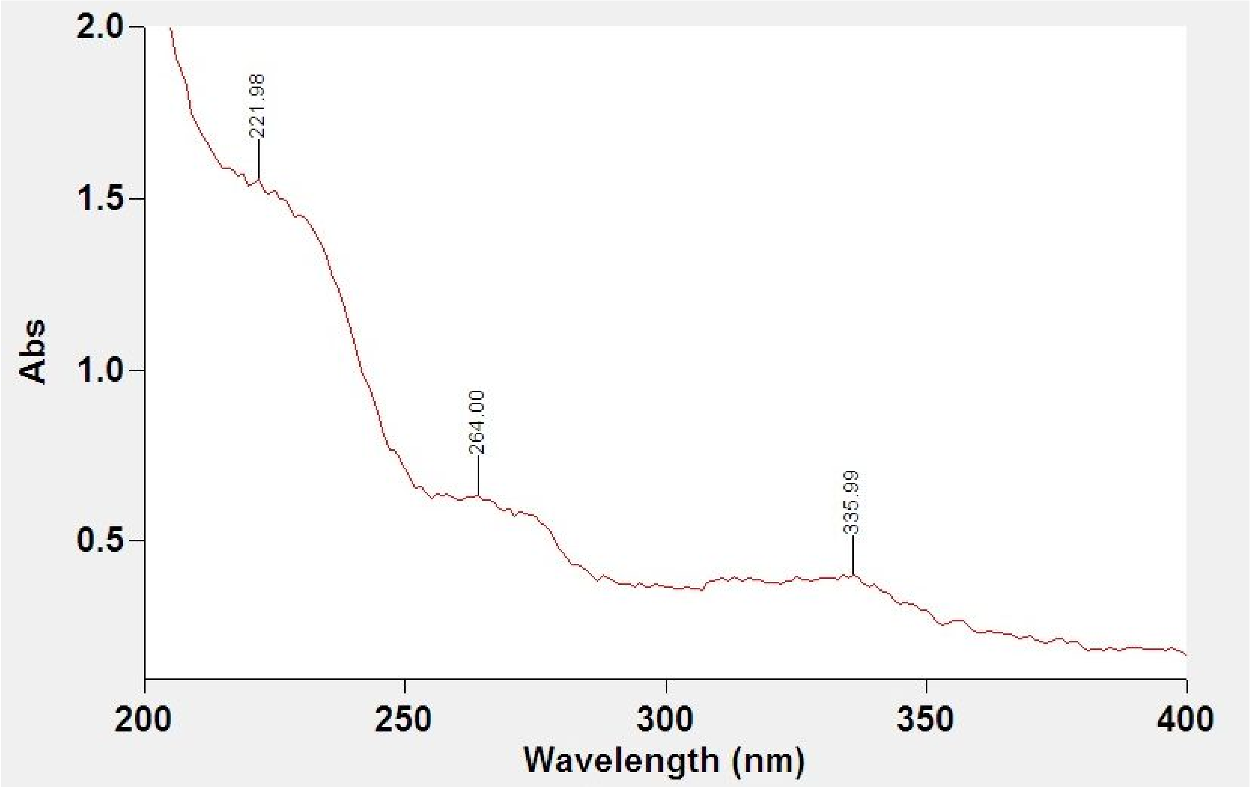

Figure S9: Tuaimenal B (**1**) UV λ_{max} (C_2H_3N).

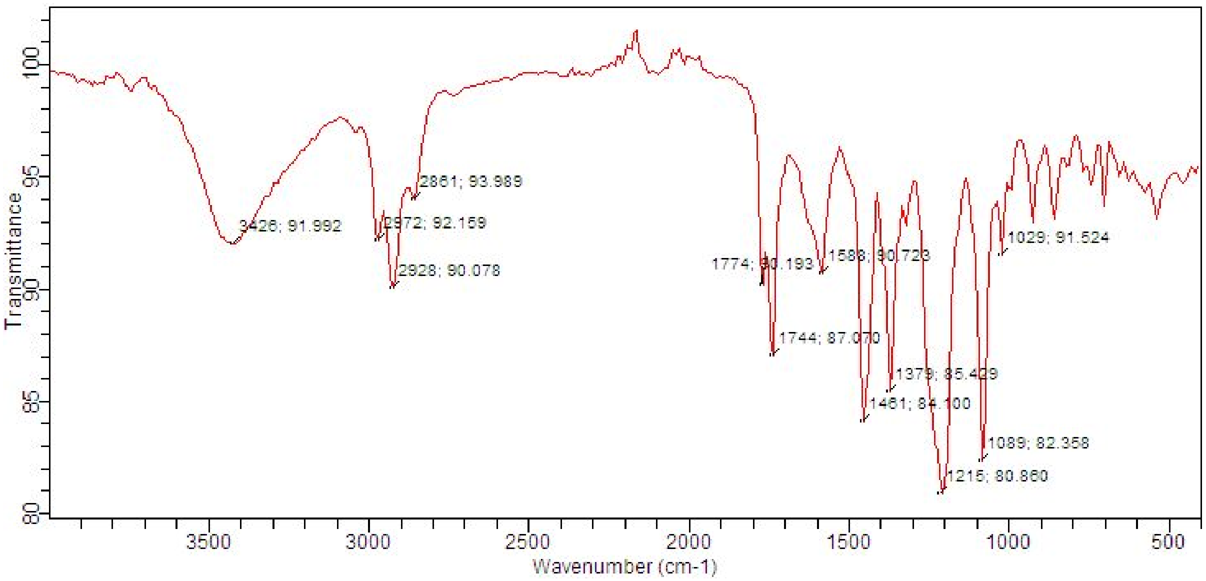

Figure S10: Tuaimenal B (**1**) IR spectrum (thin film).

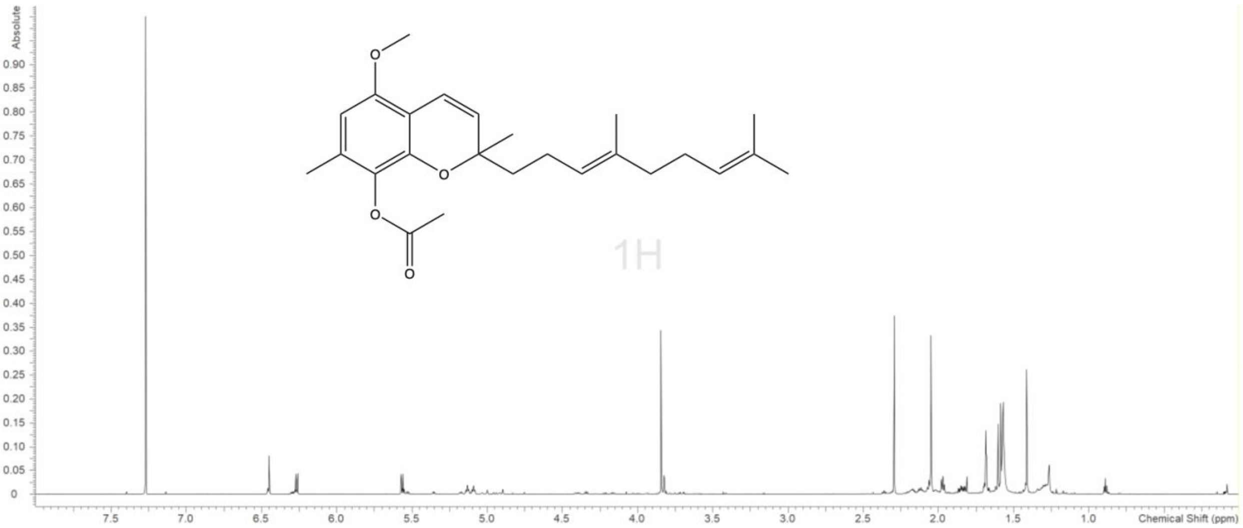

Figure S11: Tuaimenal C (**2**) ^{1}H NMR spectrum (800 MHz, $CDCl_3$).

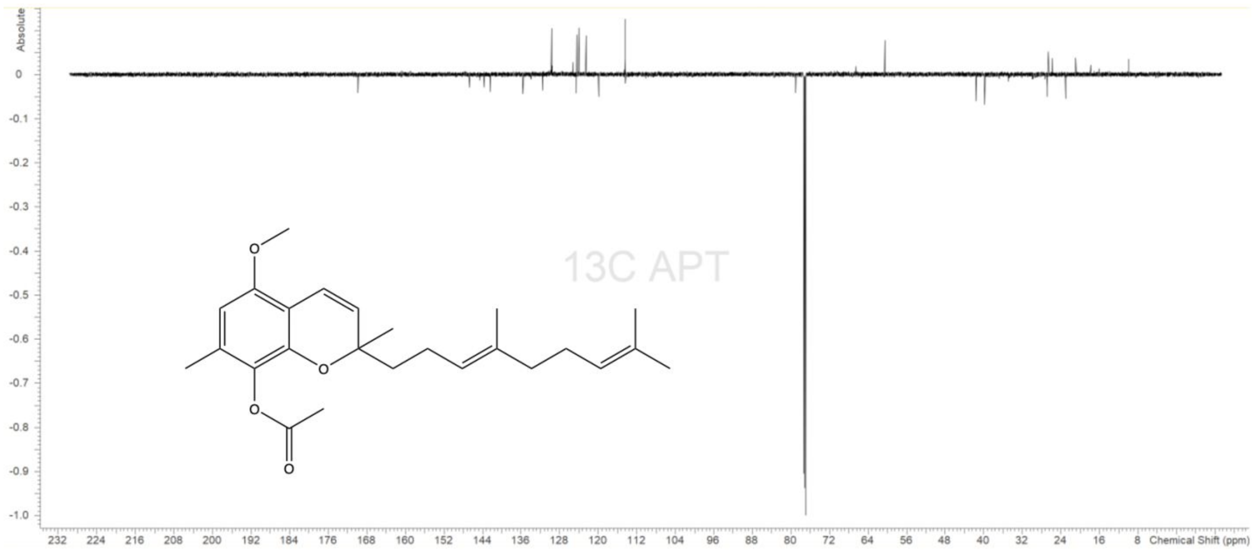

Figure S12: Tuaimenal C (**2**) ^{13}C NMR spectrum (200 MHz, $CDCl_3$).

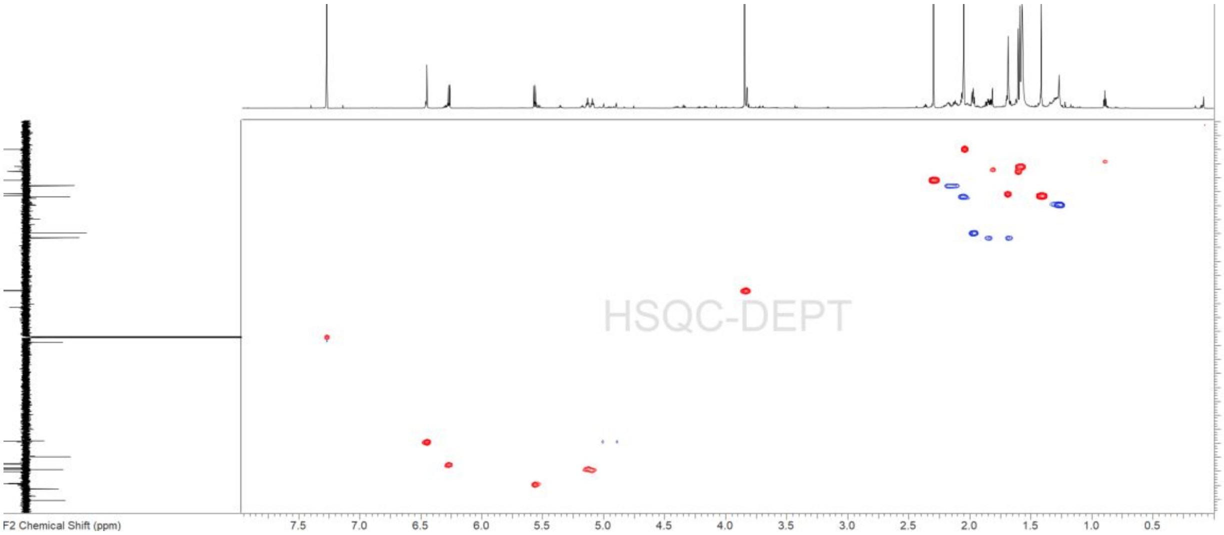

Figure S13: Tuaimenal C (**2**) HSQC NMR spectrum (800 MHz, $CDCl_3$).

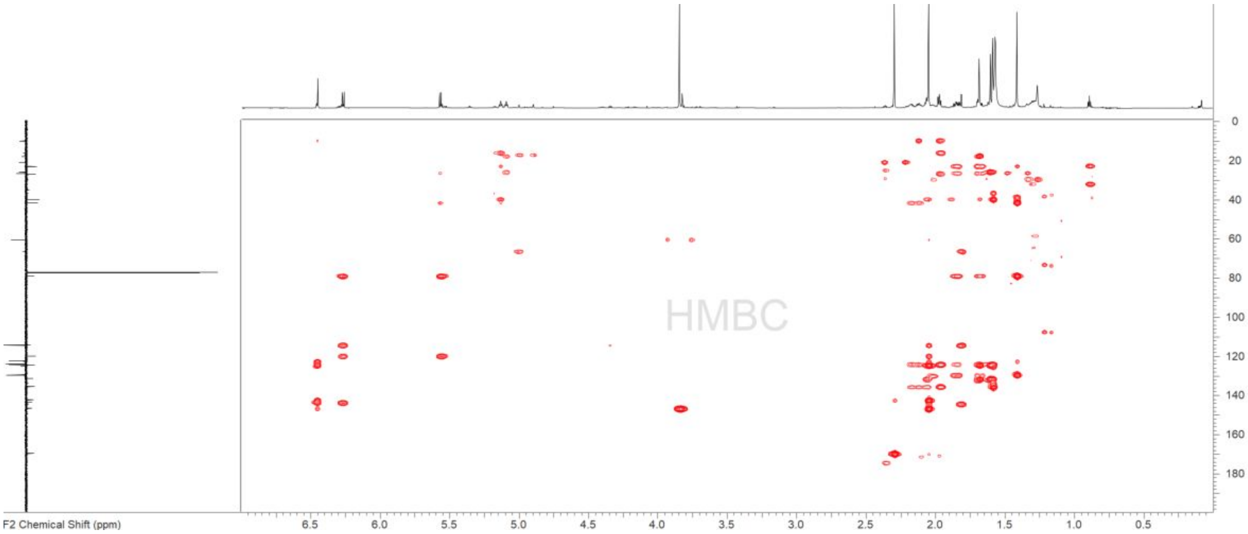

Figure S14: Tuaimenal C (**2**) HMBC NMR spectrum (800 MHz, $CDCl_3$).

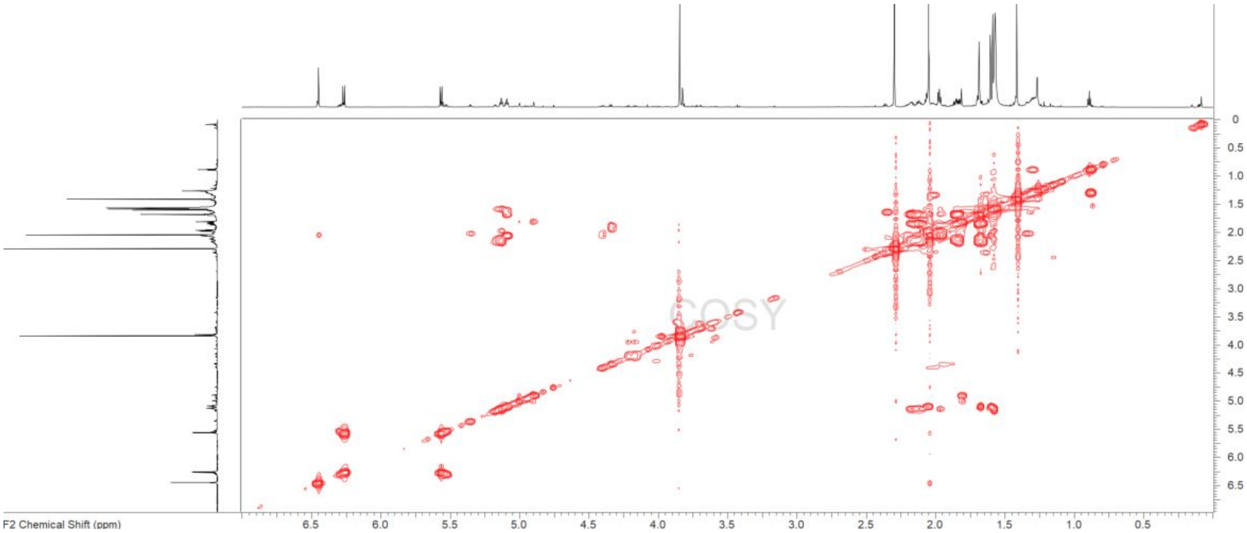

Figure S15: Tuaimenal C (**2**) COSY NMR spectrum (800 MHz, $CDCl_3$).

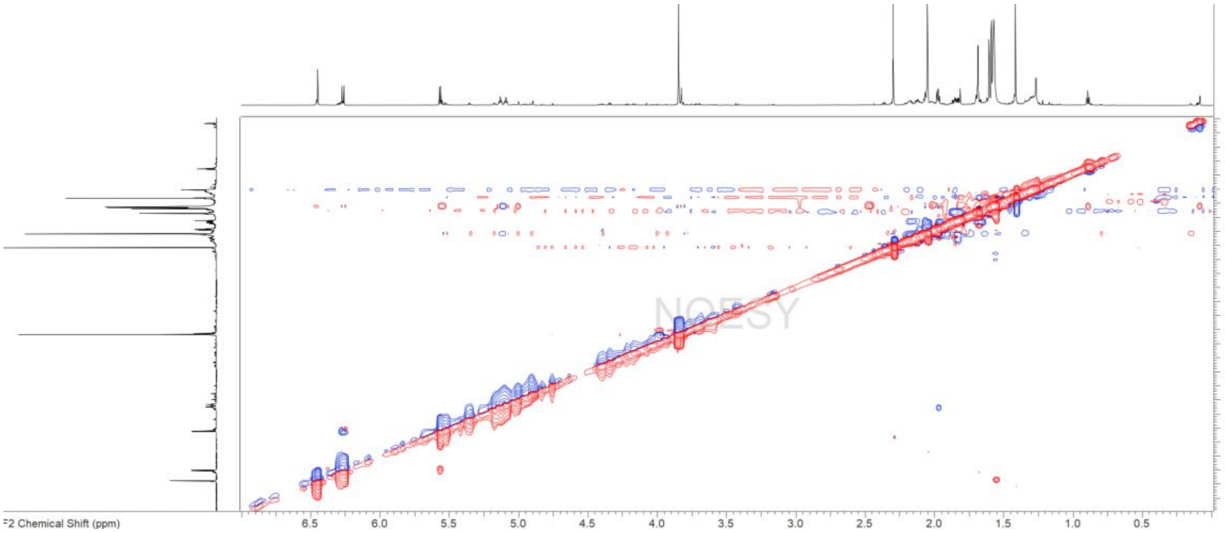

Figure S16: Tuaimenal C (**2**) NOESY NMR spectrum (800 MHz, $CDCl_3$).

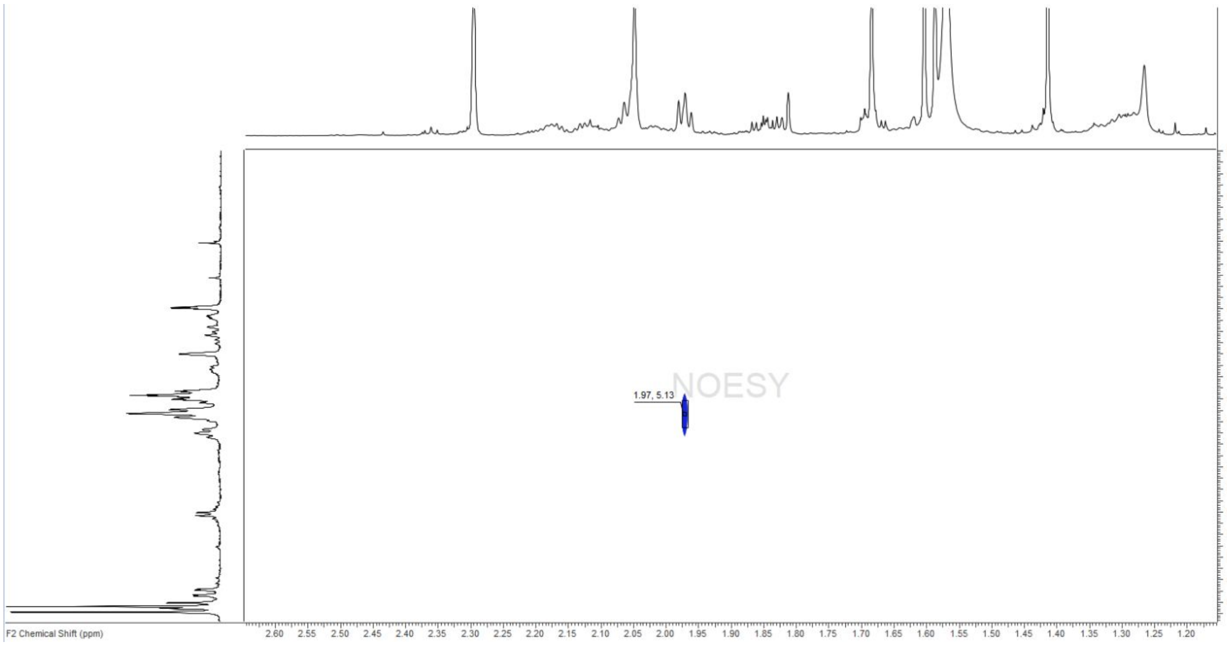

Figure S17: Tuaimenal C (**2**) zoomed NOESY NMR spectrum (800 MHz, $CDCl_3$).

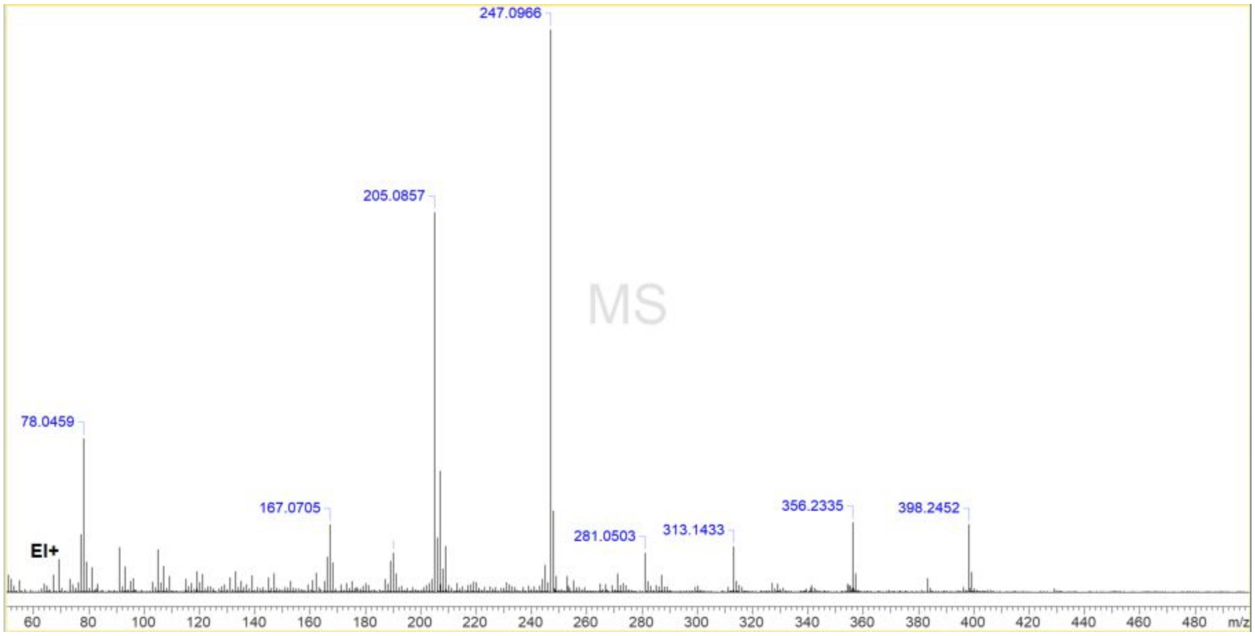

Figure S18: Tuaimenal C (**2**) HREIMS.

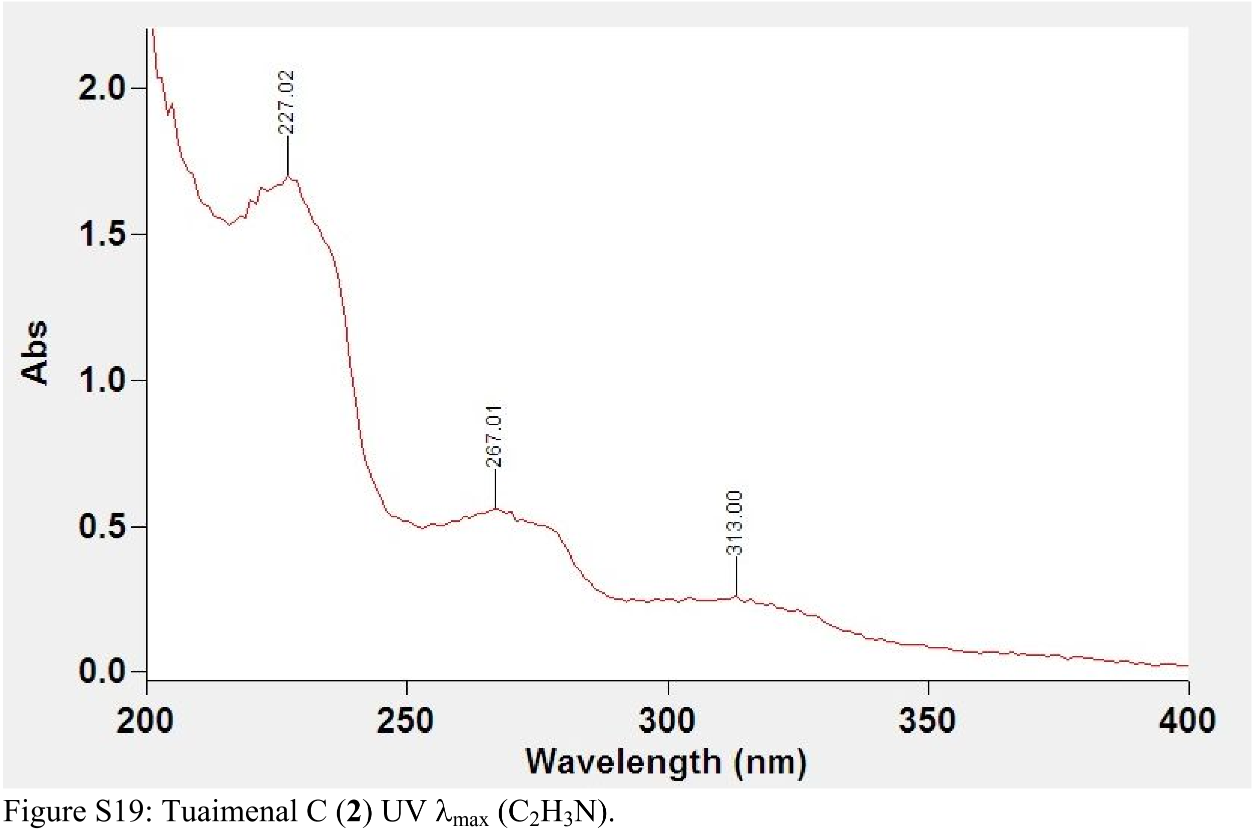

Figure S19: Tuaimenal C (**2**) UV λ_{max} (C_2H_3N).

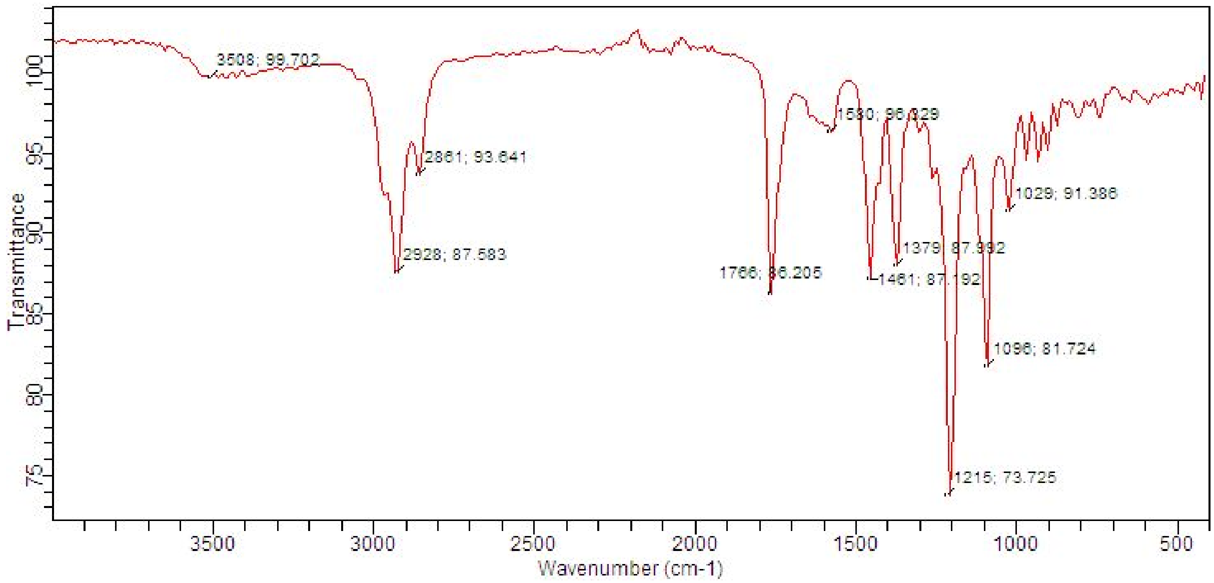

Figure S20: Tuaimenal C (**2**) IR spectrum (thin film).

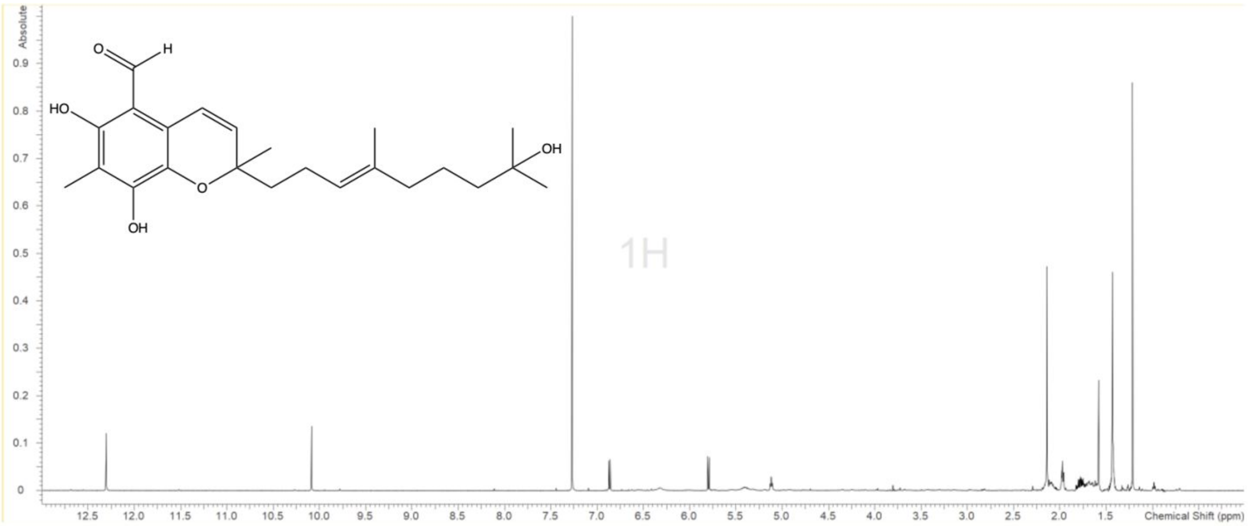

Figure S21: Tuaimenal D (**3**) ^{1}H NMR spectrum (600 MHz, $CDCl_3$).

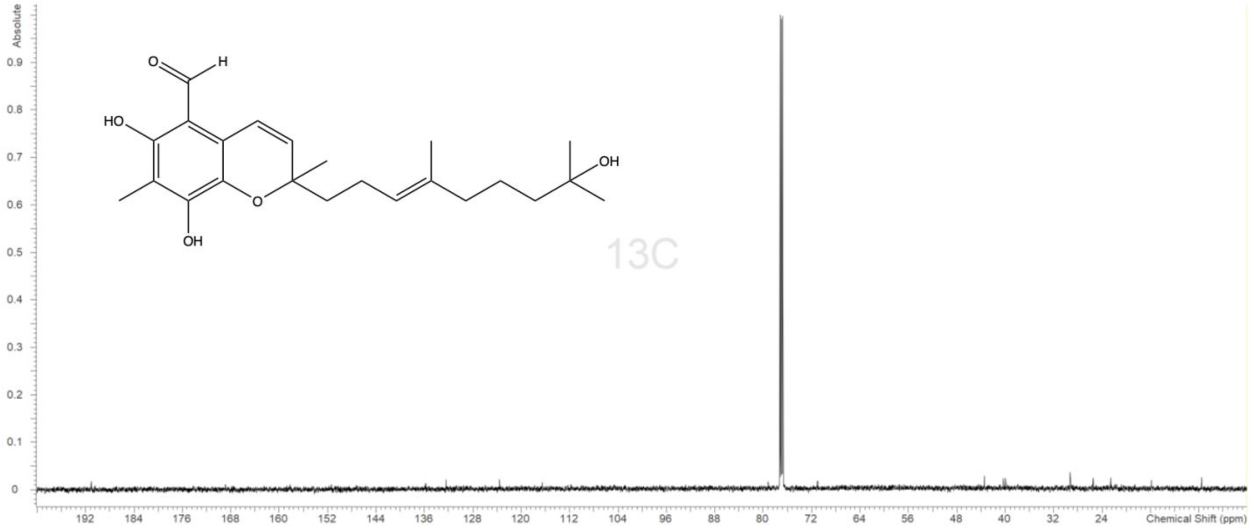

Figure S22: Tuaimenal D (**3**) ^{13}C NMR spectrum (150 MHz, $CDCl_3$).

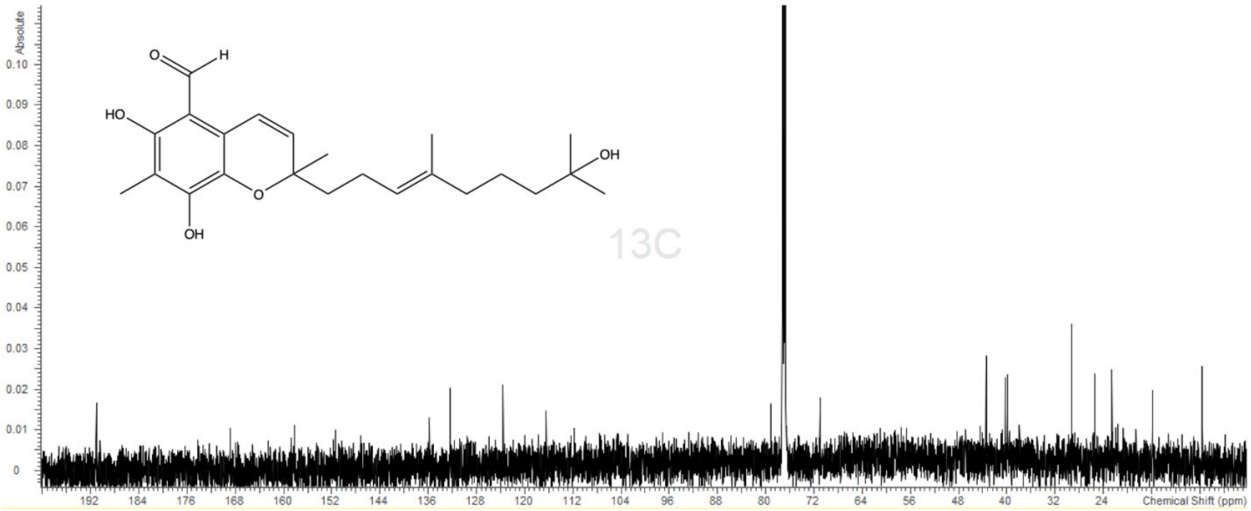

Figure S23: Tuaimenal D (**3**) ^{13}C NMR spectrum zoomed (150 MHz, $CDCl_3$).

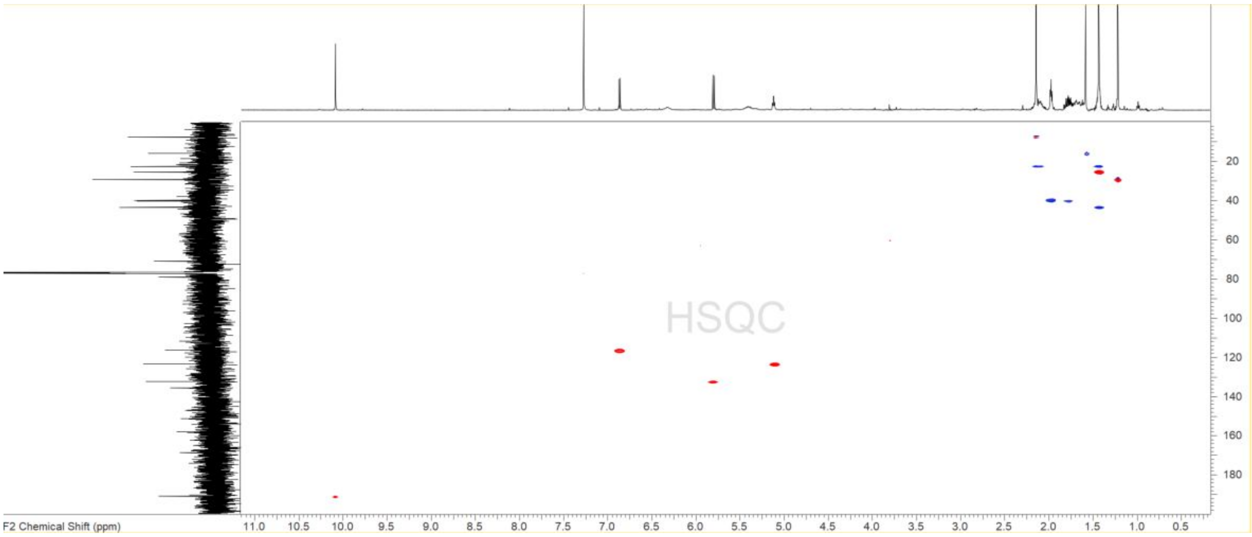

Figure S24: Tuaimenal D (**3**) HSCQ NMR spectrum (500 MHz, $CDCl_3$).

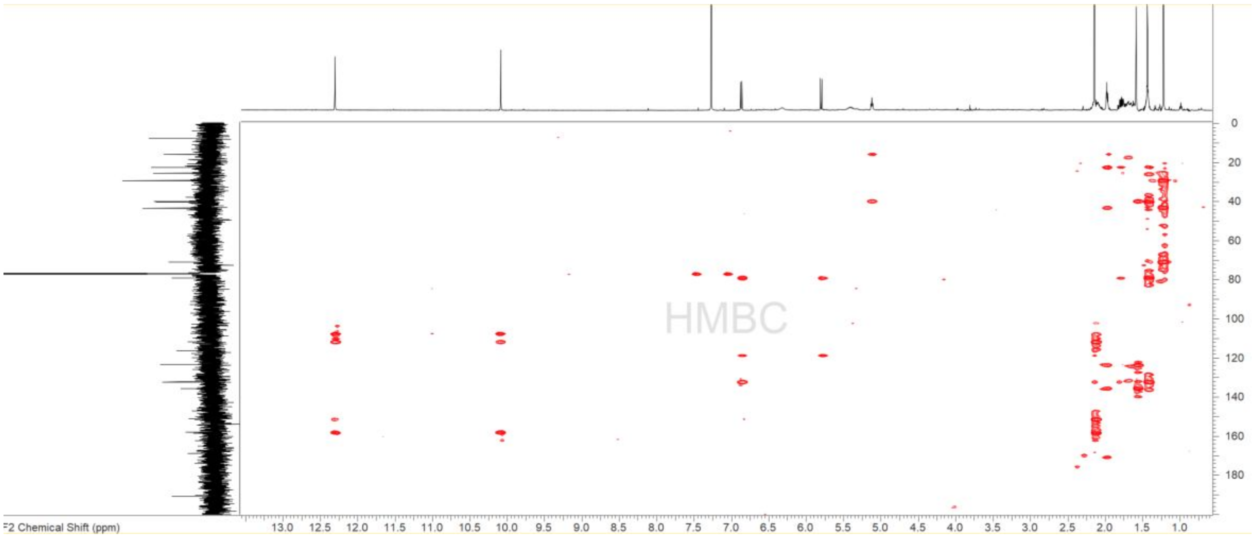

Figure S25: Tuaimenal D (**3**) HMBC NMR spectrum (500 MHz, $CDCl_3$).

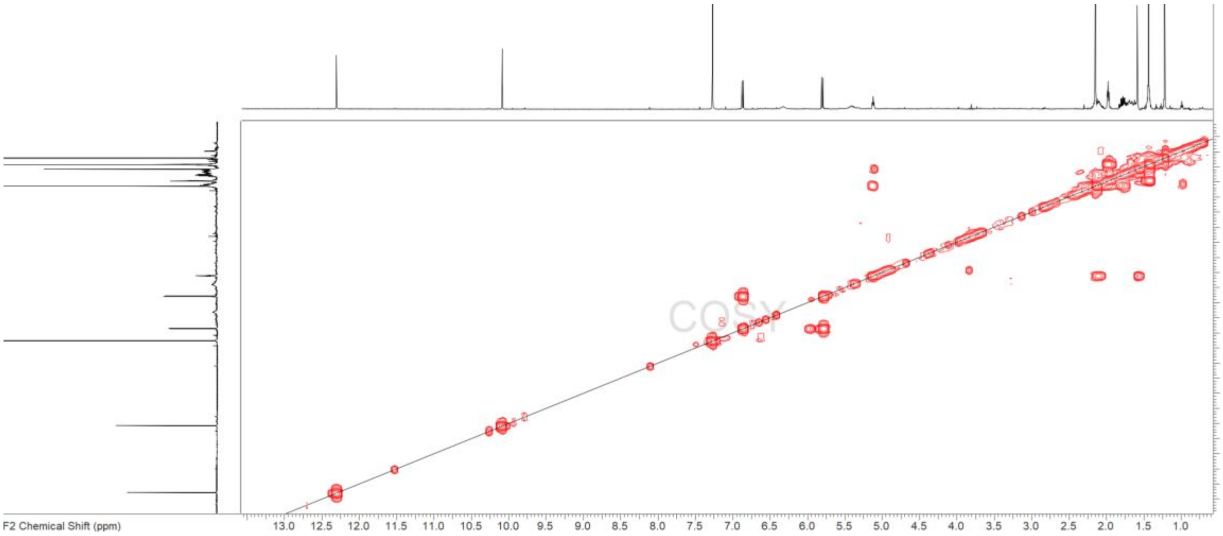

Figure S26: Tuaimenal D (**3**) COSY NMR spectrum (500 MHz, $CDCl_3$).

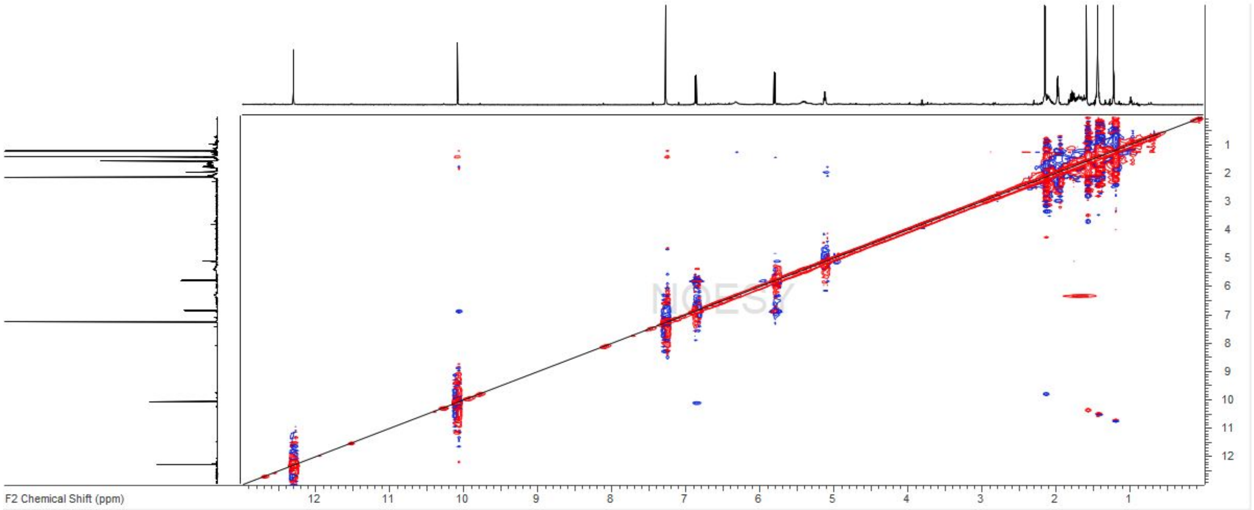

Figure S27: Tuaimenal D (**3**) NOESY NMR spectrum (500 MHz, $CDCl_3$).

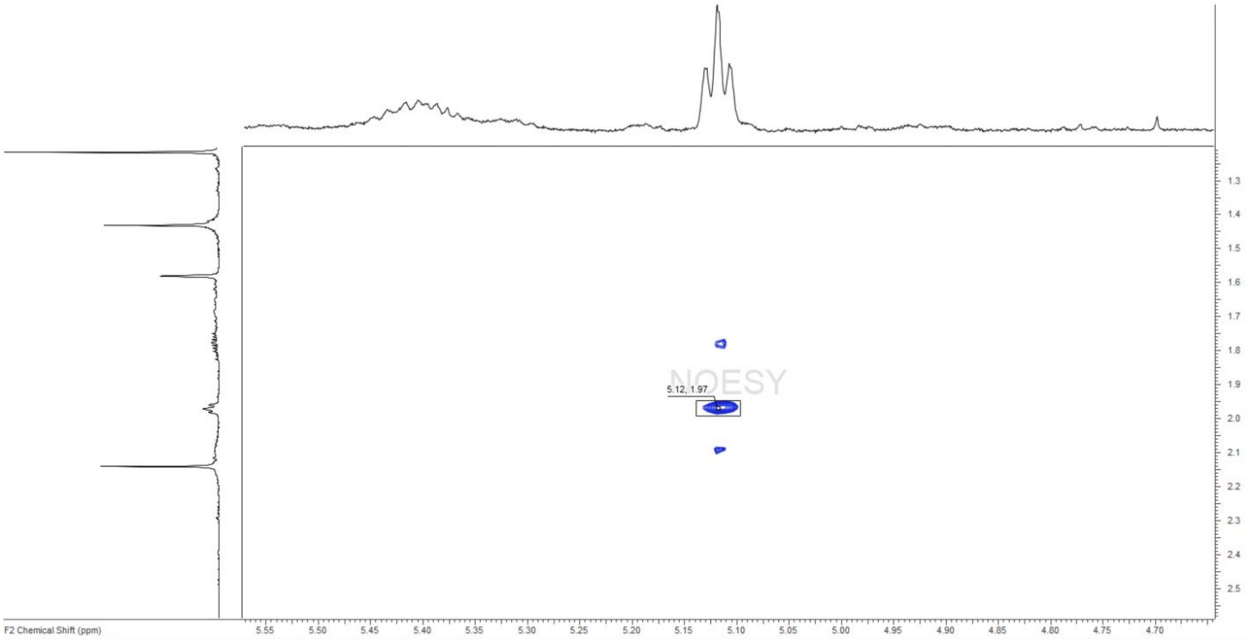

Figure S28: Tuaimenal D (**3**) zoomed NOESY NMR spectrum (500 MHz, $CDCl_3$).

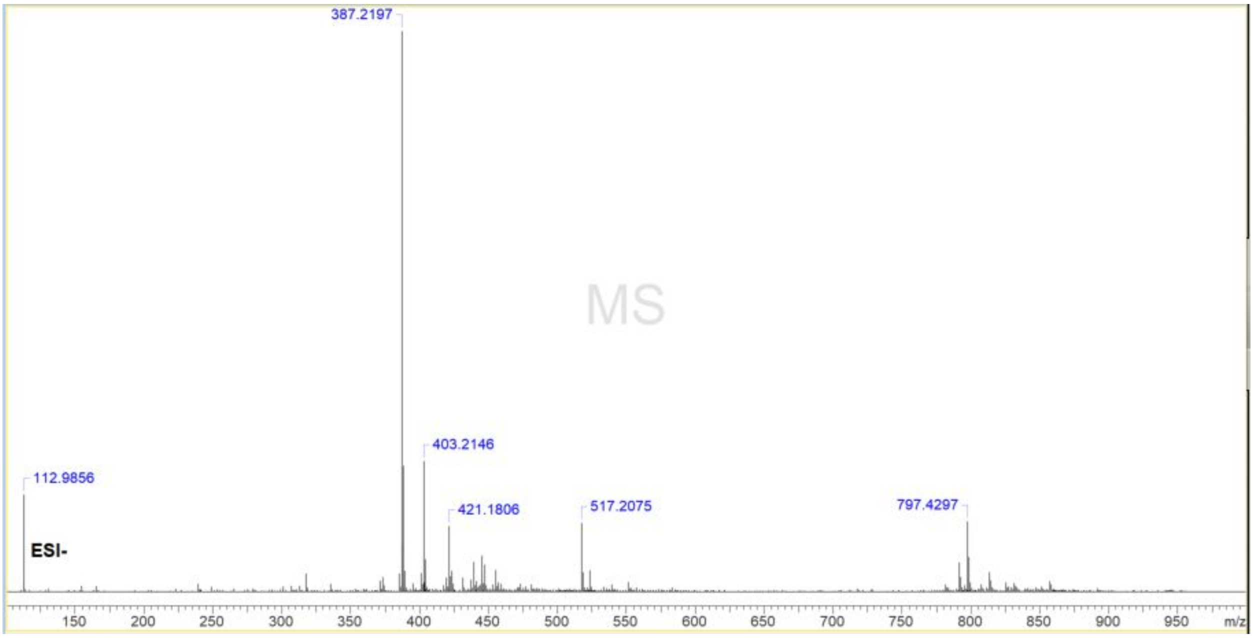

Figure S29: Tuaimenal D (**3**) HRESIMS (neg).

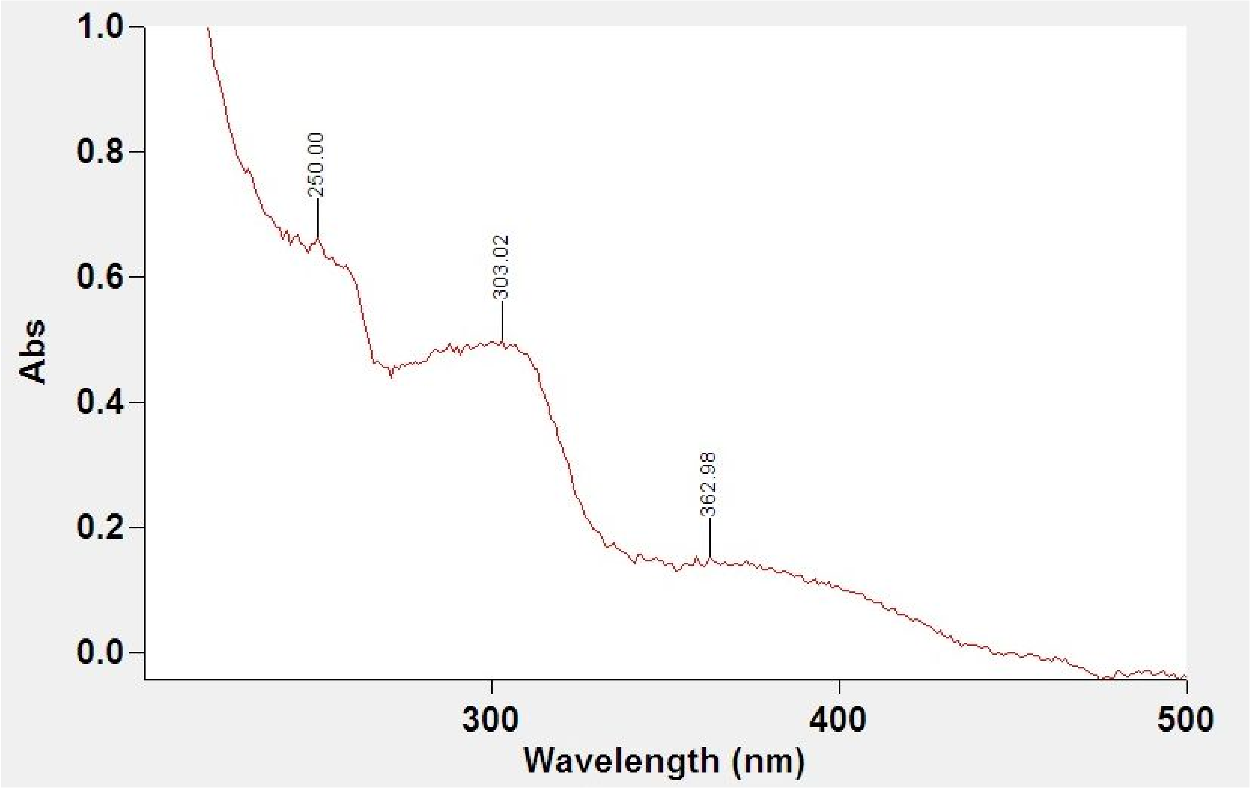

Figure S30: Tuaimenal D (**3**) UV λ_{max} (C_2H_3N).

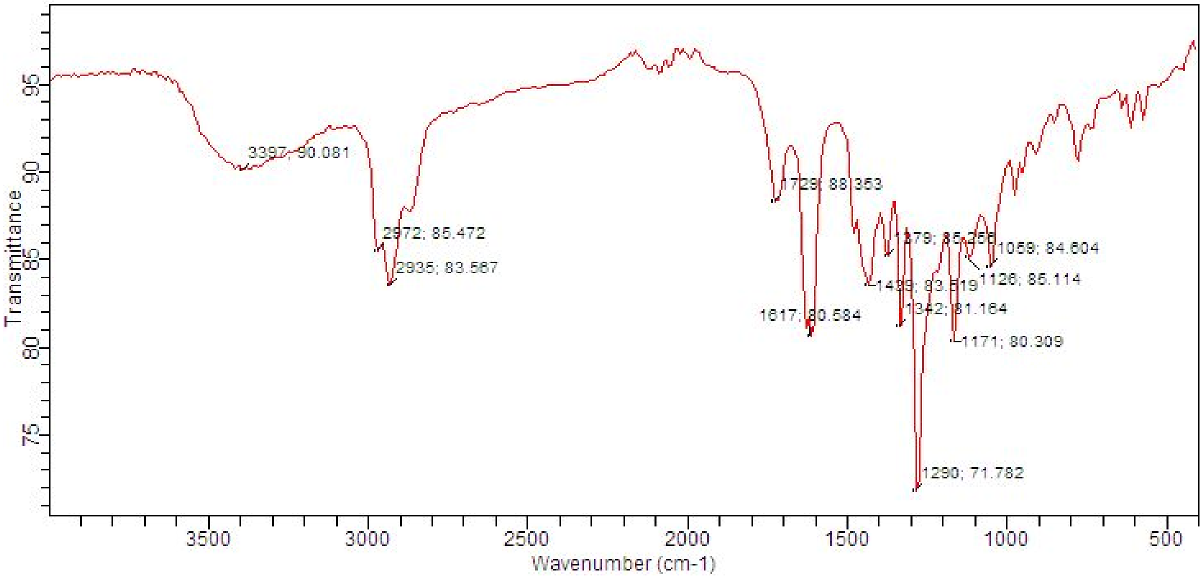

Figure S31: Tuaimenal D (**3**) IR spectrum (thin film).

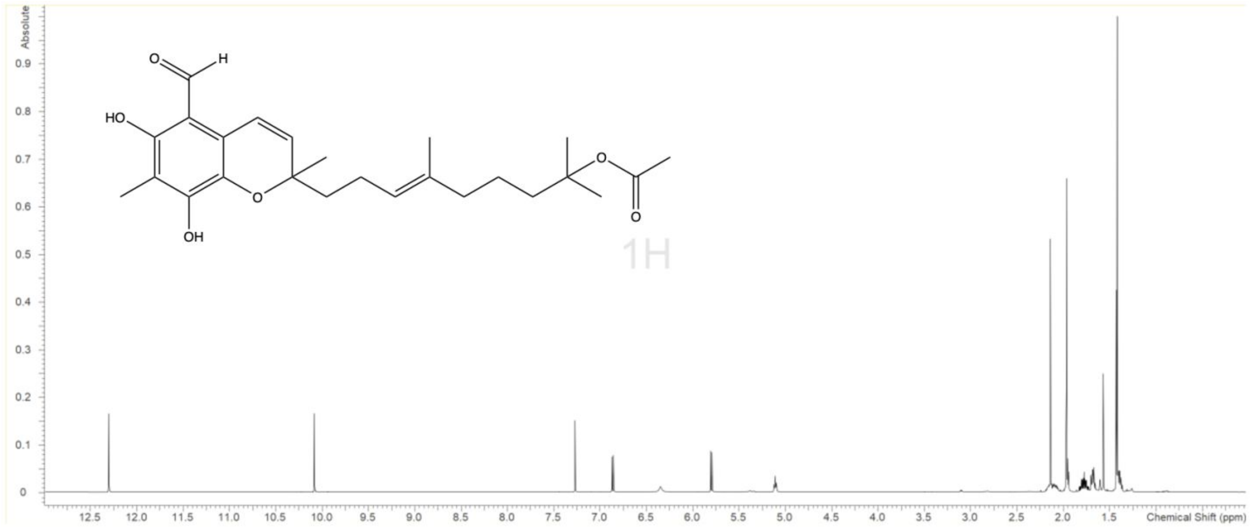

Figure S32: Tuaimenal E (**4**) ^{1}H NMR spectrum (600 MHz, $CDCl_3$).

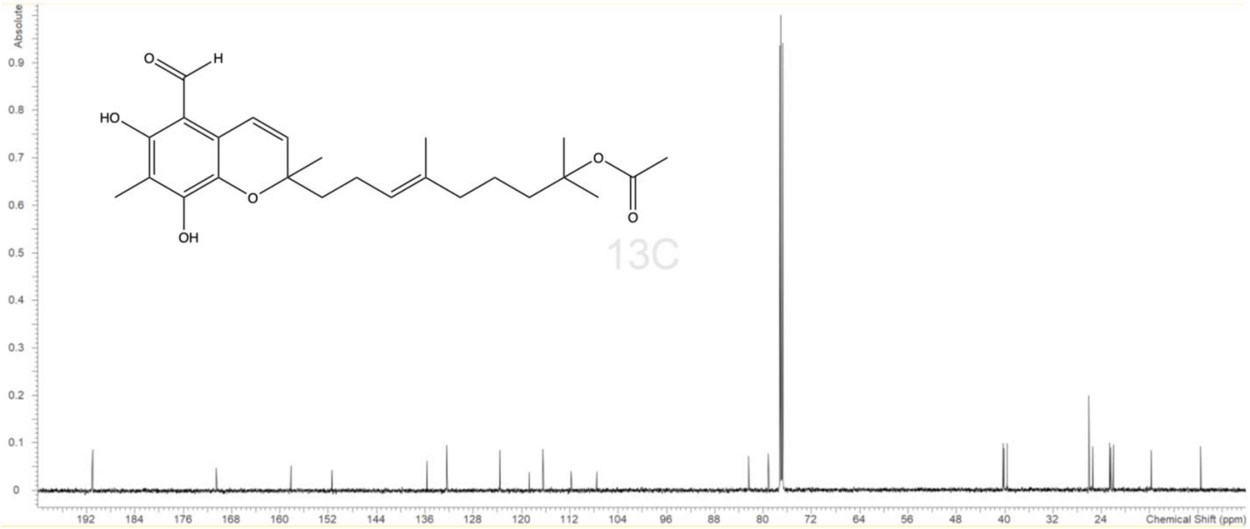

Figure S33: Tuaimenal E (**4**) ^{13}C NMR spectrum (150 MHz, $CDCl_3$).

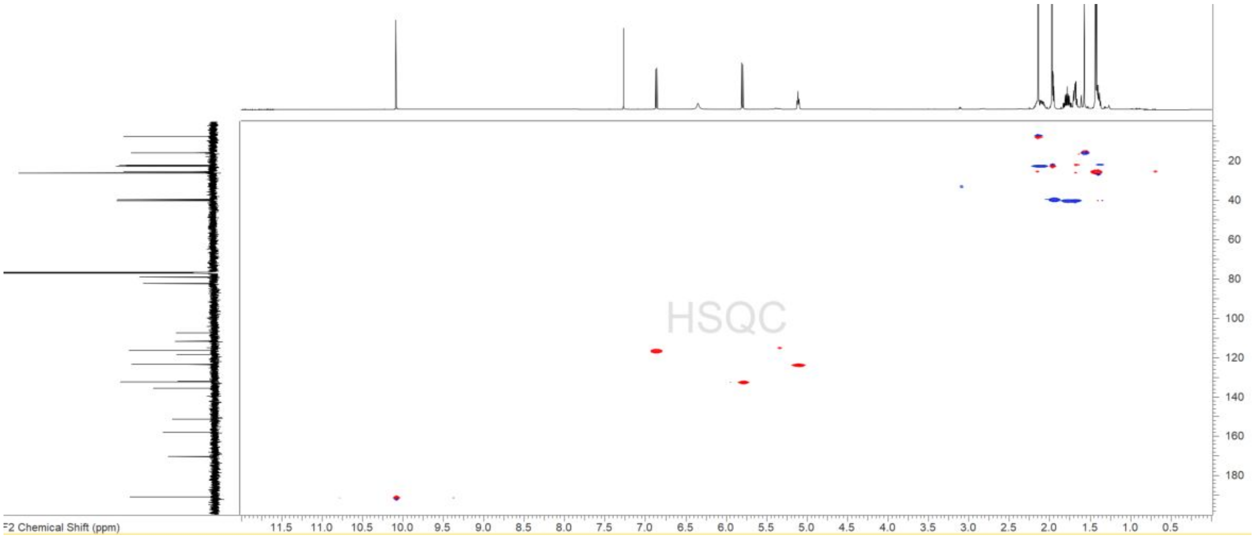

Figure S34: Tuaimenal E (**4**) HSCQ NMR spectrum (500 MHz, $CDCl_3$).

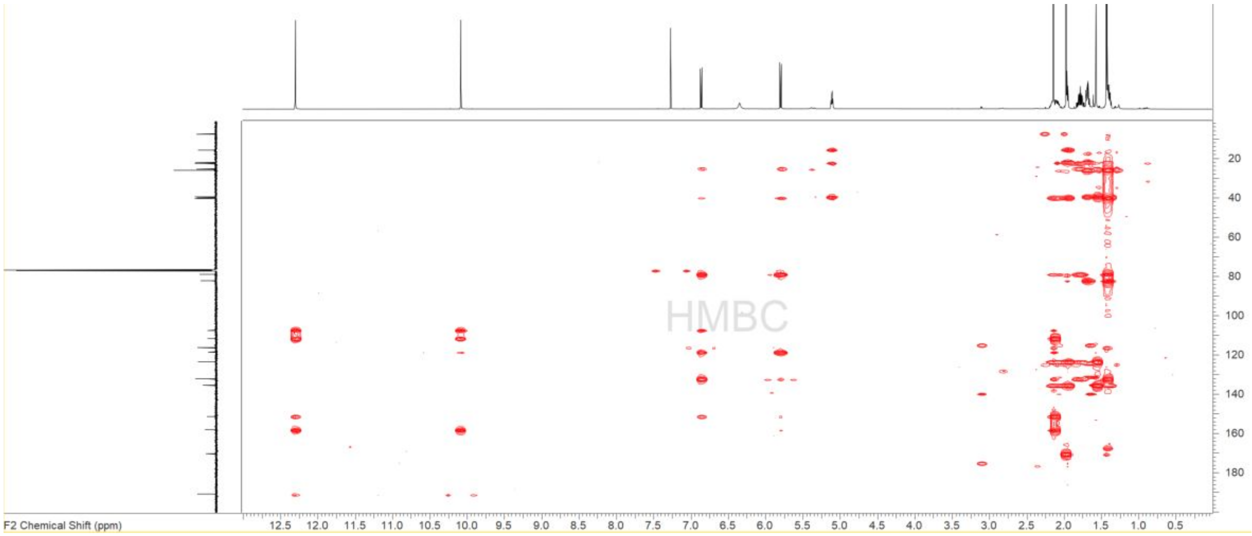

Figure S35: Tuaimenal E (**4**) HMBC NMR spectrum (500 MHz, $CDCl_3$).

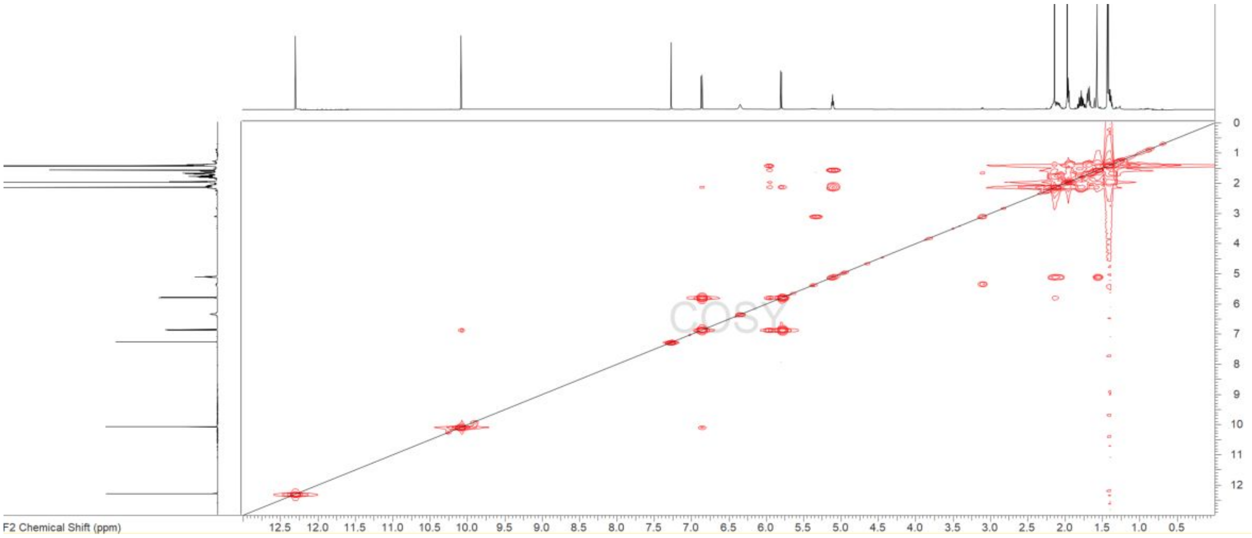

Figure S36: Tuaimenal E (**4**) COSY NMR spectrum (500 MHz, $CDCl_3$).

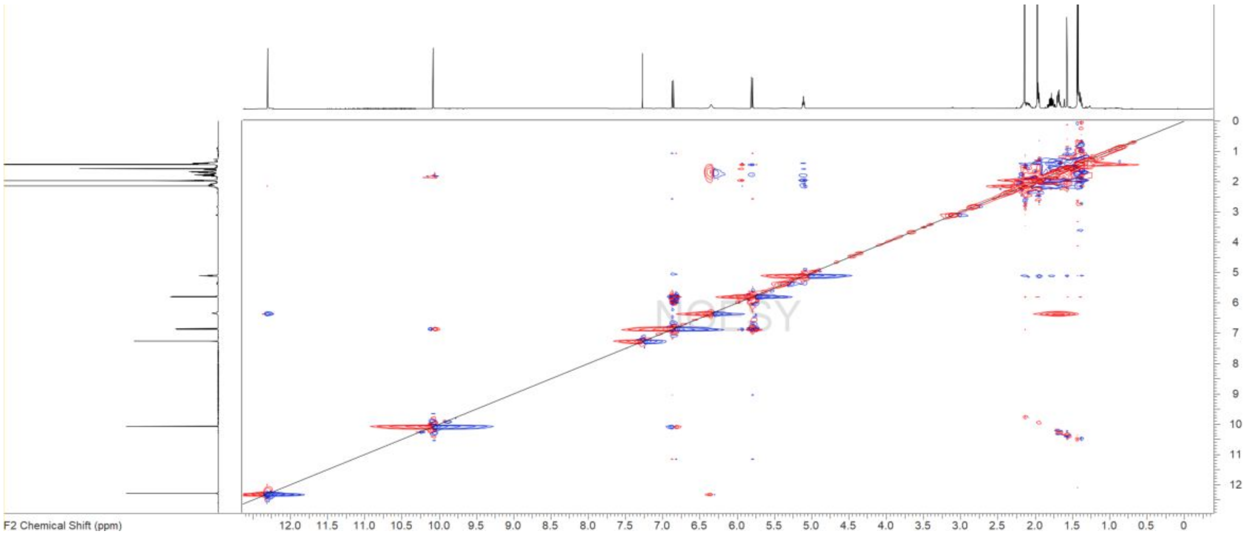

Figure S37: Tuaimenal E (**4**) NOESY NMR spectrum (500 MHz, $CDCl_3$).

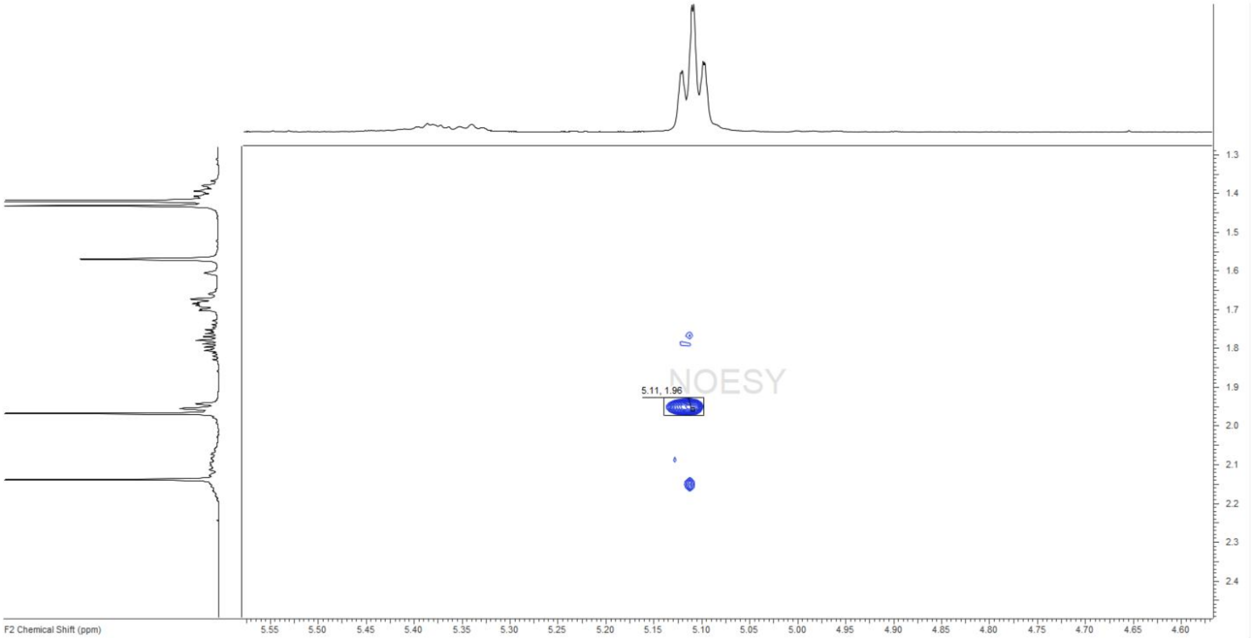

Figure S38: Tuaimenal E (**4**) zoomed NOESY NMR spectrum (500 MHz, $CDCl_3$).

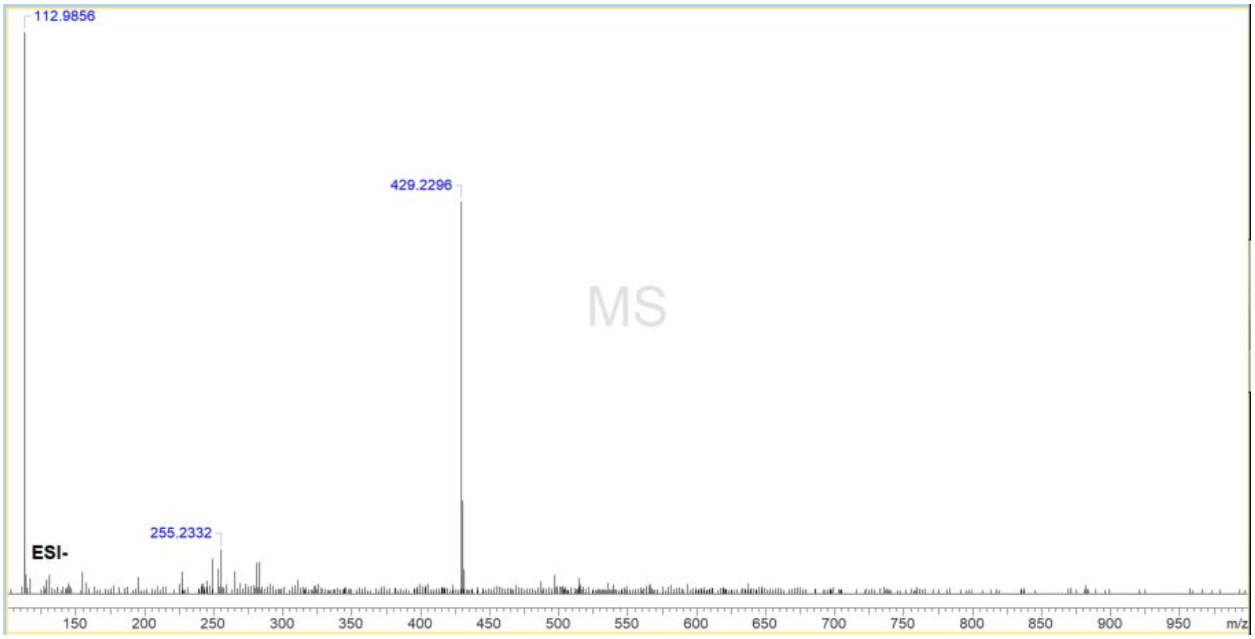

Figure S39: Tuaimenal E (**4**) HRESIMS (neg).

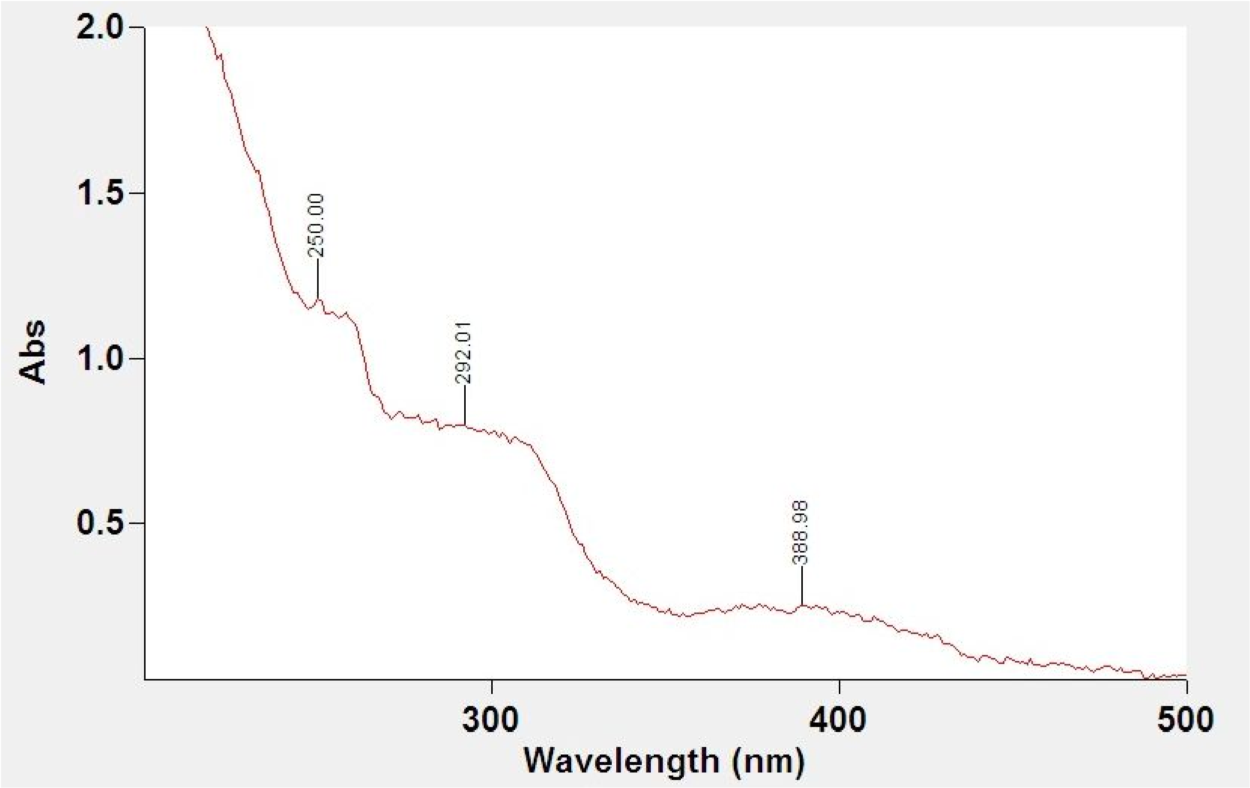

Figure S40: Tuaimenal E (**4**) UV λ_{max} (C_2H_3N).

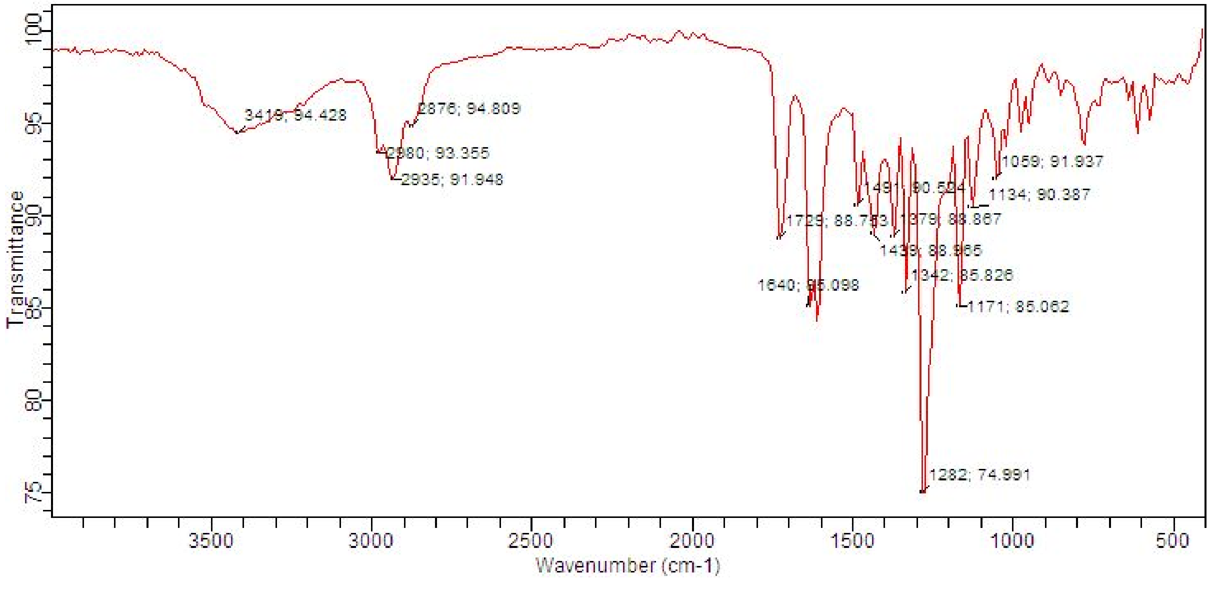

Figure S41: Tuaimenal E (**4**) IR spectrum (thin film).

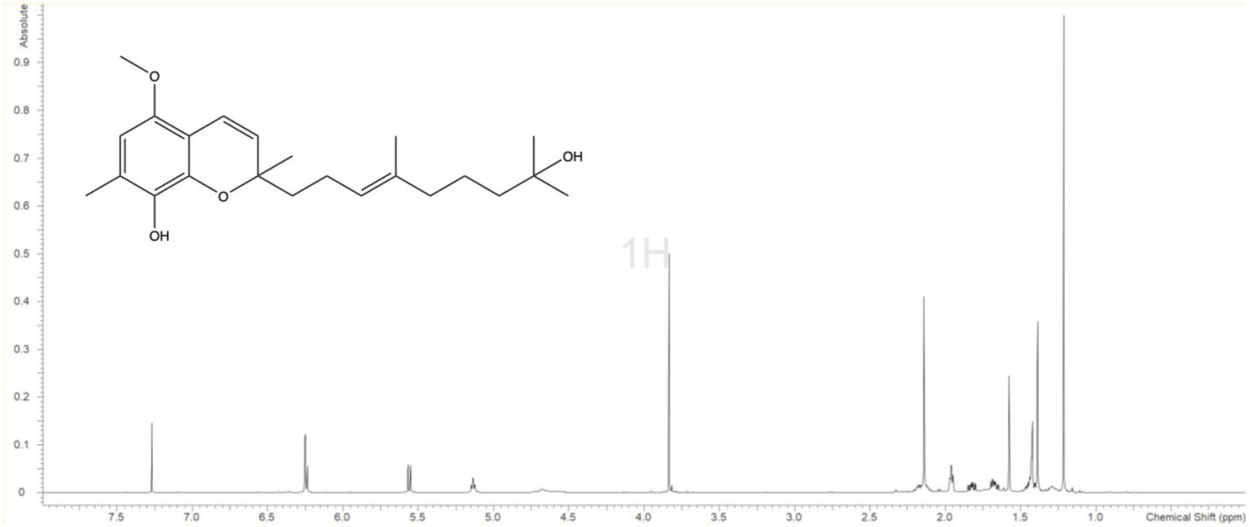

Figure S42: Tuaimenal F (**5**) ^{1}H NMR spectrum (600 MHz, $CDCl_3$).

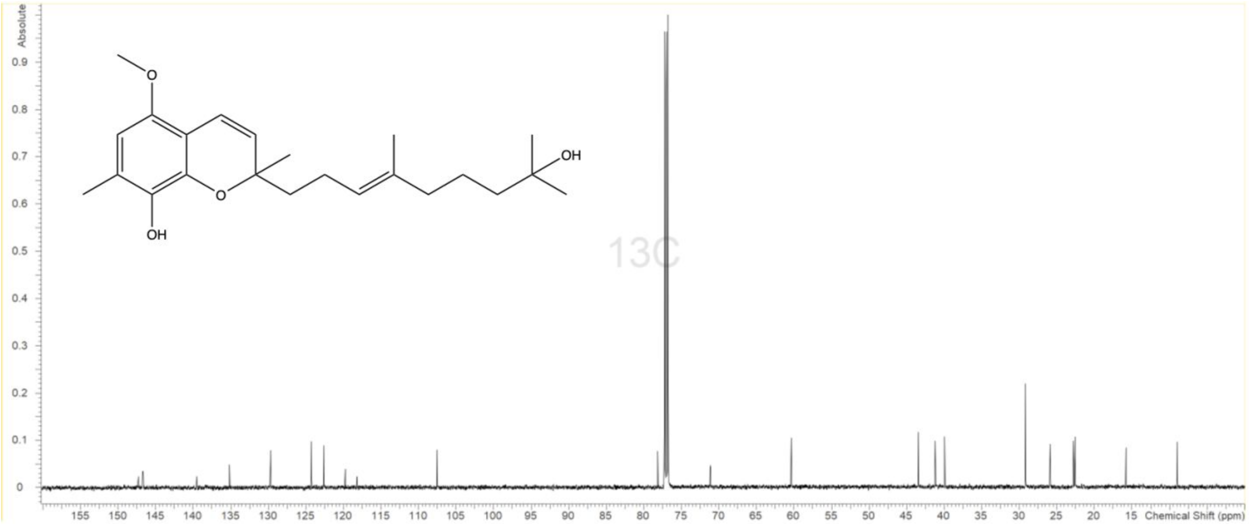

Figure S43: Tuaimenal F (**5**) ^{13}C NMR spectrum (150 MHz, $CDCl_3$).

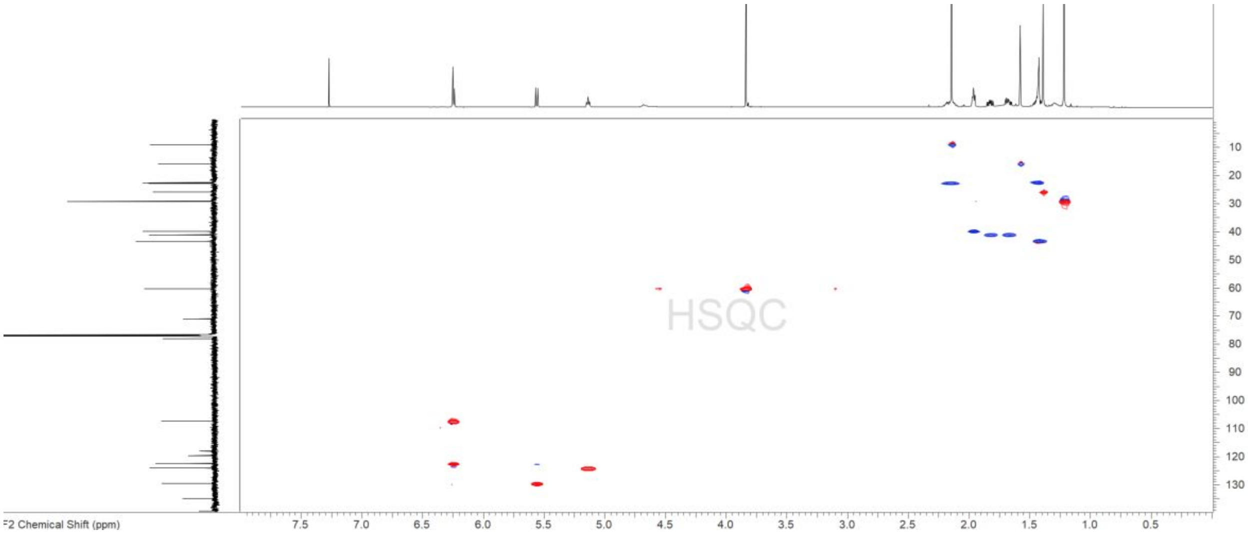

Figure S44: Tuaimenal F (**5**) HSCQ NMR spectrum (500 MHz, $CDCl_3$).

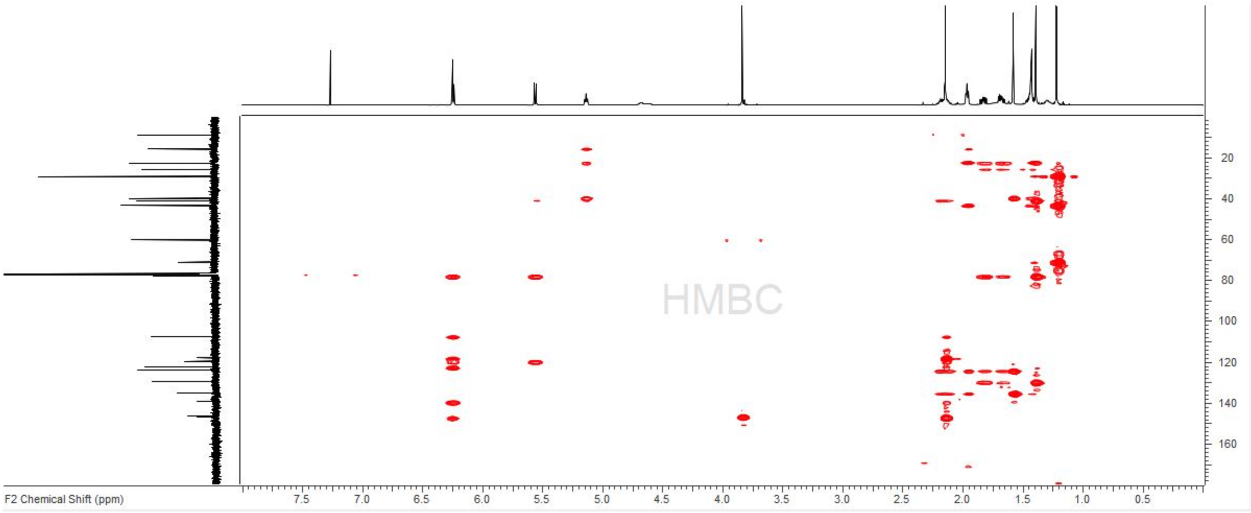

Figure S45: Tuaimenal F (**5**) HMBC NMR spectrum (500 MHz, $CDCl_3$).

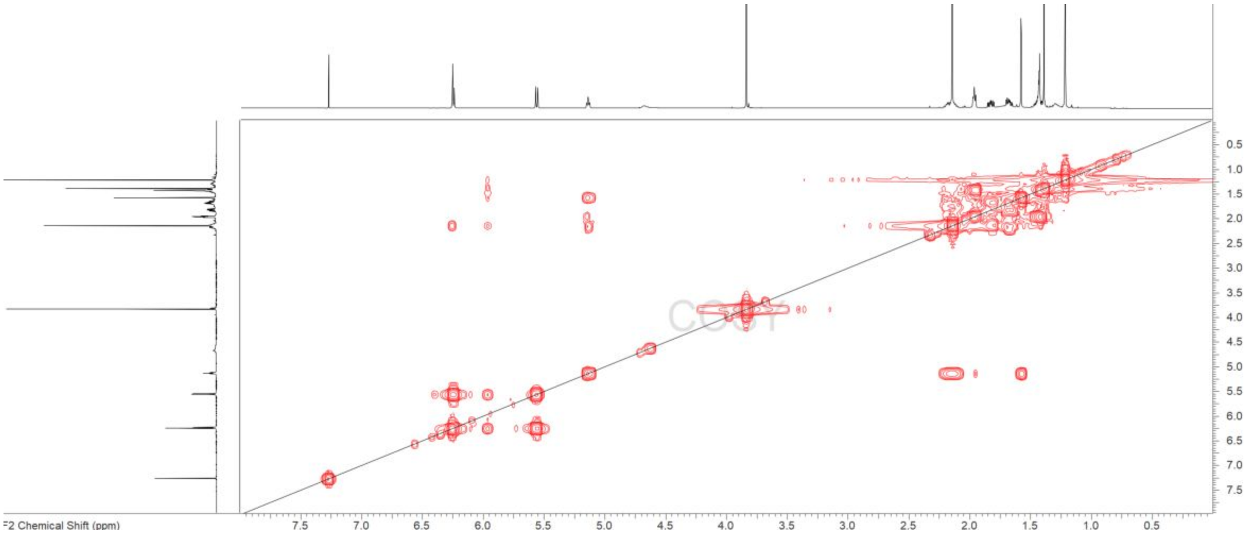

Figure S46: Tuaimenal F (**5**) COSY NMR spectrum (500 MHz, $CDCl_3$).

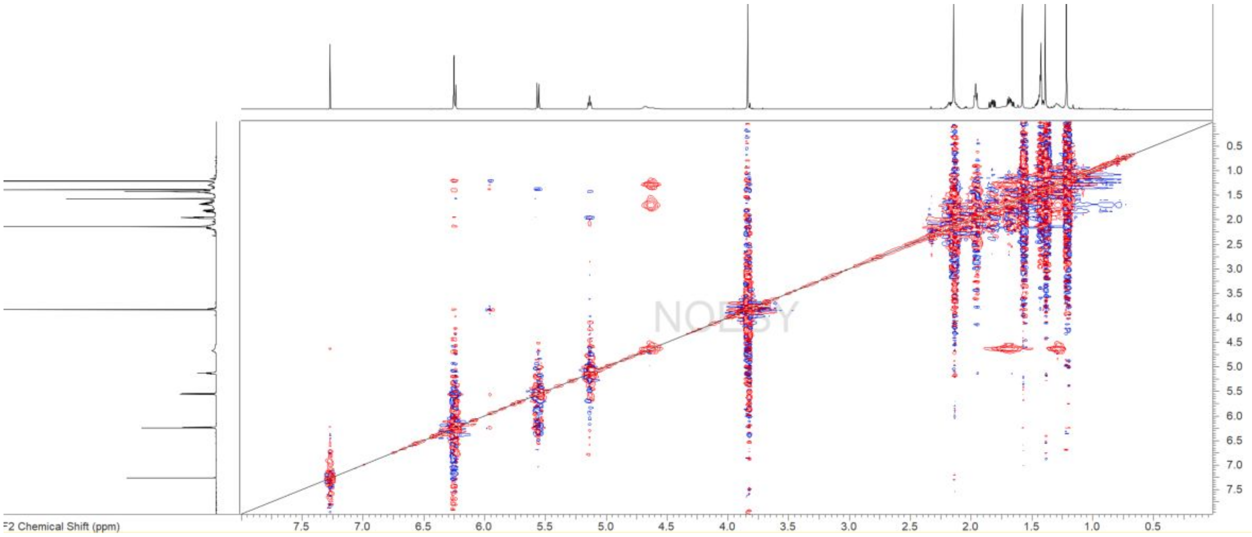

Figure S47: Tuaimenal F (**5**) NOESY NMR spectrum (500 MHz, $CDCl_3$).

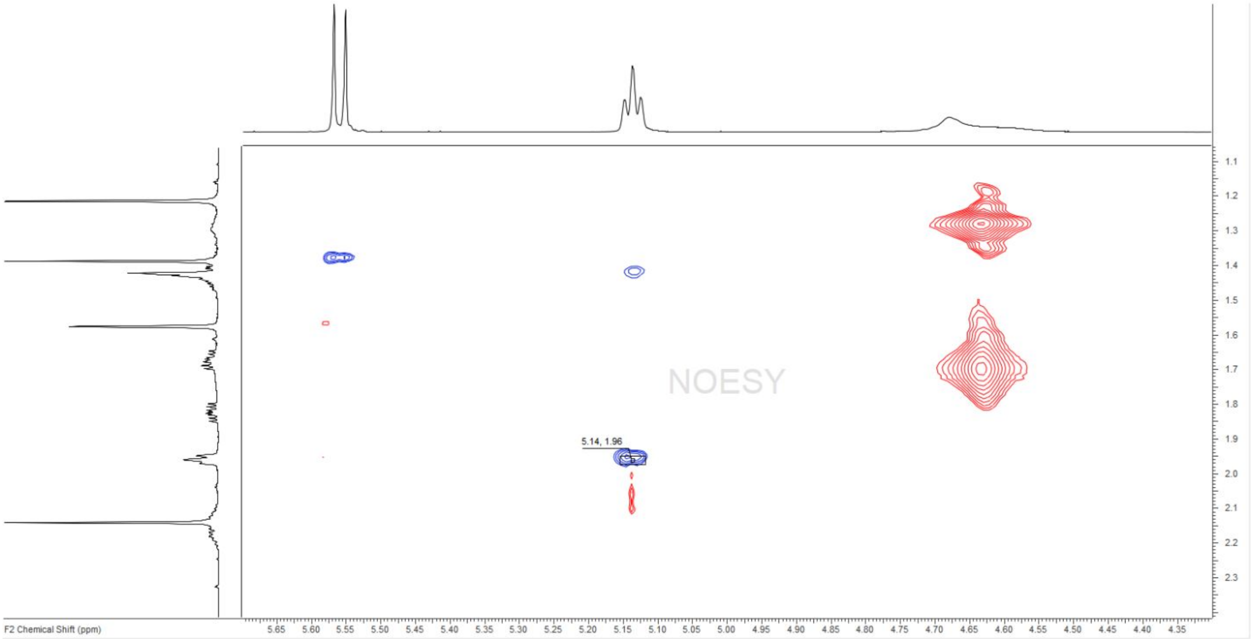

Figure S48: Tuaimenal F (**5**) zoomed NOESY NMR spectrum (500 MHz, $CDCl_3$).

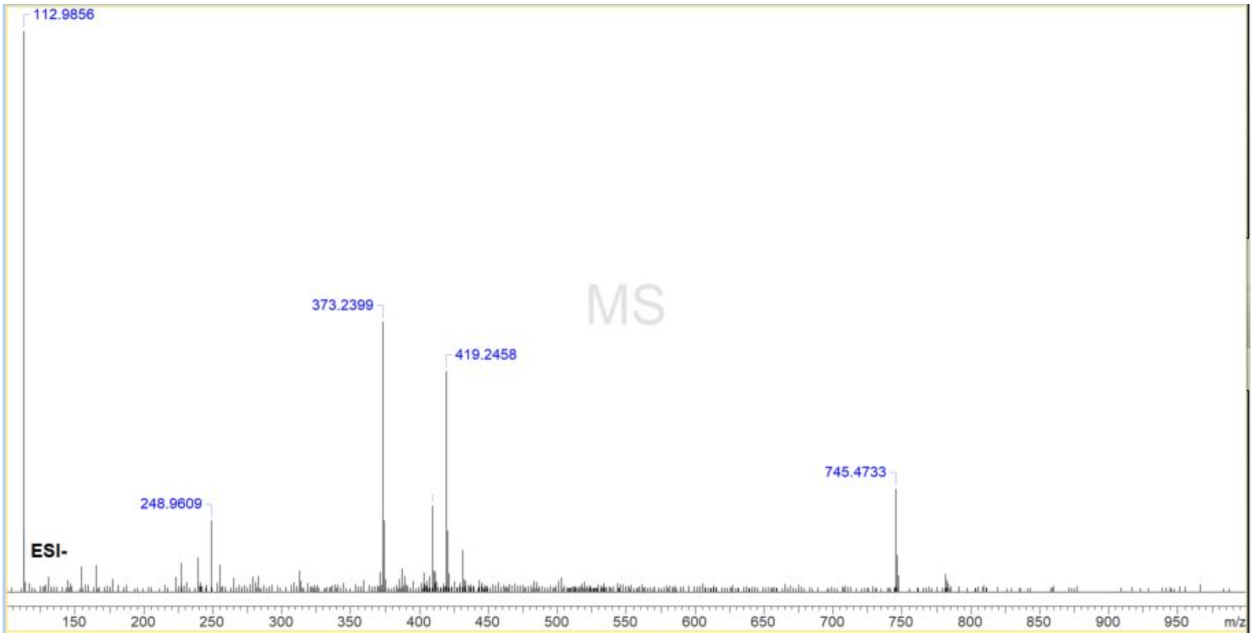

Figure S49: Tuaimenal F (**5**) HRESIMS (neg).

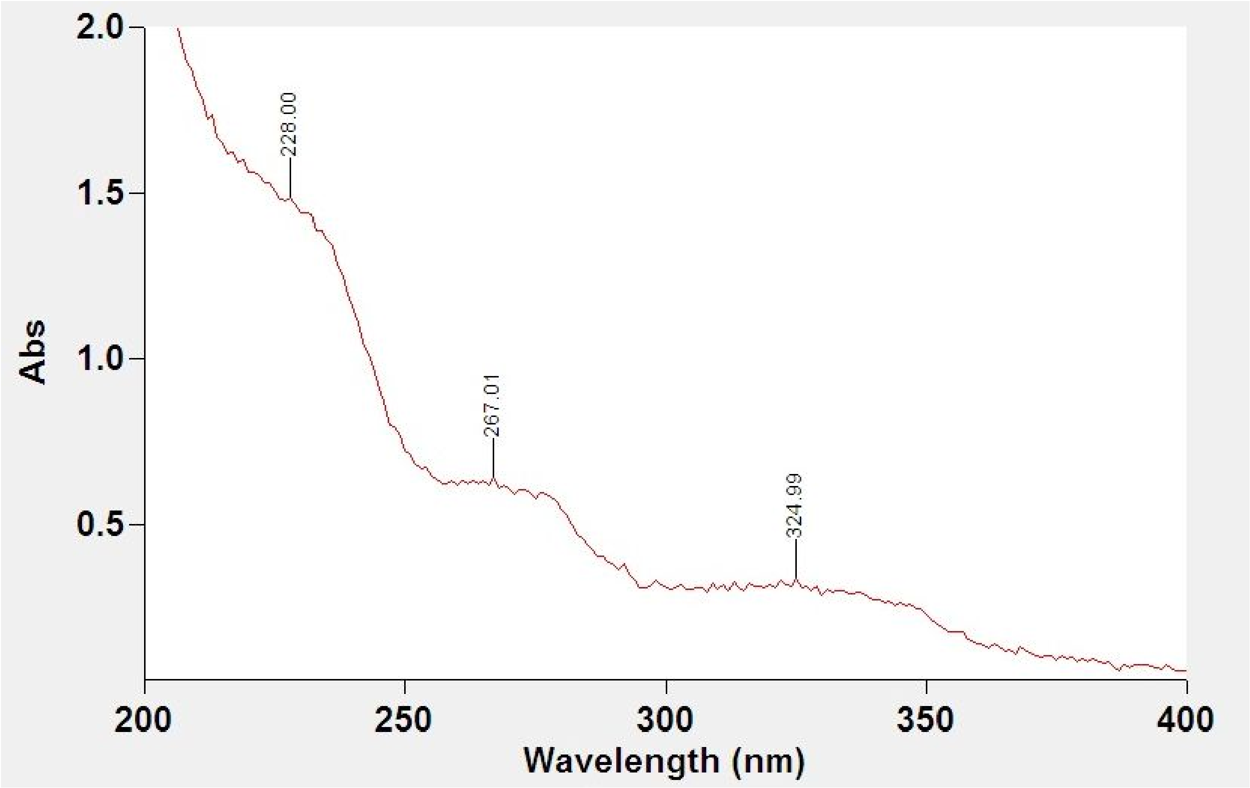

Figure S50: Tuaimenal F (**5**) UV λ_{max} (C_2H_3N).

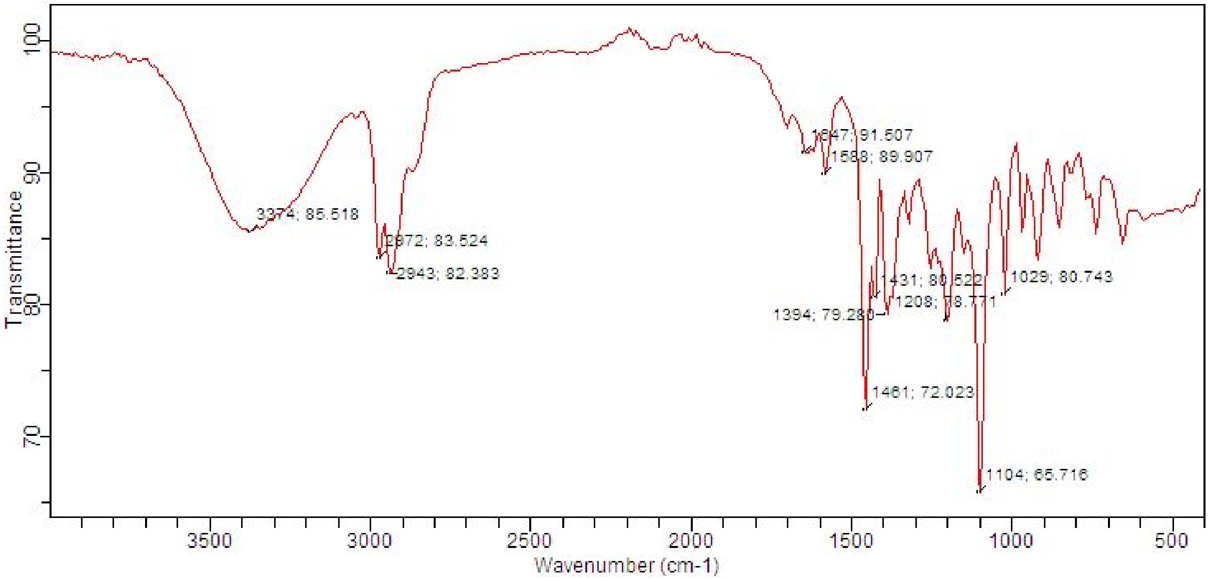

Figure S51: Tuaimenal F (**5**) IR spectrum (thin film).

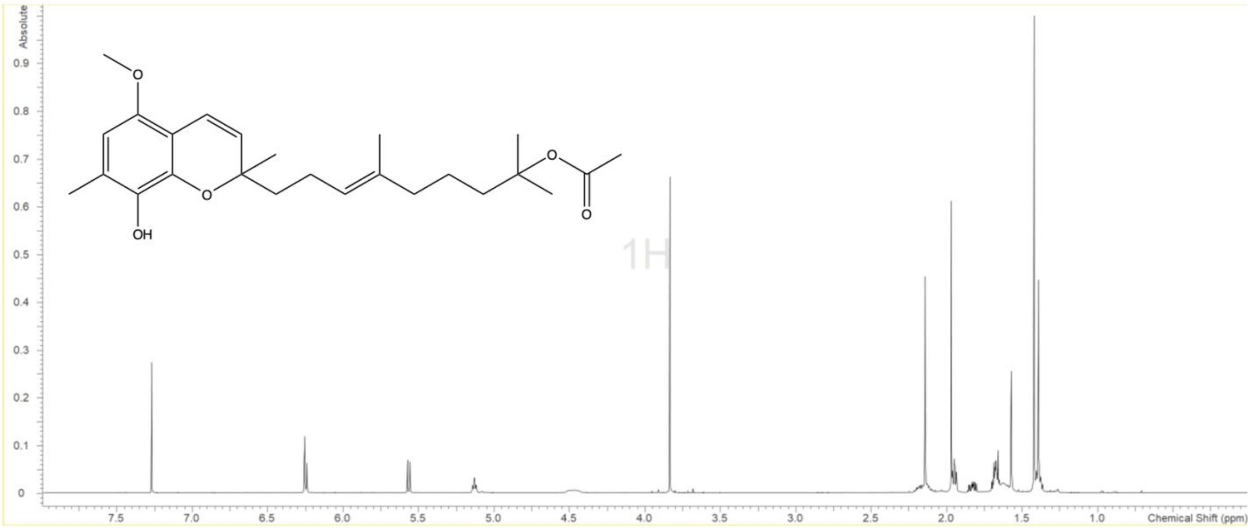

Figure S52: Tuaimenal G (**6**) ^{1}H NMR spectrum (600 MHz, $CDCl_3$).

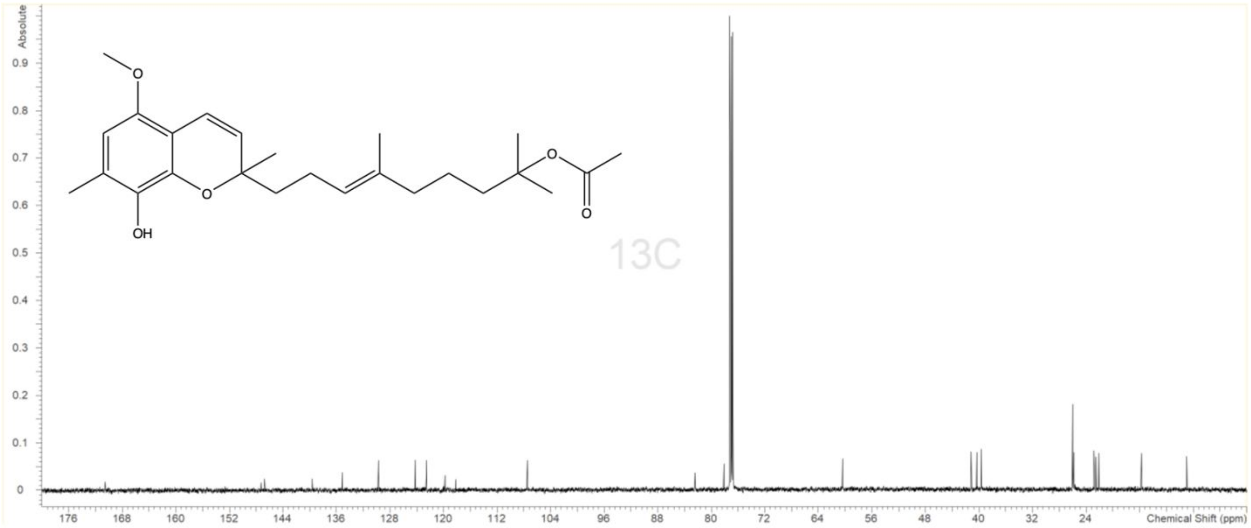

Figure S53: Tuaimenal G (**6**) ^{13}C NMR spectrum (150 MHz, $CDCl_3$).

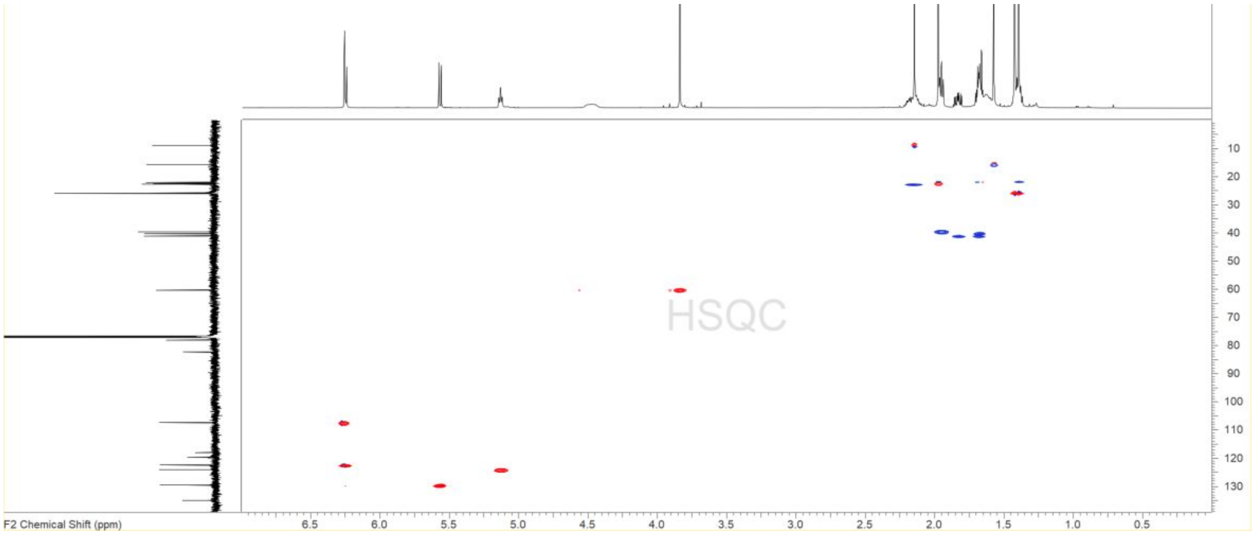

Figure S54: Tuaimenal G (**6**) HSCQ NMR spectrum (500 MHz, $CDCl_3$).

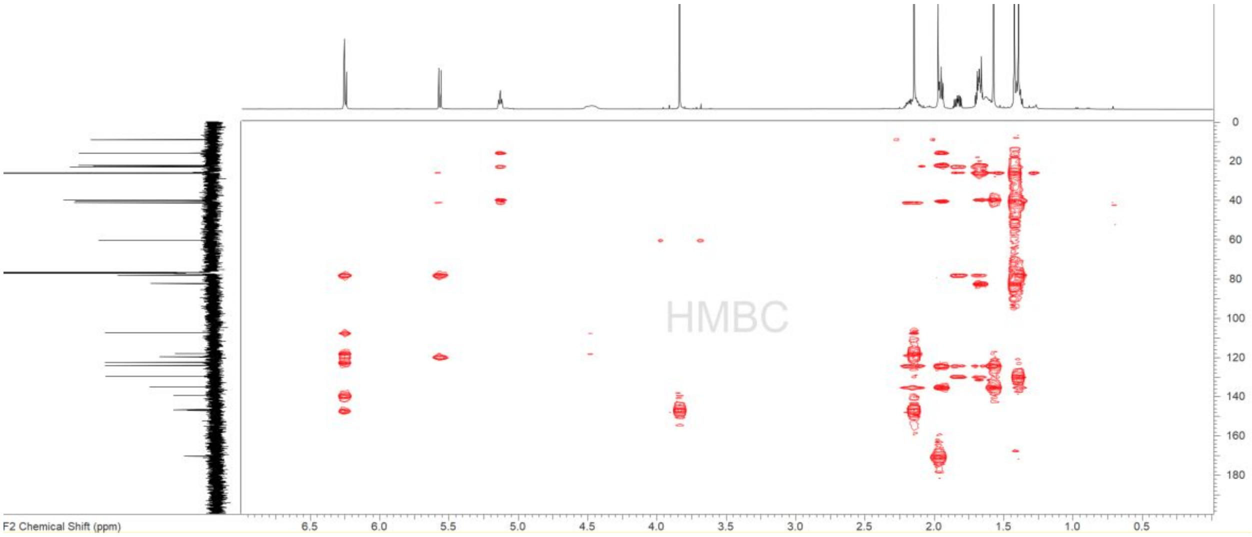

Figure S55: Tuaimenal G (**6**) HMBC NMR spectrum (500 MHz, $CDCl_3$).

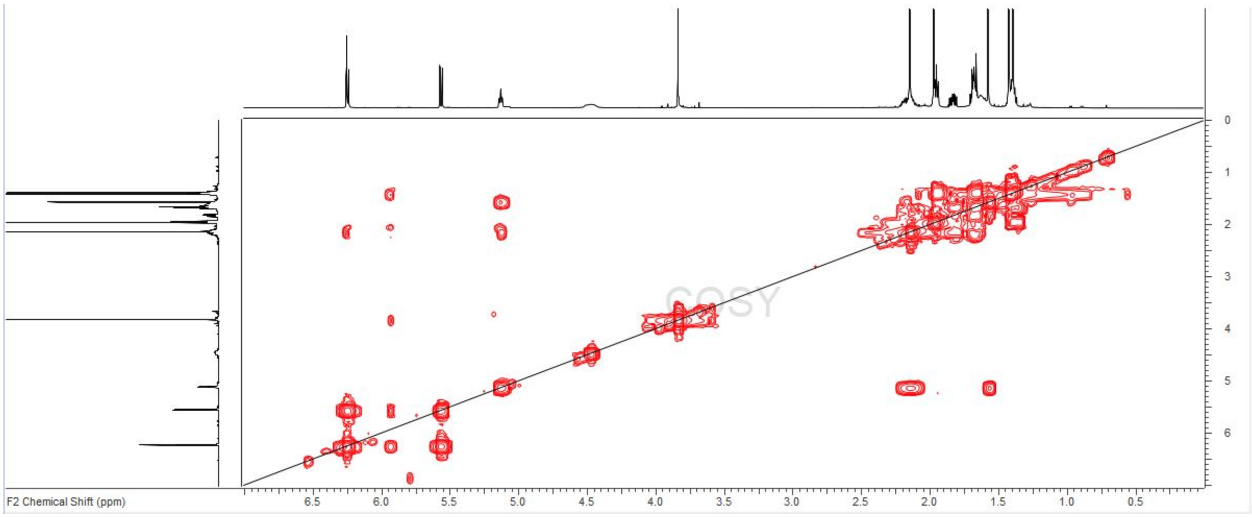

Figure S56: Tuaimenal G (**6**) COSY NMR spectrum (500 MHz, $CDCl_3$).

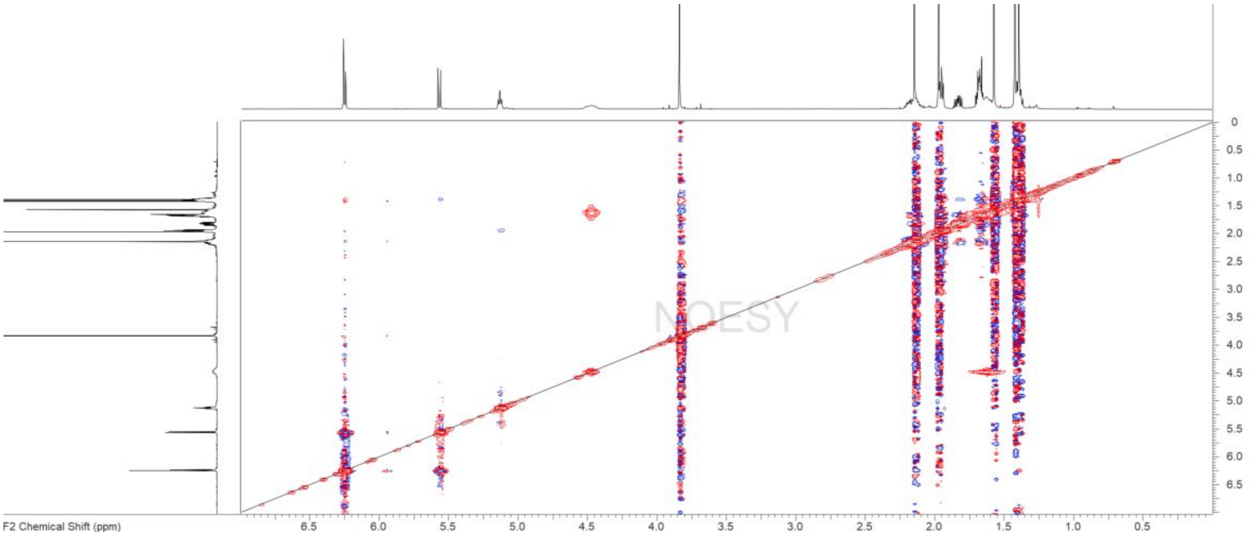

Figure S57: Tuaimenal G (**6**) NOESY NMR spectrum (500 MHz, $CDCl_3$).

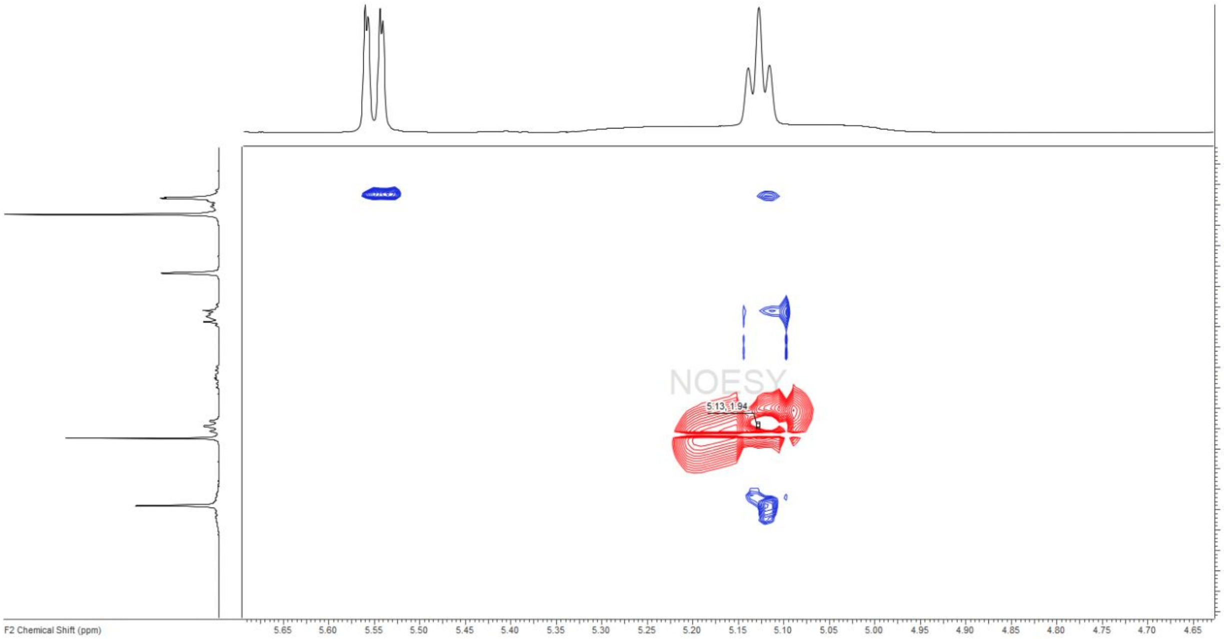

Figure S58: Tuaimenal G (**6**) zoomed NOESY NMR spectrum (500 MHz, $CDCl_3$).

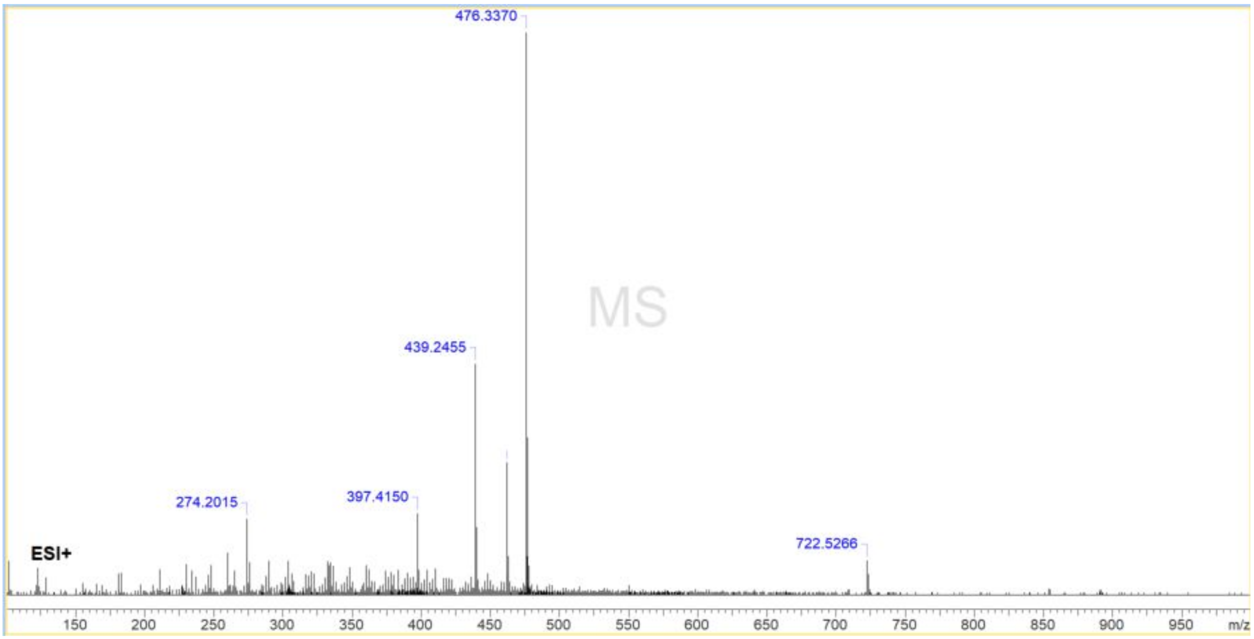

Figure S59: Tuaimenal G (**6**) HRESIMS (pos).

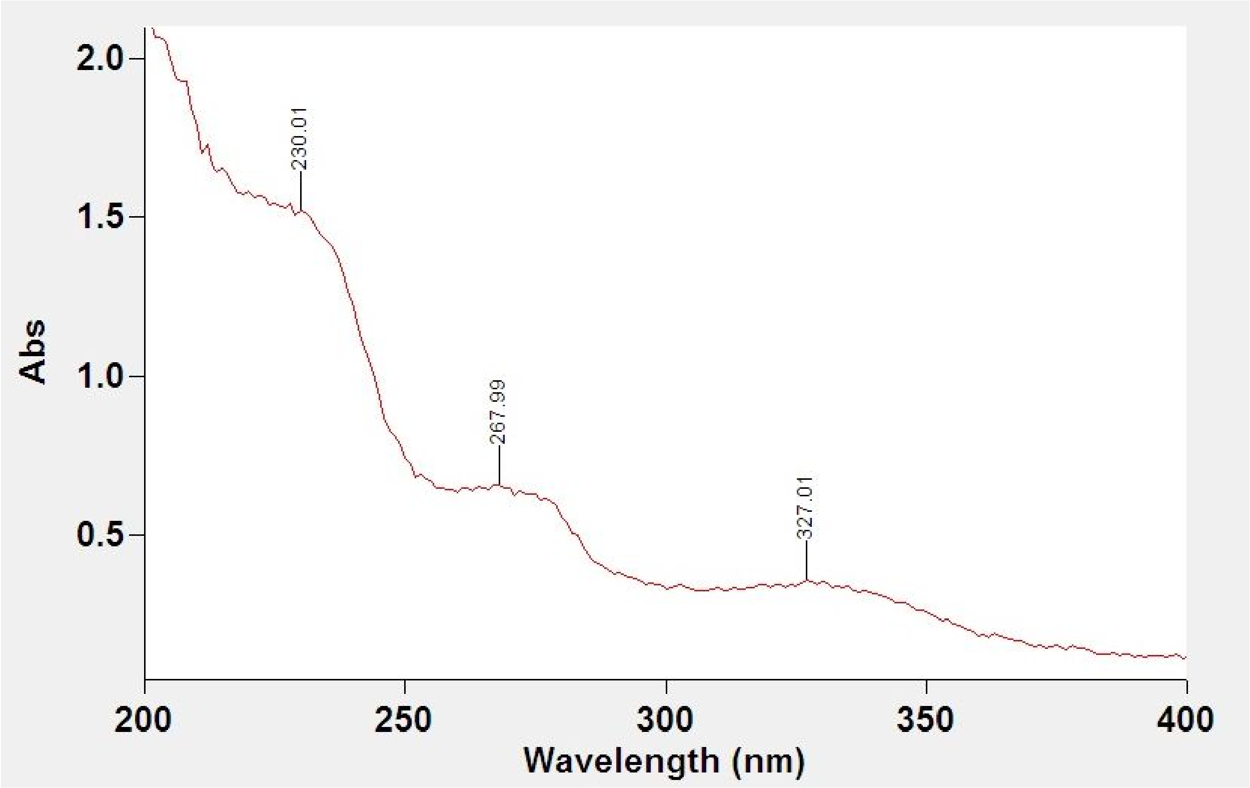

Figure S60: Tuaimenal G (**6**) UV λ_{max} (C_2H_3N).

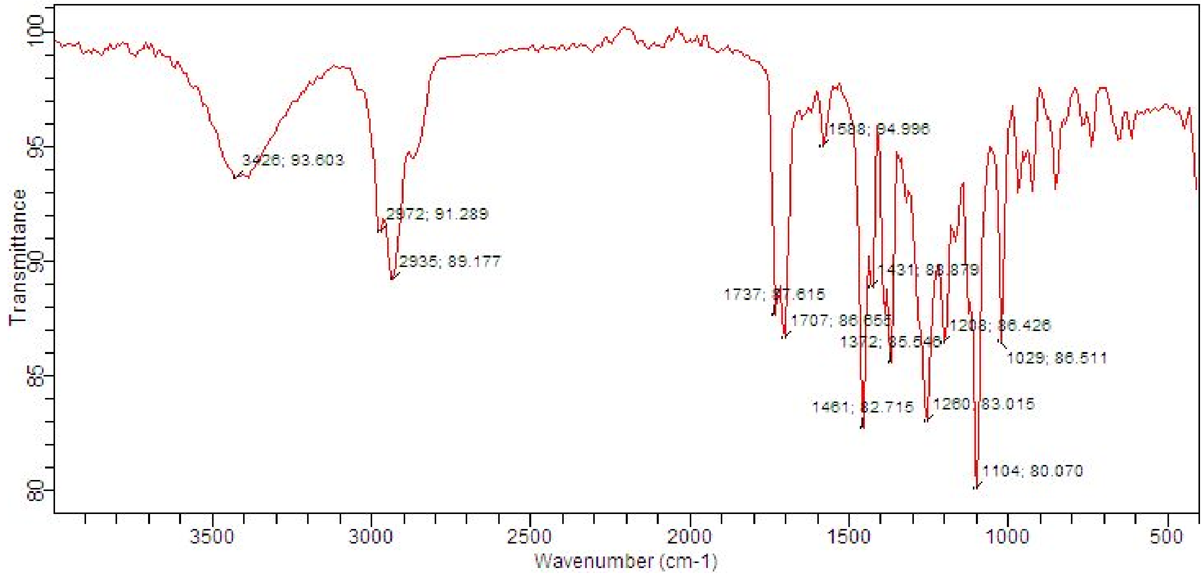

Figure S61: Tuaimenal G (**6**) IR spectrum (thin film).

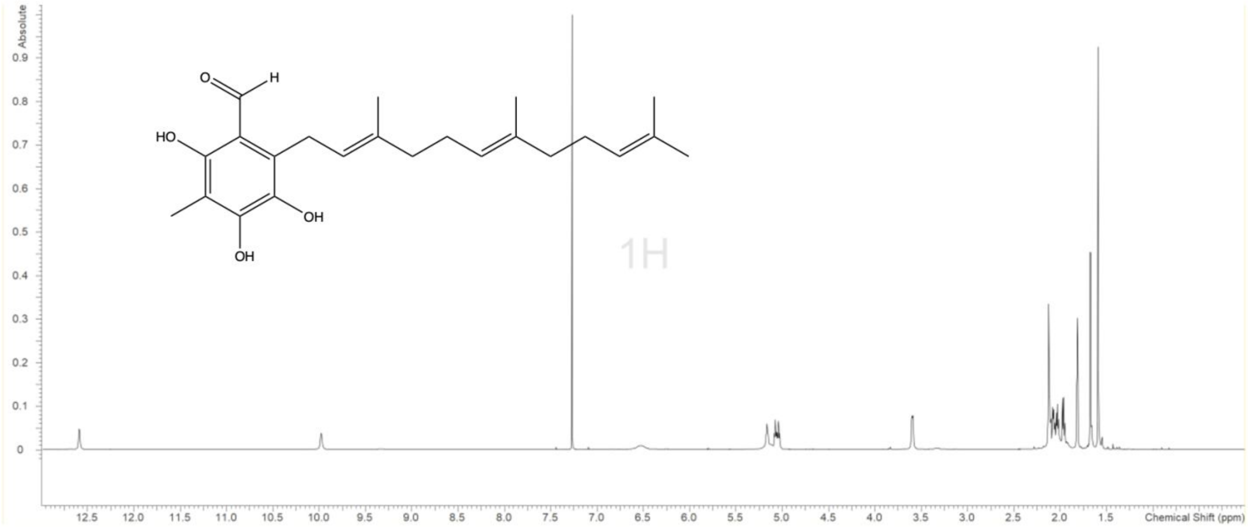

Figure S62: Tuaimenal H (**7**) ^{1}H NMR spectrum (600 MHz, $CDCl_3$).

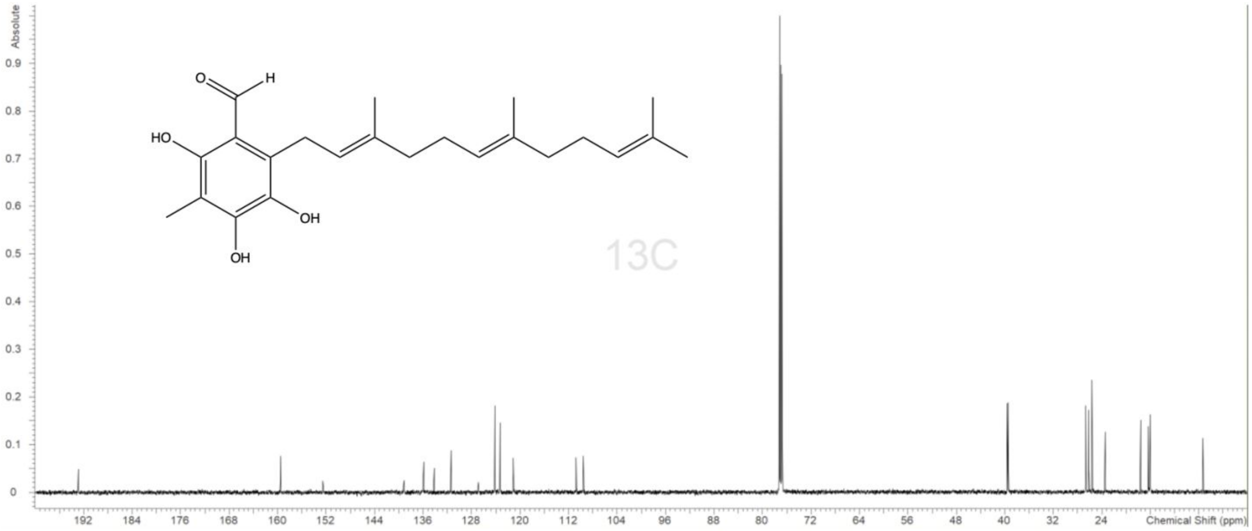

Figure S63: Tuaimenal H (**7**) ^{13}C NMR spectrum (150 MHz, $CDCl_3$).

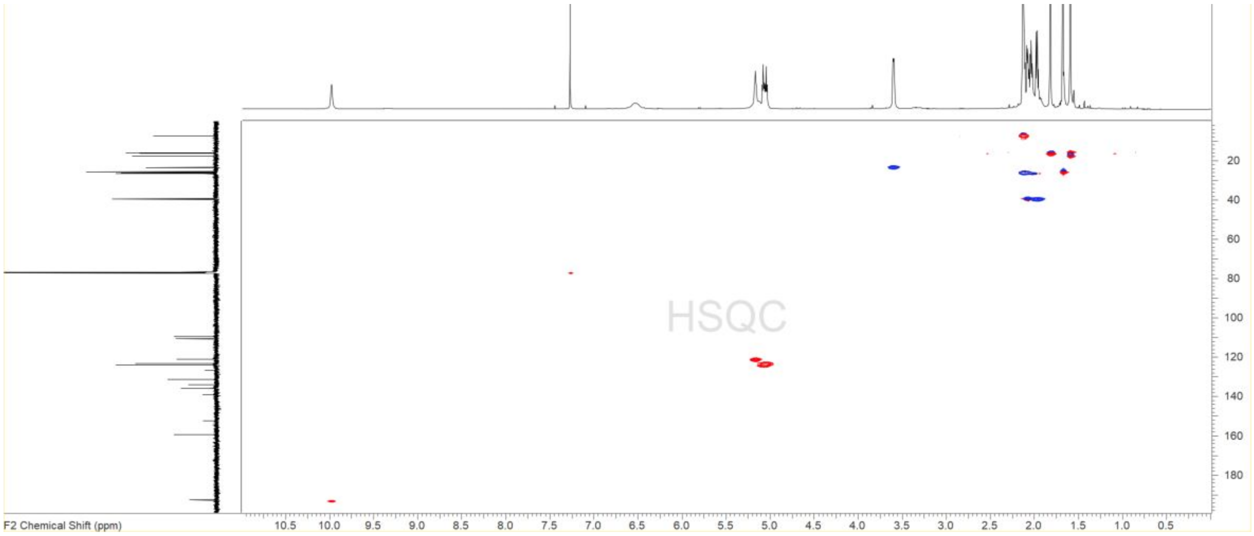

Figure S64: Tuaimenal H (**7**) HSCQ NMR spectrum (500 MHz, $CDCl_3$).

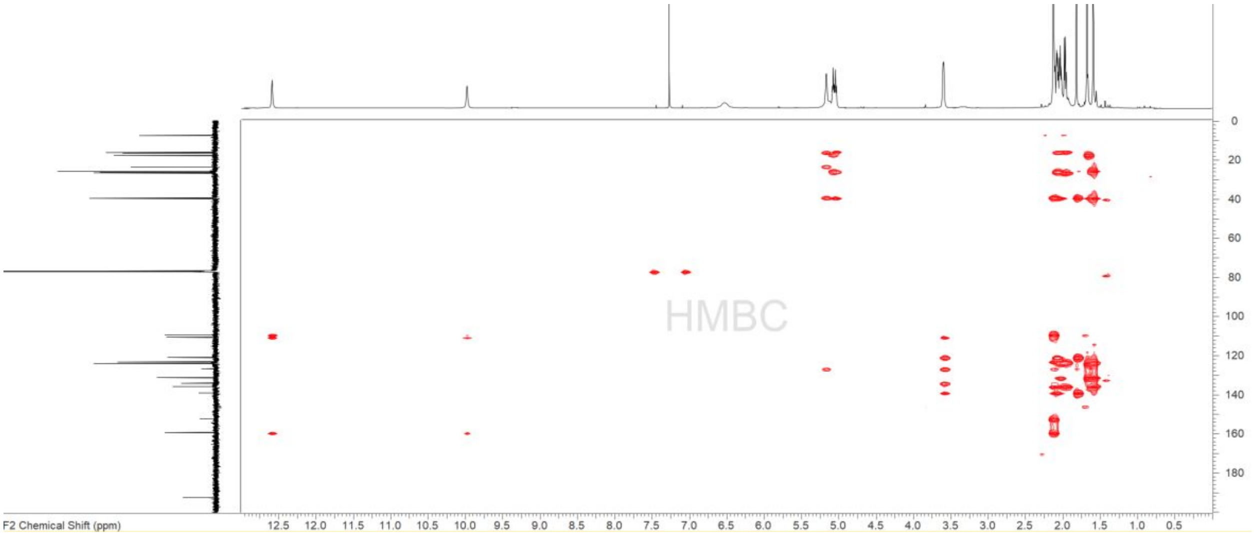

Figure S65: Tuaimenal H (**7**) HMBC NMR spectrum (500 MHz, $CDCl_3$).

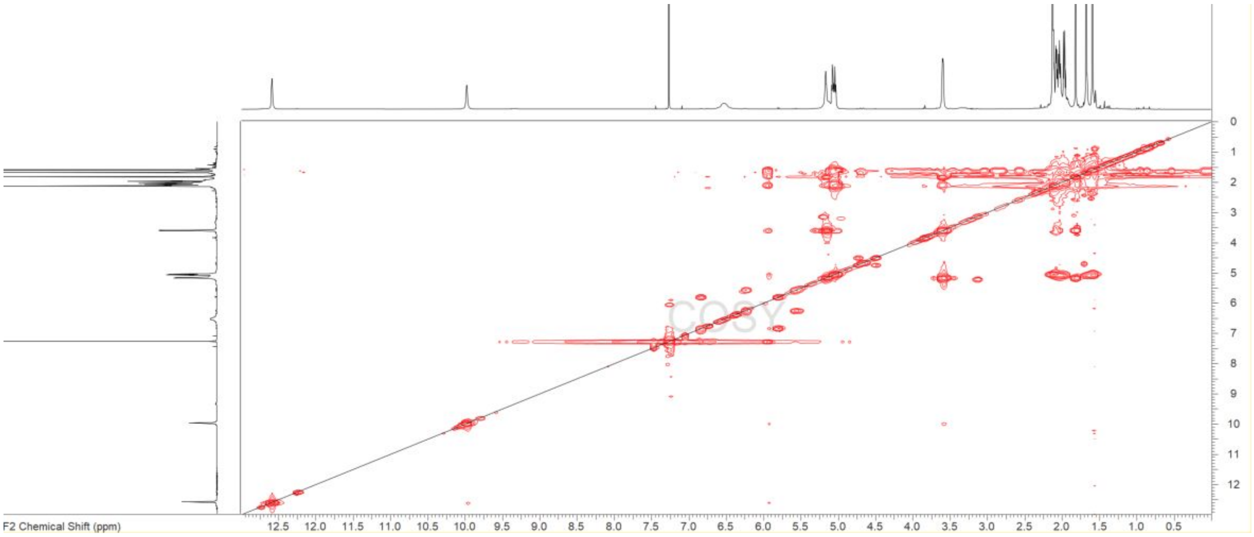

Figure S66: Tuaimenal H (**7**) COSY NMR spectrum (500 MHz, $CDCl_3$).

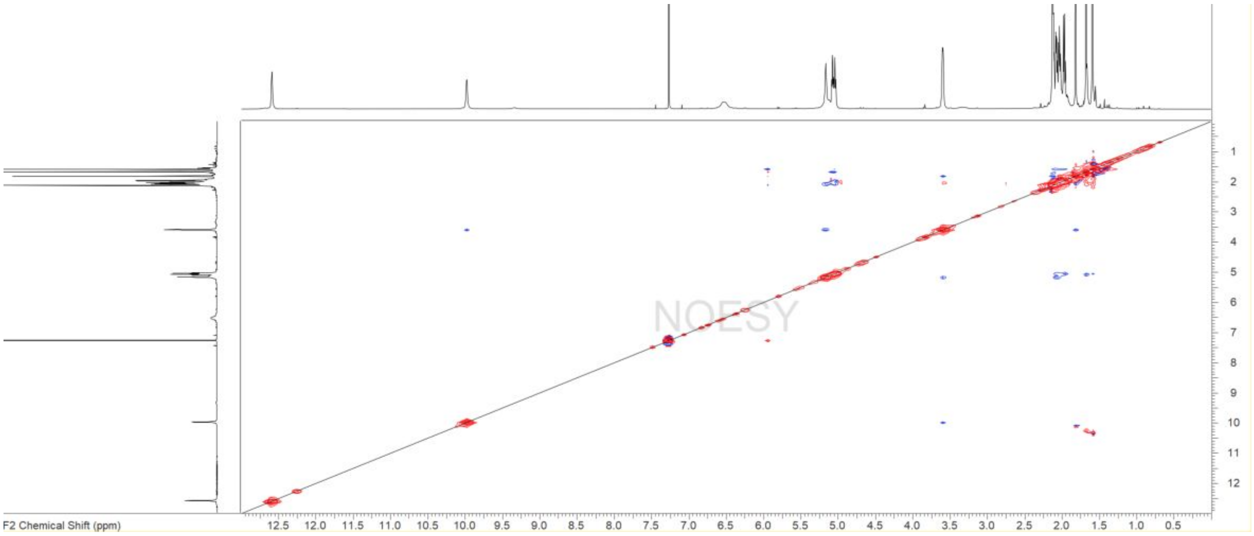

Figure S67: Tuaimenal H (**7**) NOESY NMR spectrum (500 MHz, $CDCl_3$).

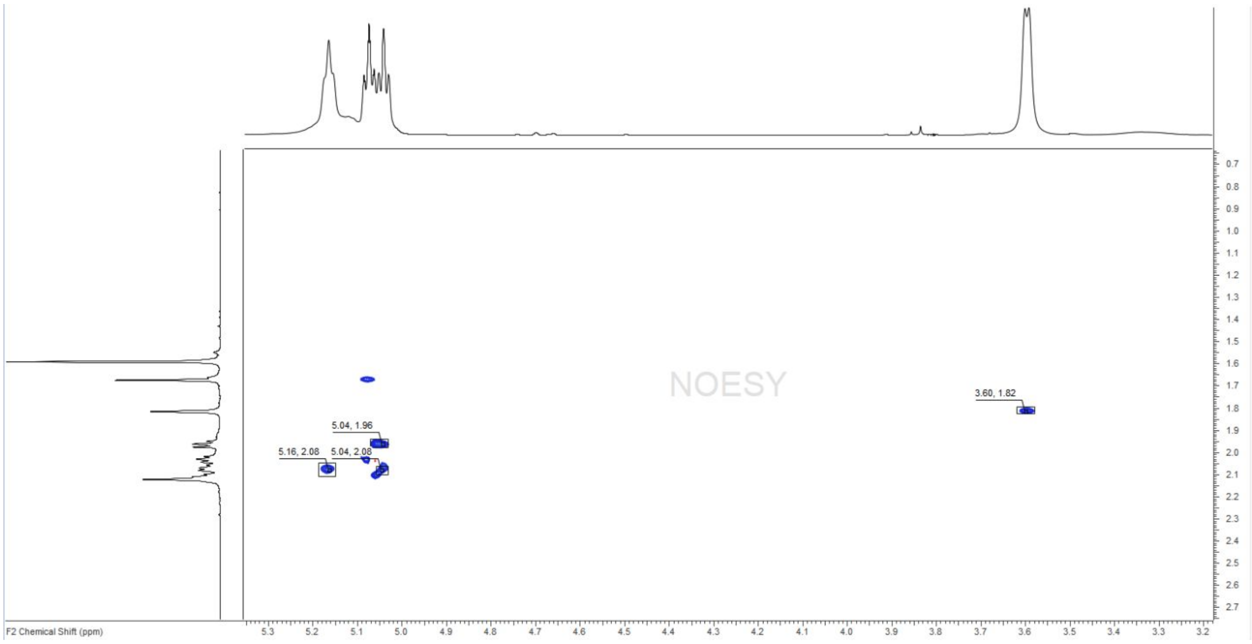

Figure S68: Tuaimenal H (**7**) zoomed NOESY NMR spectrum (500 MHz, $CDCl_3$).

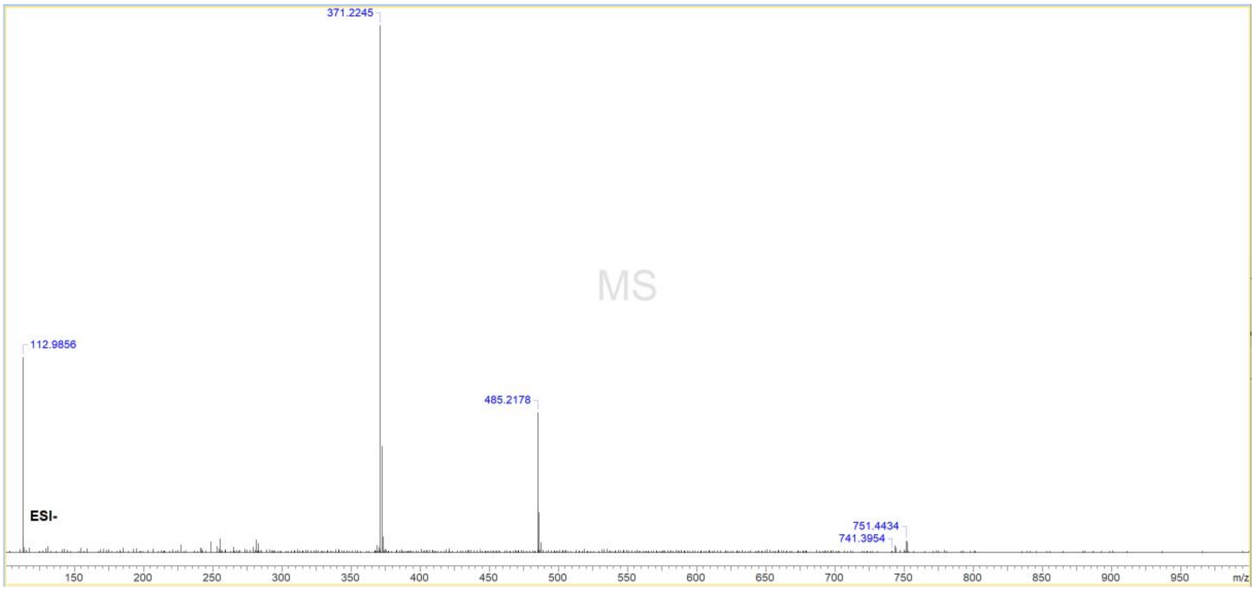

Figure S69: Tuaimenal H (**7**) HRESIMS (neg).

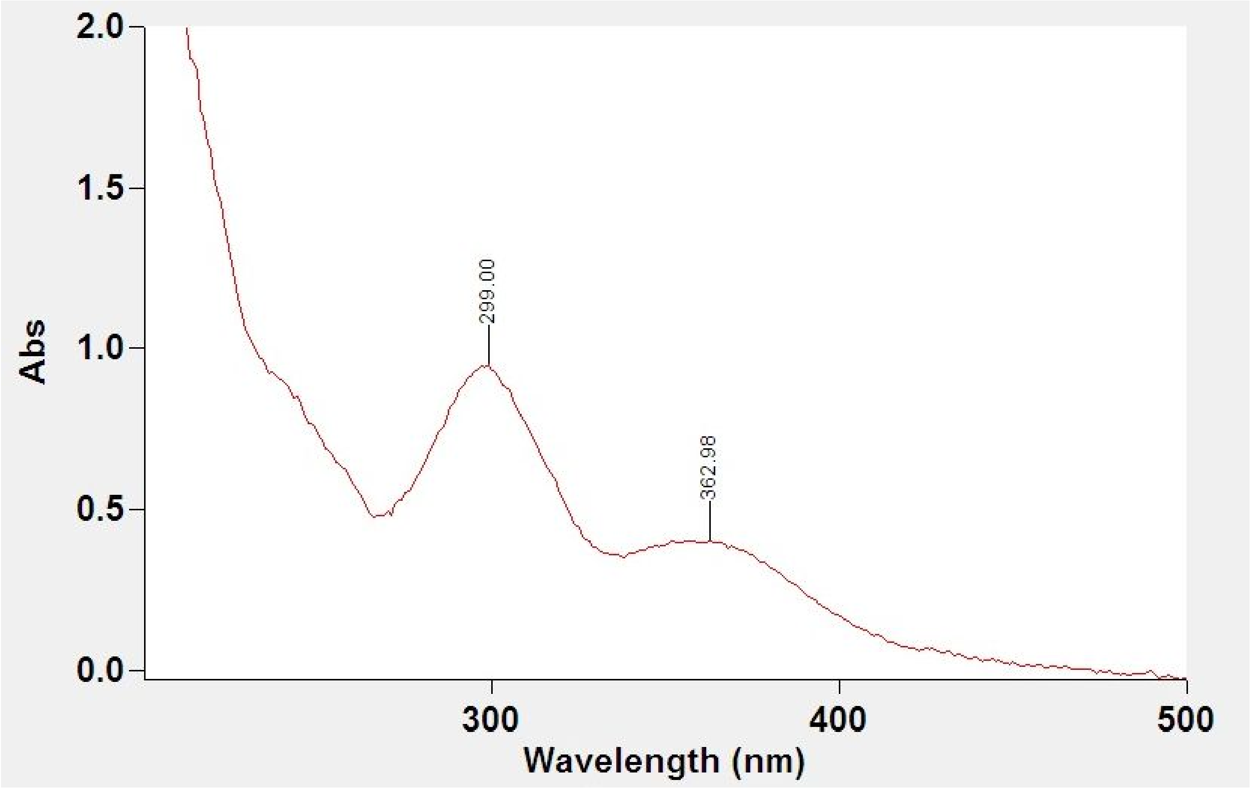

Figure S70: Tuaimenal H (**7**) UV λ_{max} (C_2H_3N).

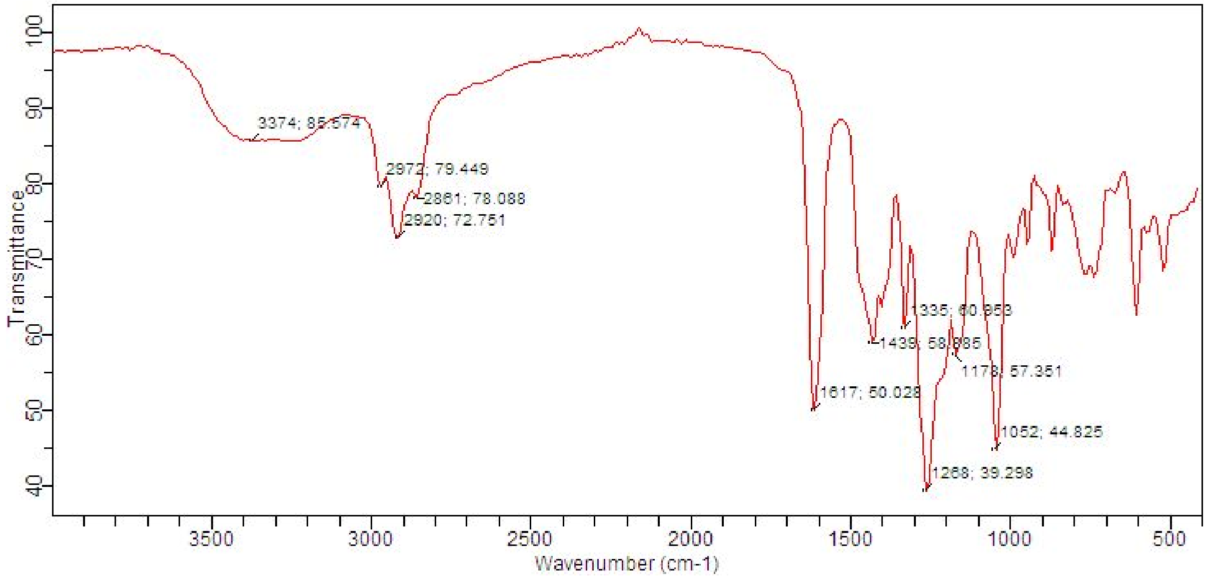

Figure S71: Tuaimenal H (**7**) IR spectrum (thin film).

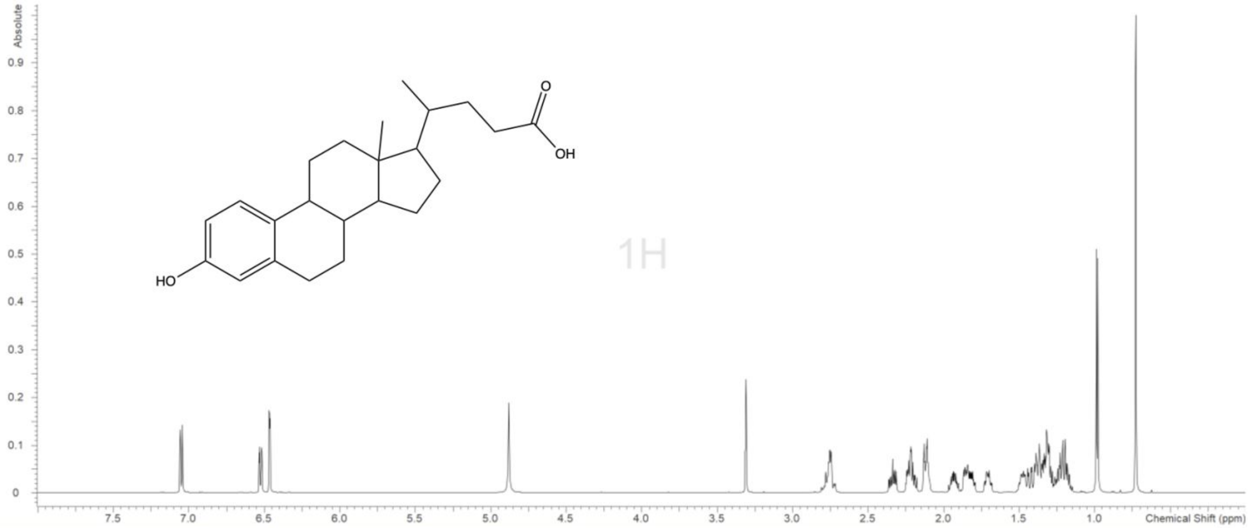

Figure S72: 3-hydroxy-19-norchola-1,3,5(10)-trien-24-oic acid (**8**) ^{1}H NMR spectrum (600 MHz, MeOD).

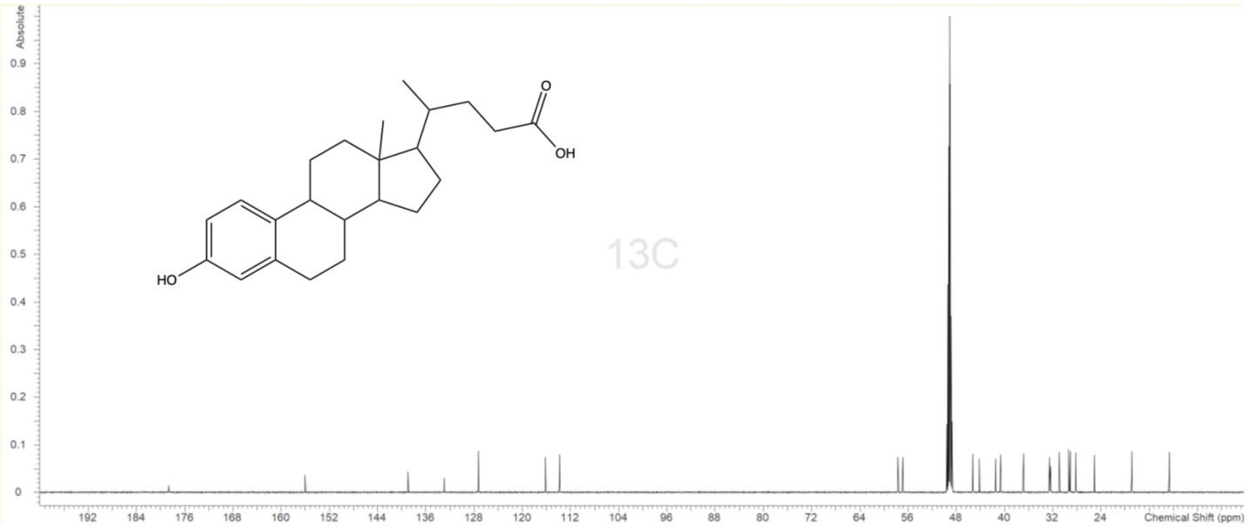

Figure S73: 3-hydroxy-19-norchola-1,3,5(10)-trien-24-oic acid (**8**) ^{13}C NMR spectrum (150 MHz, MeOD).

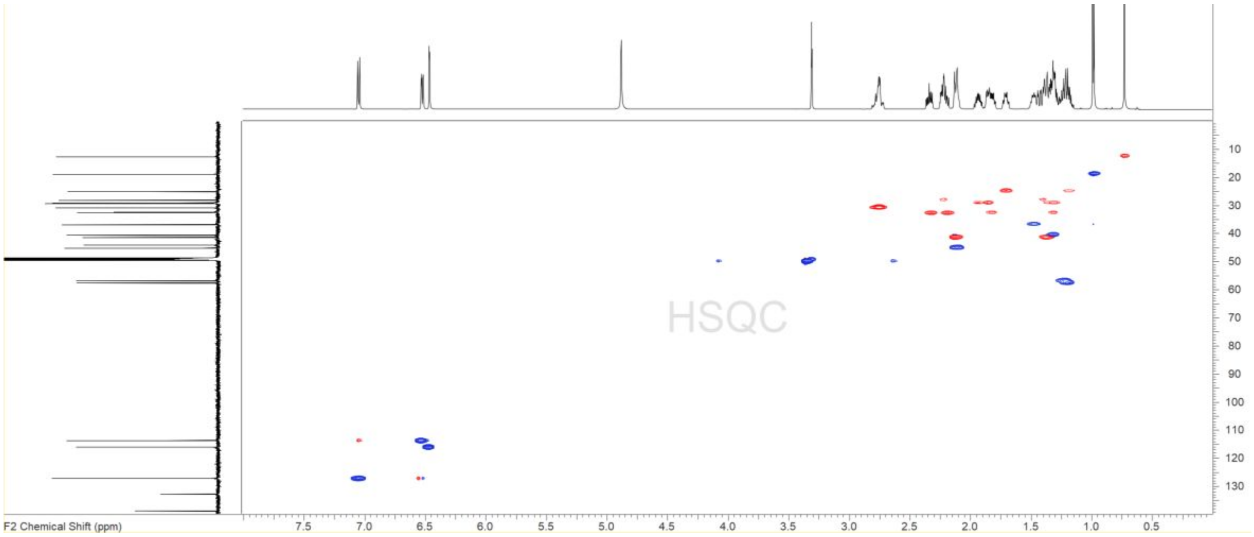

Figure S74: 3-hydroxy-19-norchola-1,3,5(10)-trien-24-oic acid (**8**) HSCQ NMR spectrum (500 MHz, MeOD).

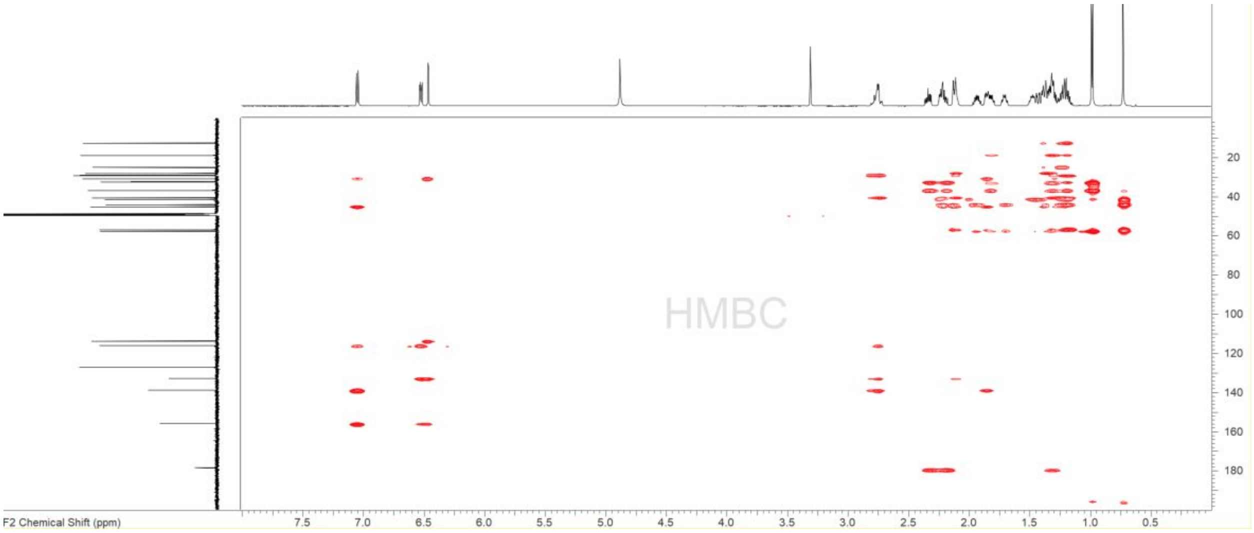

Figure S75: 3-hydroxy-19-norchola-1,3,5(10)-trien-24-oic acid (**8**) HMBC NMR spectrum (500 MHz, MeOD).

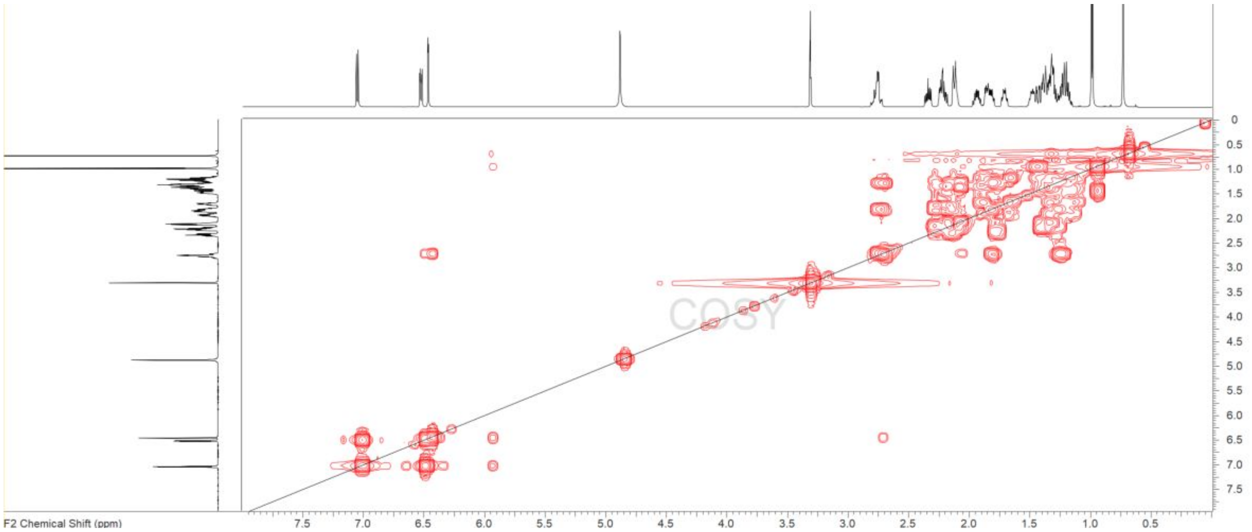

Figure S76: 3-hydroxy-19-norchola-1,3,5(10)-trien-24-oic acid (**8**) COSY NMR spectrum (500 MHz, MeOD).

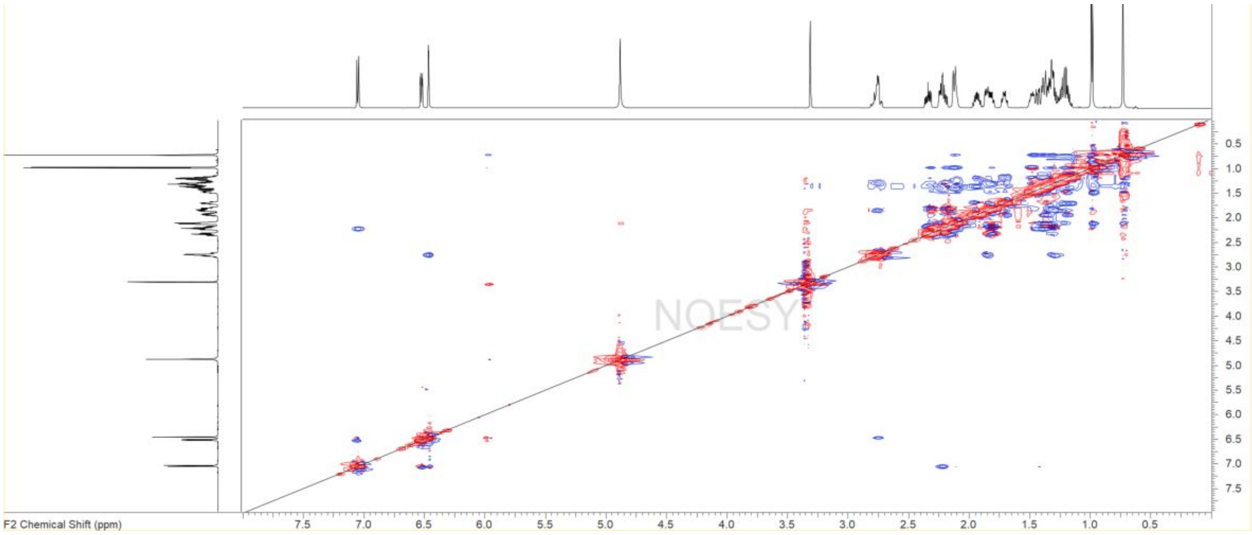

Figure S77: 3-hydroxy-19-norchola-1,3,5(10)-trien-24-oic acid (**8**) NOESY NMR spectrum (500 MHz, MeOD).

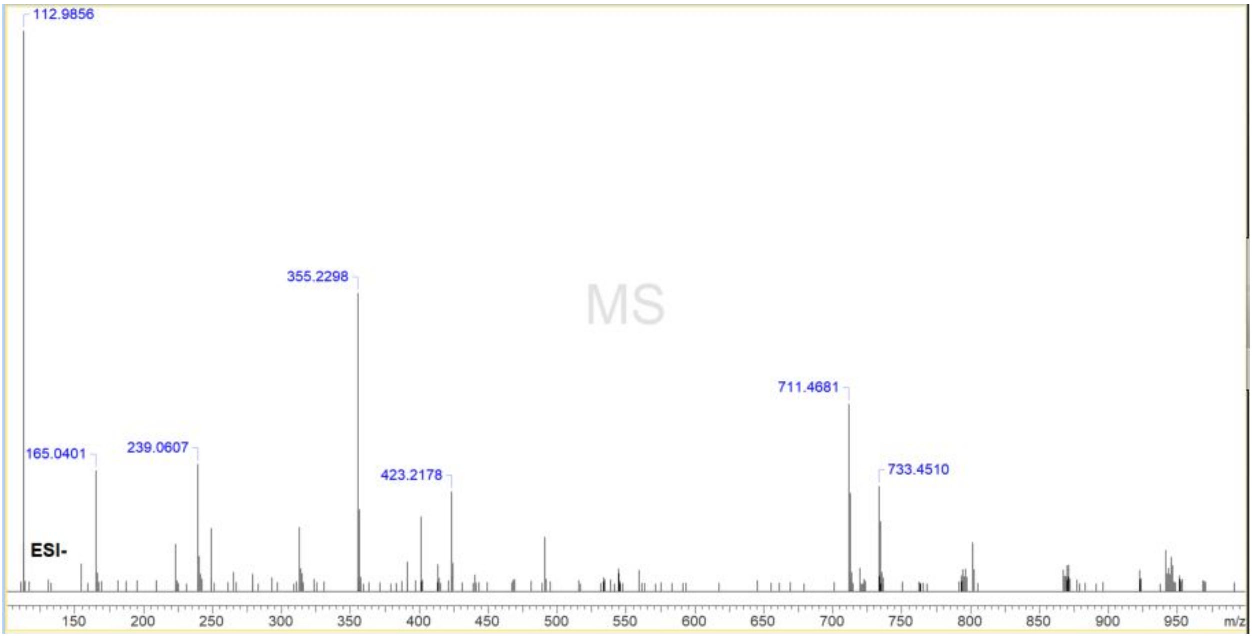

Figure S78: 3-hydroxy-19-norchola-1,3,5(10)-trien-24-oic acid (**8**) HRESIMS (neg).

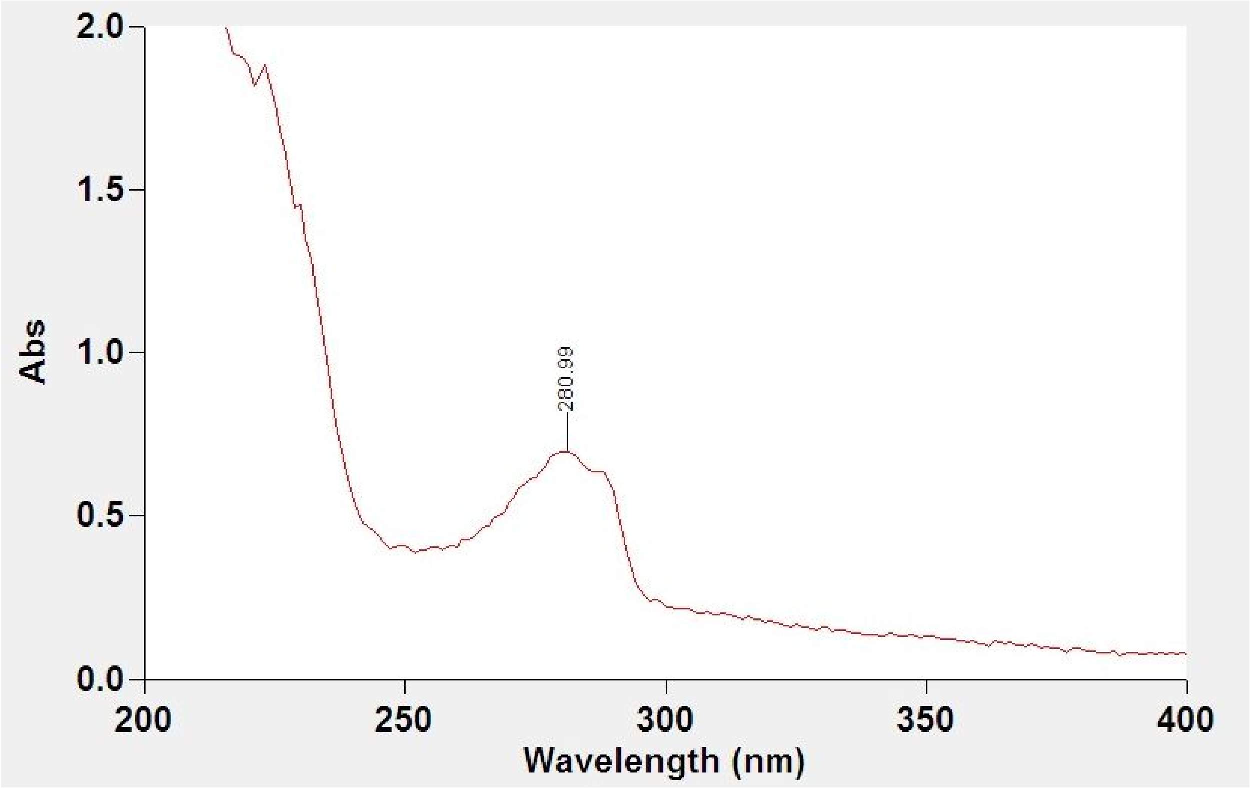

Figure S79: 3-hydroxy-19-norchola-1,3,5(10)-trien-24-oic acid (**8**) UV λ_{max} (C_2H_3N).

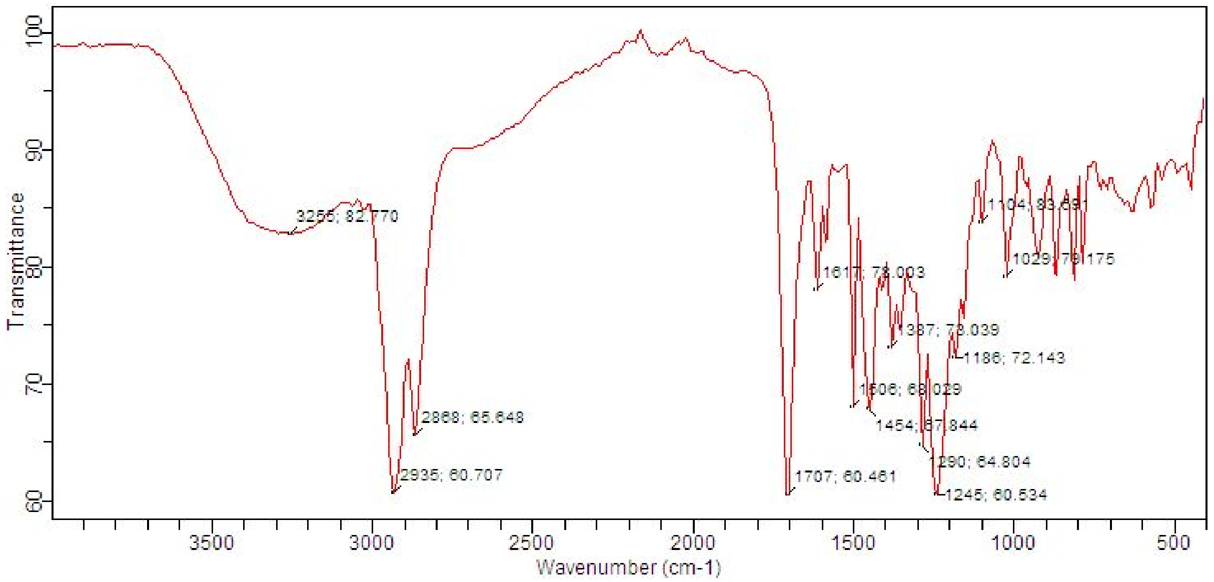

Figure S80: 3-hydroxy-19-norchola-1,3,5(10)-trien-24-oic acid (**8**) IR spectrum (thin film).

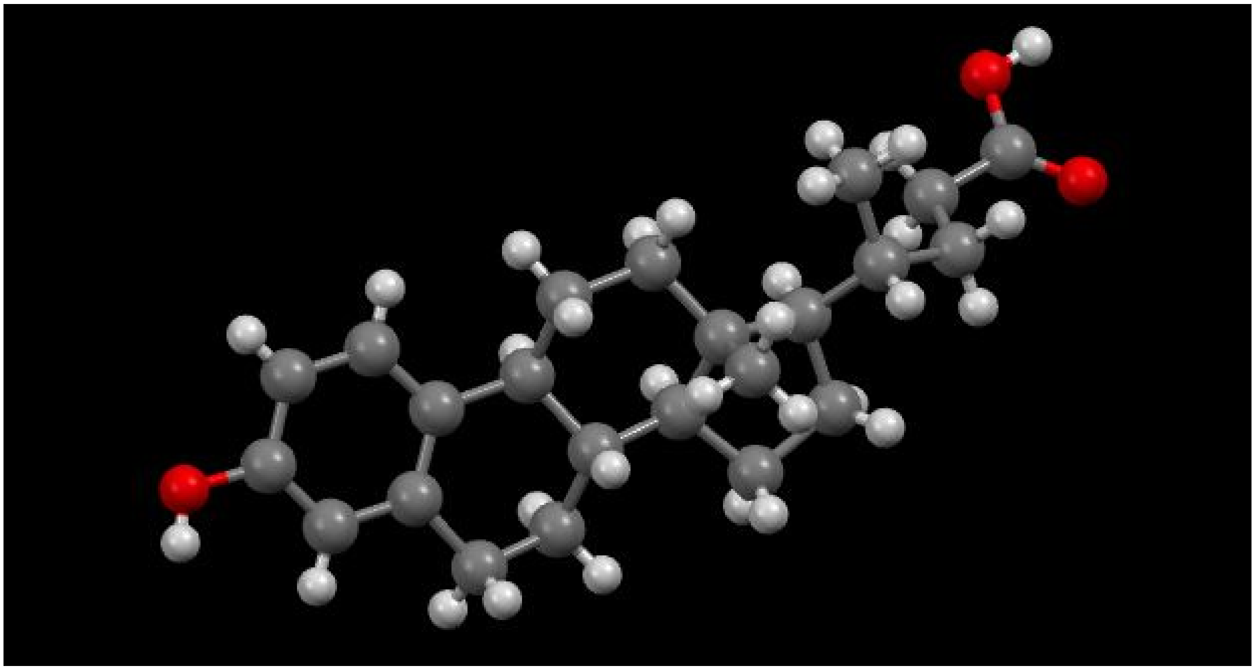

Figure S81: 3-hydroxy-19-norchola-1,3,5(10)-trien-24-oic acid (**8**) X-ray crystal structure.

Identification code	JTW-045
Empirical formula	$C_{23}H_{32}O_3$
Formula weight	356.48
Temperature/K	100
Crystal system	orthorhombic
Space group	$P2_12_12_1$
a/Å	6.46790(10)
b/Å	9.6023(2)
c/Å	30.7076(5)
α/°	90
β/°	90
γ/°	90
Volume/Å^3	1907.15(6)
Z	4
ρ_{calc}g/cm^3	1.242
μ/mm^{-1}	0.629
F(000)	776.0
Crystal size/mm^3	0.2 × 0.2 × 0.1
Radiation	CuKα (λ = 1.54178)
2Θ range for data collection/°	5.756 to 158.692
Index ranges	-8 ≤ h ≤ 8, -12 ≤ k ≤ 12, -38 ≤ l ≤ 38
Reflections collected	44886
Independent reflections	4106 [R_{int} = 0.0296, R_{sigma} = 0.0141]
Data/restraints/parameters	4106/0/245
Goodness-of-fit on F^2	1.080
Final R indexes [I>=2σ (I)]	R_1 = 0.0344, wR_2 = 0.0916
Final R indexes [all data]	R_1 = 0.0347, wR_2 = 0.0919
Largest diff. peak/hole / e Å^{-3}	0.16/-0.18
Flack parameter	0.00(3)

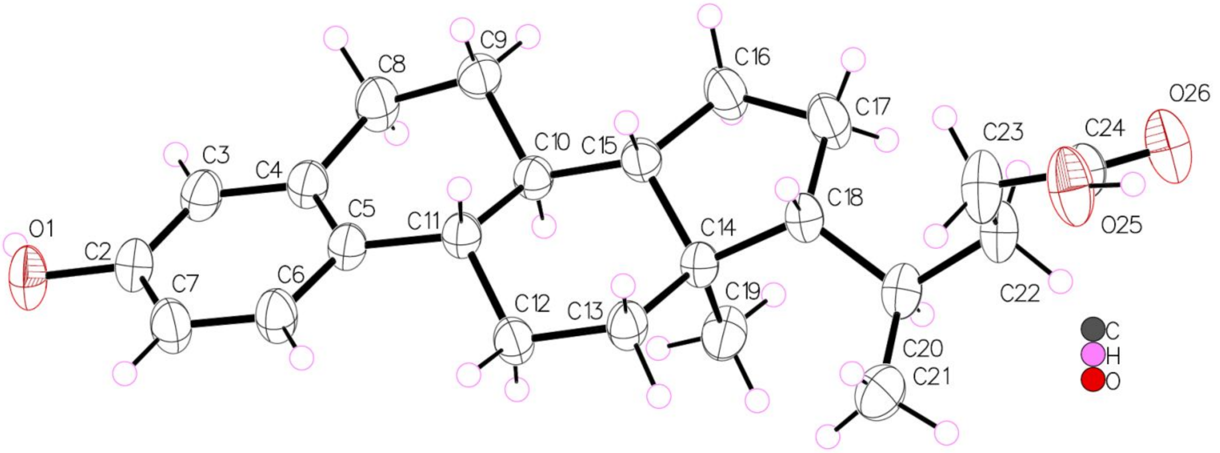

Figure S82: 3-hydroxy-19-norchola-1,3,5(10)-trien-24-oic acid (**8**) X-ray crystallography metadata and ellipsoid plot with anisotropic displacement parameters drawn at 50% probability.

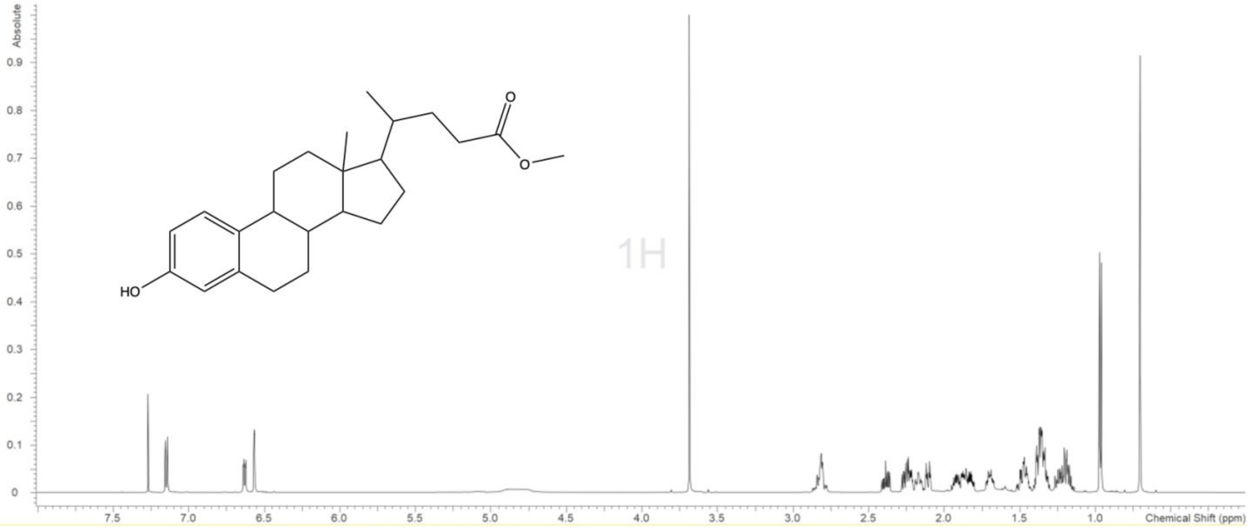

Figure S83: Methyl 3-hydroxy-19-norchola-1,3,5(10)-trien-24-oic acid (**9**) ^{1}H NMR spectrum (600 MHz, $CDCl_3$).

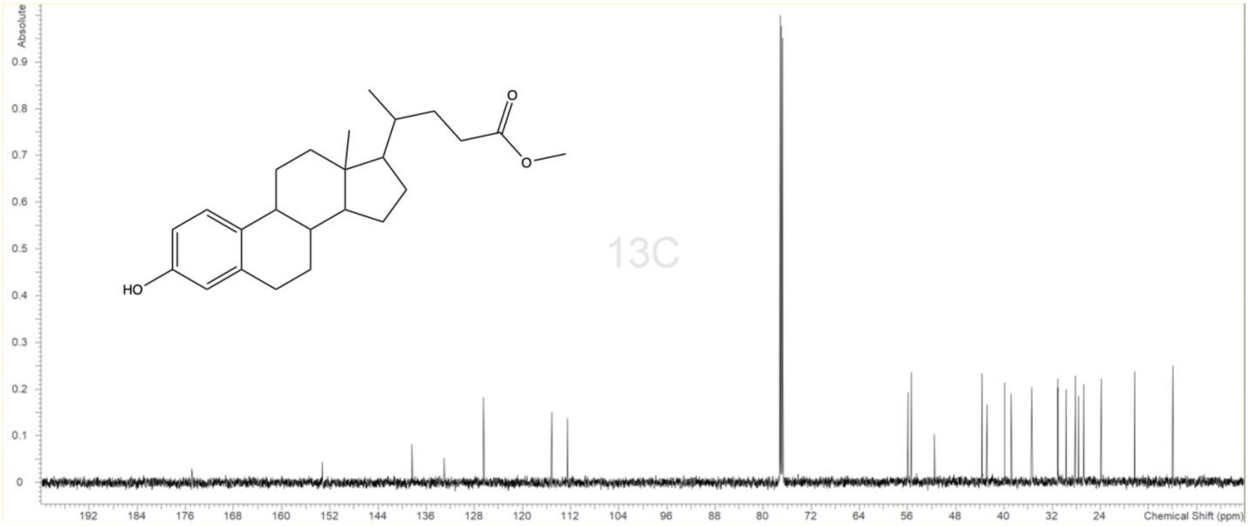

Figure S84: Methyl 3-hydroxy-19-norchola-1,3,5(10)-trien-24-oic acid (**9**) ^{13}C NMR spectrum (150 MHz, $CDCl_3$).

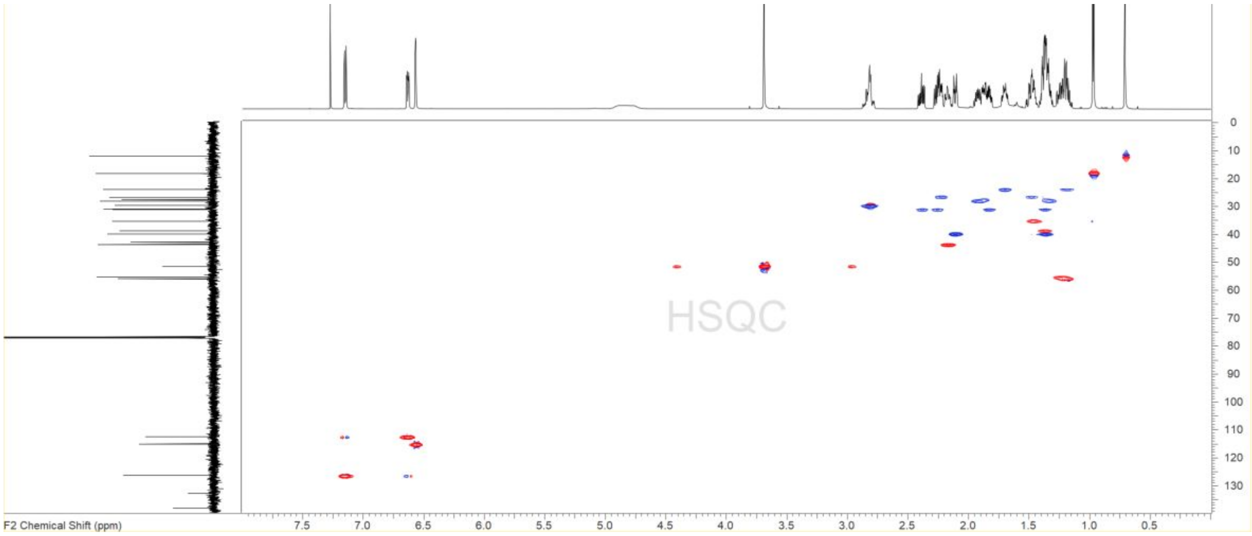

Figure S85: Methyl 3-hydroxy-19-norchola-1,3,5(10)-trien-24-oic acid (**9**) HSCQ NMR spectrum (500 MHz, $CDCl_3$).

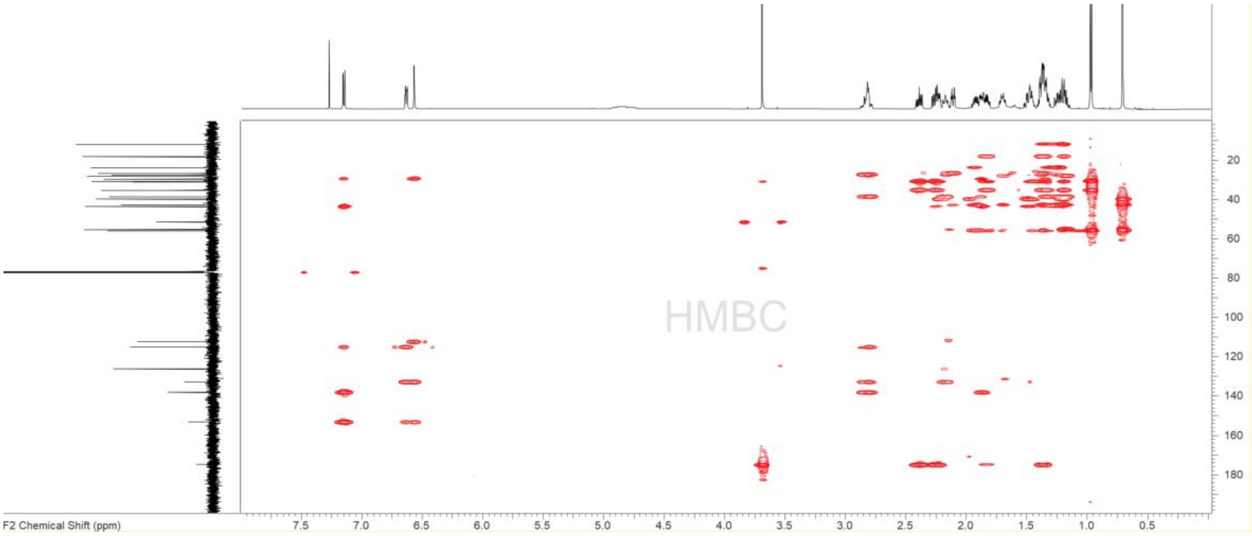

Figure S86: Methyl 30hydroxy-19-norchola-1,3,5(10)-trien-24-oic acid (**9**) HMBC NMR spectrum (500 MHz, $CDCl_3$).

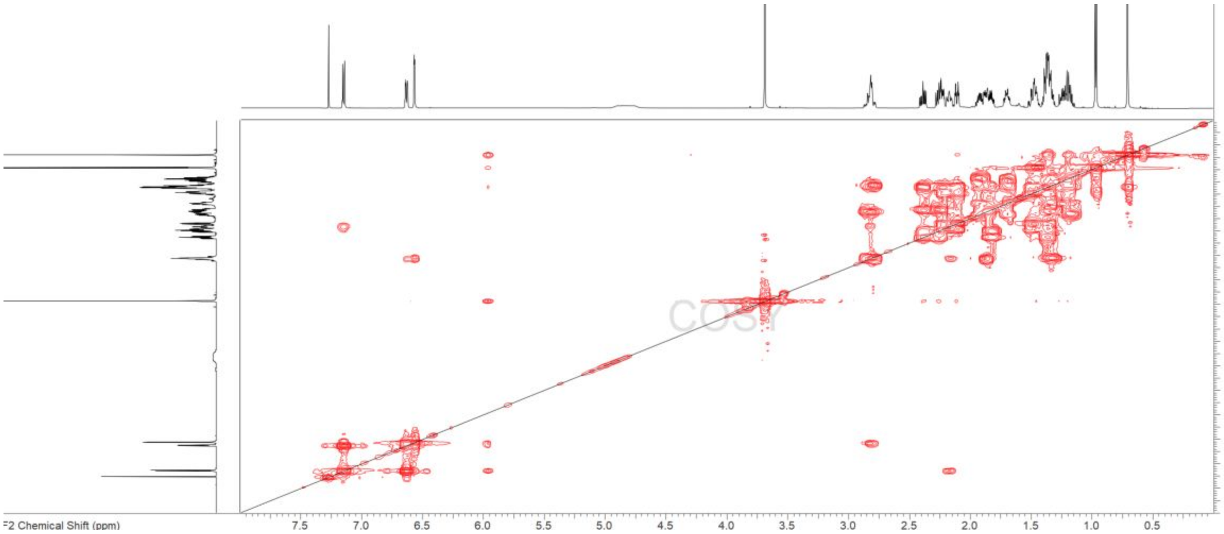

Figure S87: Methyl 3-hydroxy-19-norchola-1,3,5(10)-trien-24-oic acid (**9**) COSY NMR spectrum (500 MHz, $CDCl_3$).

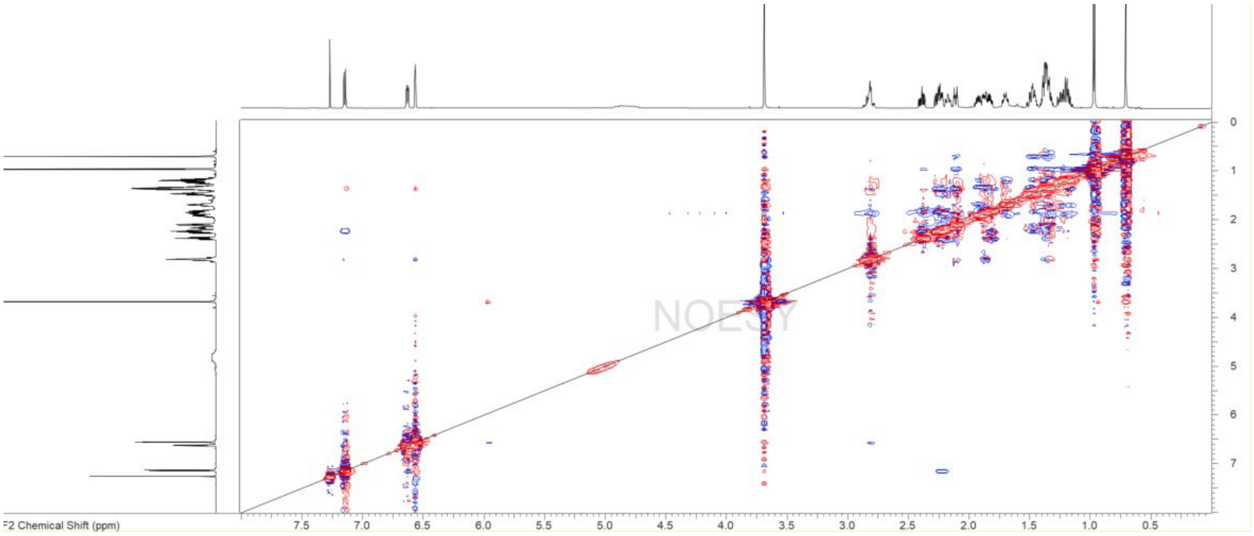

Figure S88: Methyl 3-hydroxy-19-norchola-1,3,5(10)-trien-24-oic acid (**9**) NOESY NMR spectrum (500 MHz, $CDCl_3$).

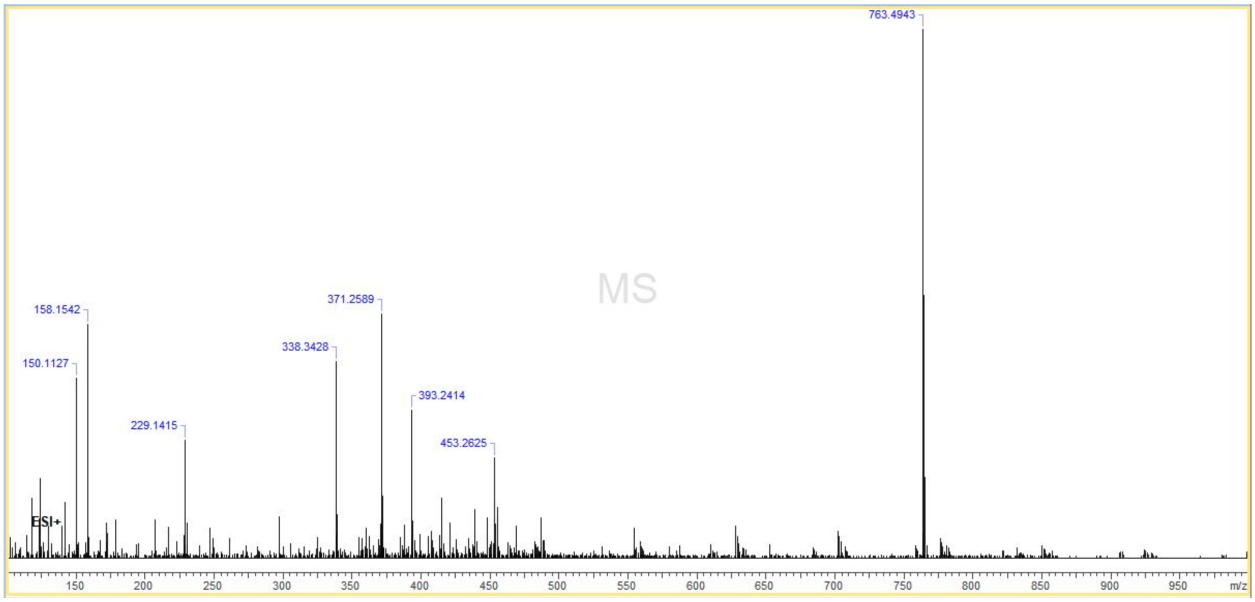

Figure S89: Methyl 3-hydroxy-19-norchola-1,3,5(10)-trien-24-oic acid (**9**) HRESIMS (pos).

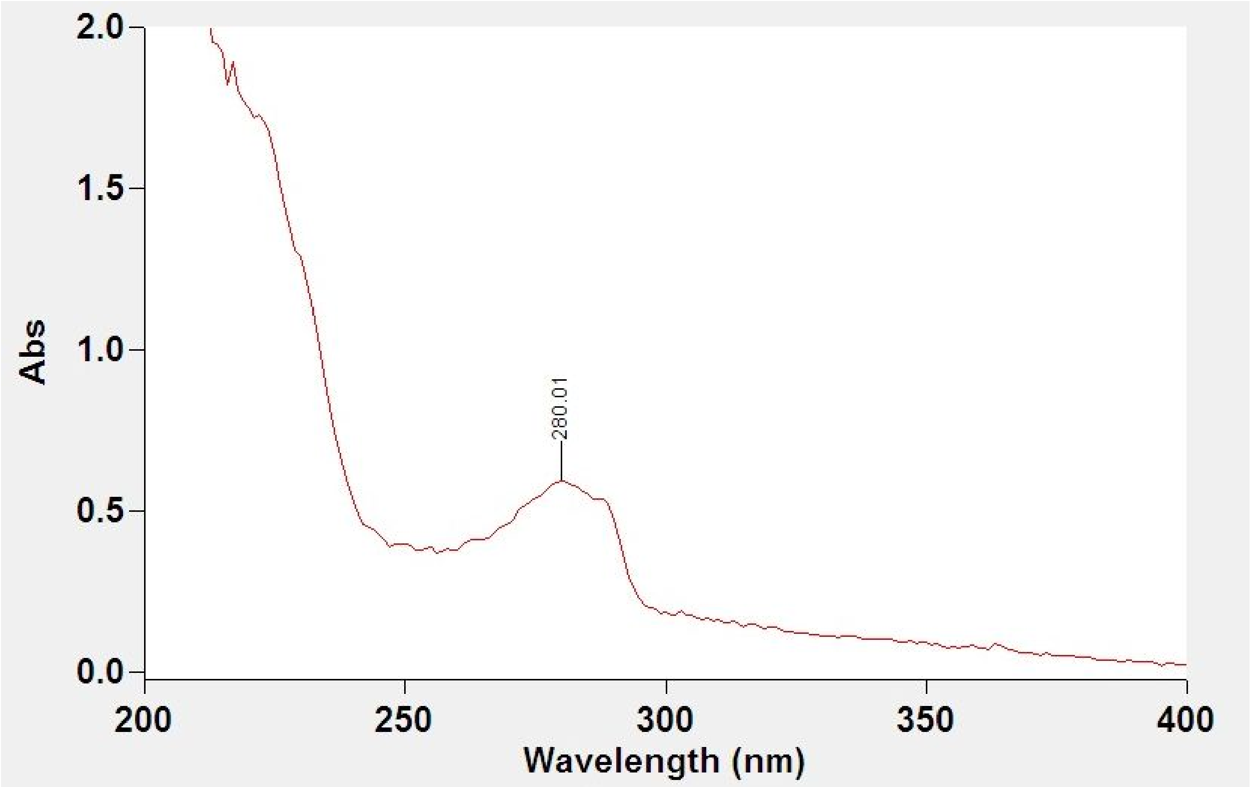

Figure S90: Methyl 3-hydroxy-19-norchola-1,3,5(10)-trien-24-oic acid (**9**) UV λ_{max} (C_2H_3N).

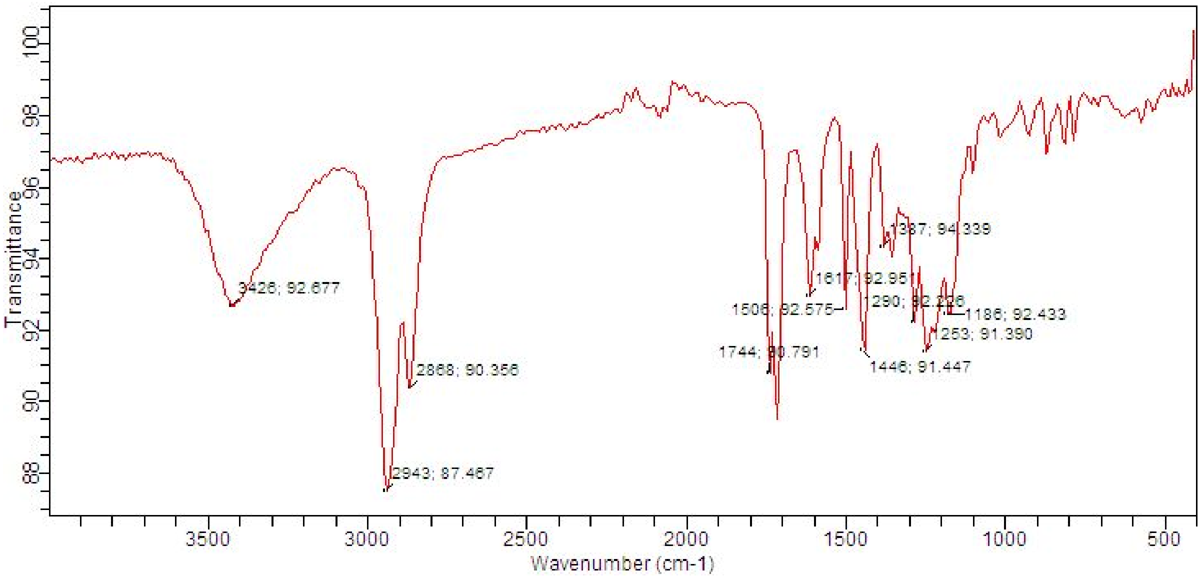

Figure S91: Methyl 3-hydroxy-19-norchola-1,3,5(10)-trien-24-oic acid (**9**) IR spectrum (thin film).

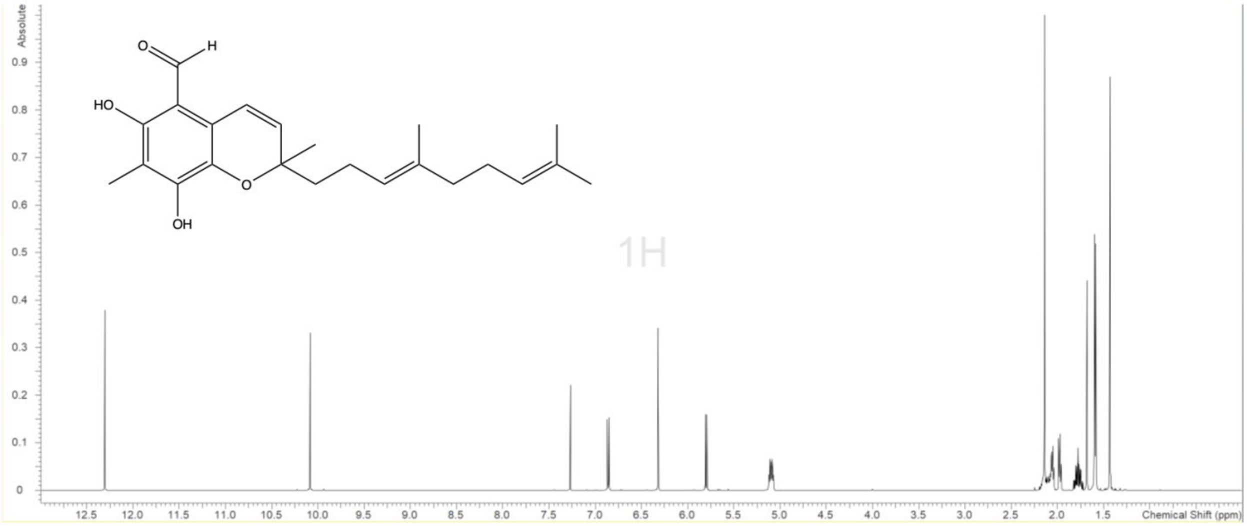

Figure S92: Tuaimenal A (**10**) ^{1}H NMR spectrum (600 MHz, $CDCl_3$).

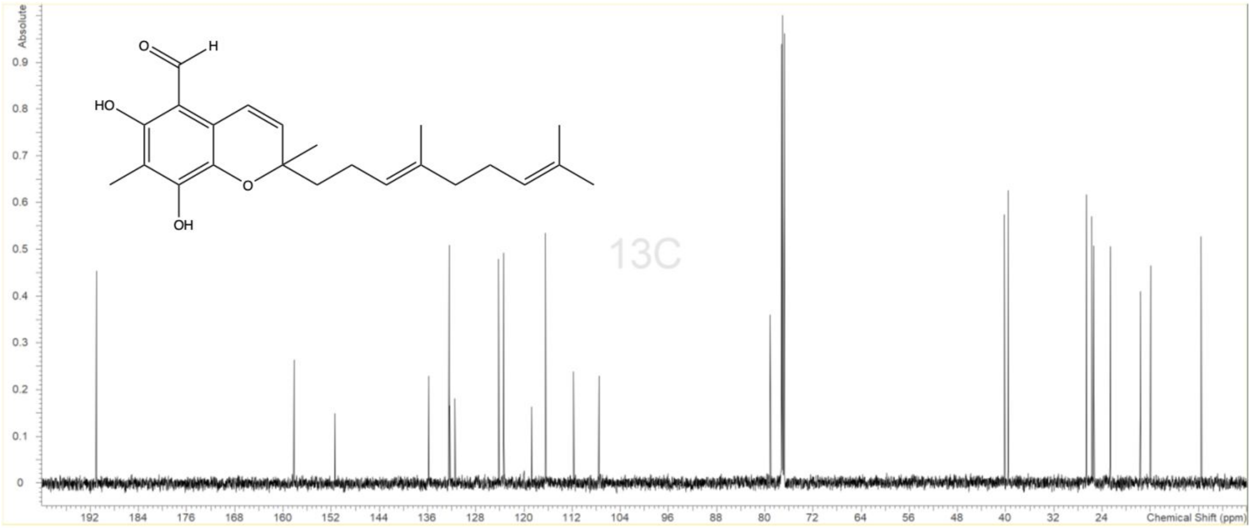

Figure S93: Tuaimenal A (**10**) ^{13}C NMR spectrum (150 MHz, $CDCl_3$).

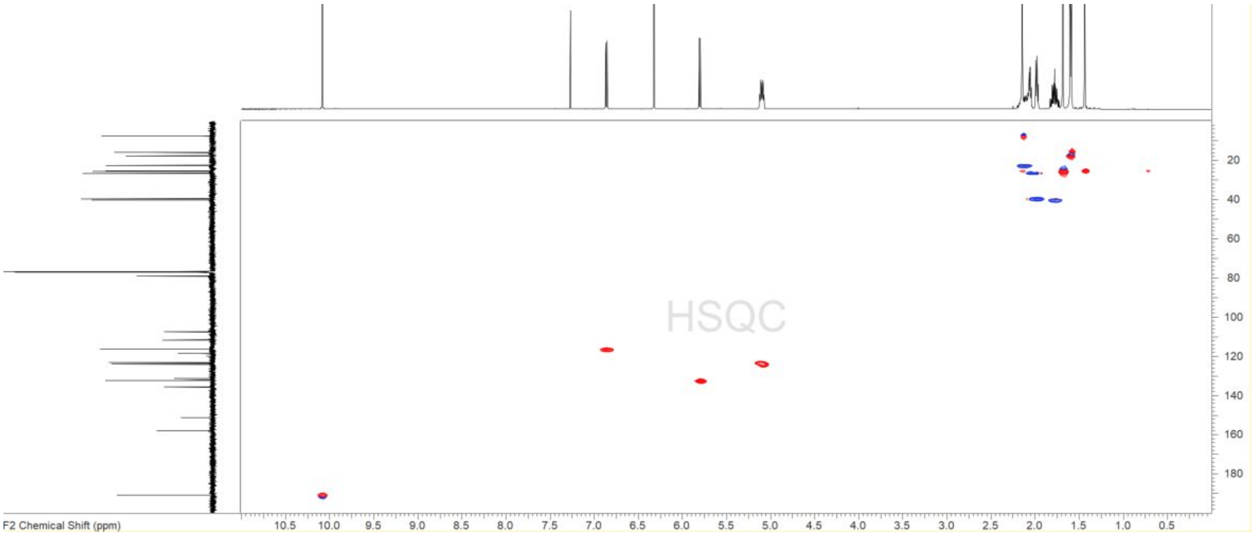

Figure S94: Tuaimenal A (**10**) HSCQ NMR spectrum (500 MHz, $CDCl_3$).

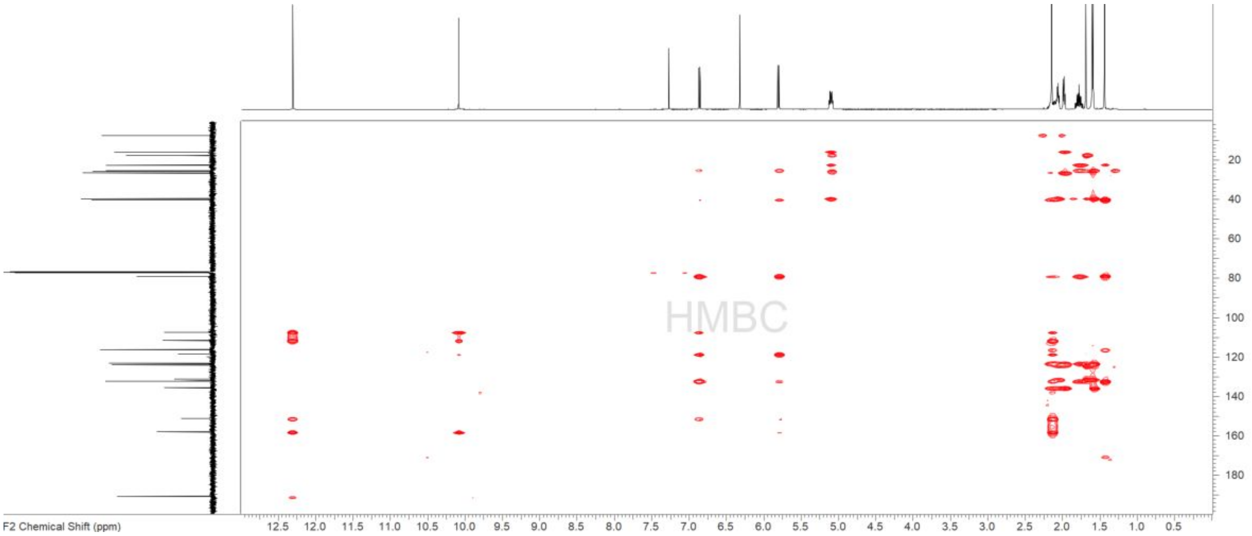

Figure S95: Tuaimenal A (**10**) HMBC NMR spectrum (500 MHz, $CDCl_3$).

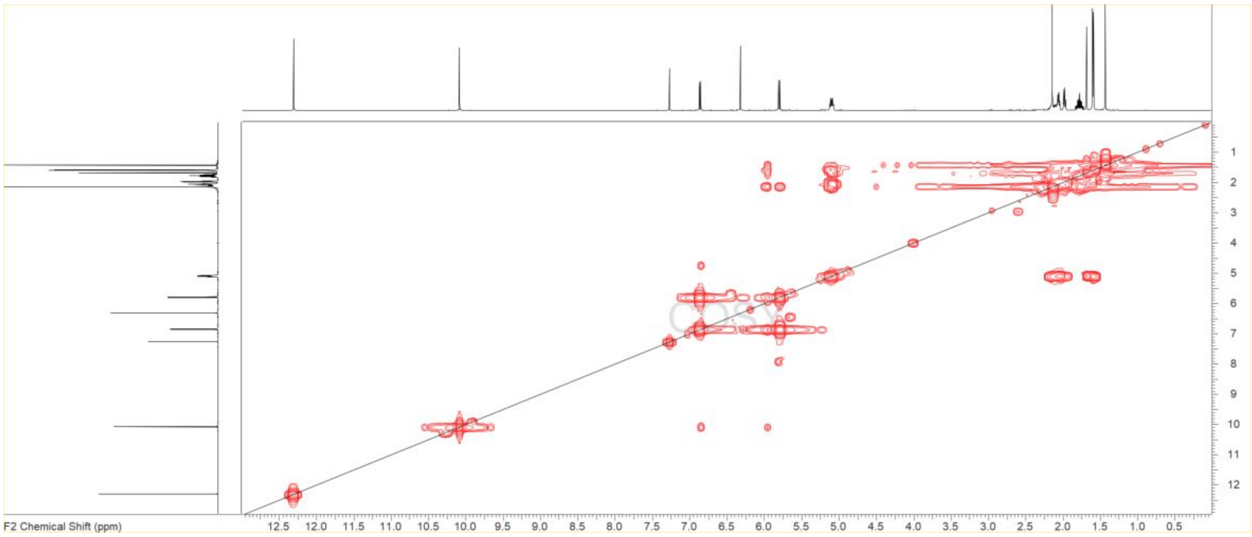

Figure S96: Tuaimenal A (**10**) COSY NMR spectrum (500 MHz, $CDCl_3$).

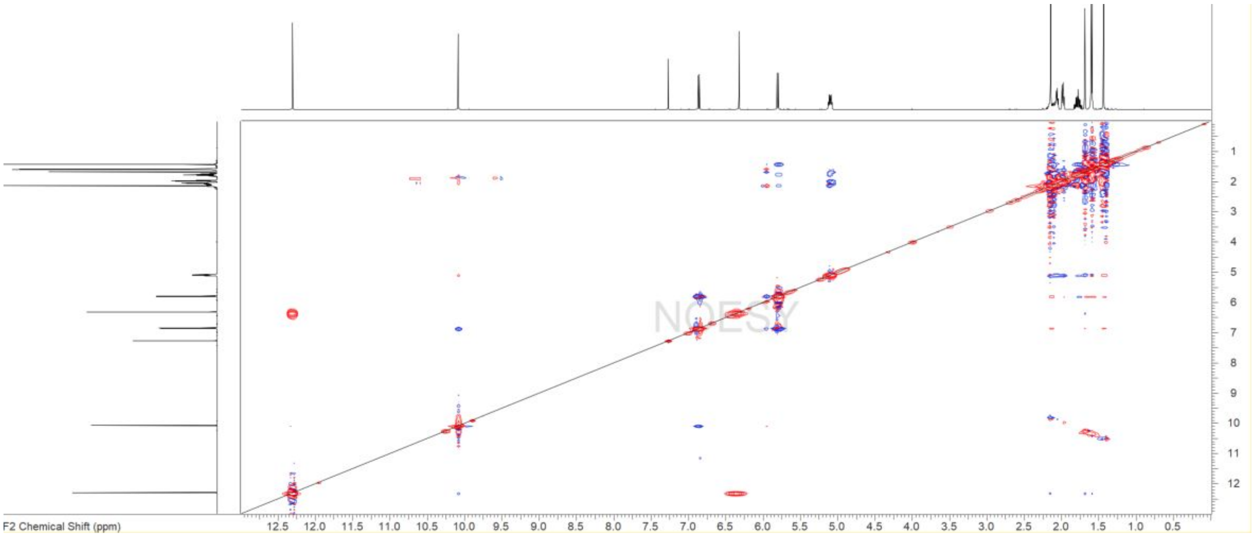

Figure S97: Tuaimenal A (**10**) NOESY NMR spectrum (500 MHz, $CDCl_3$).

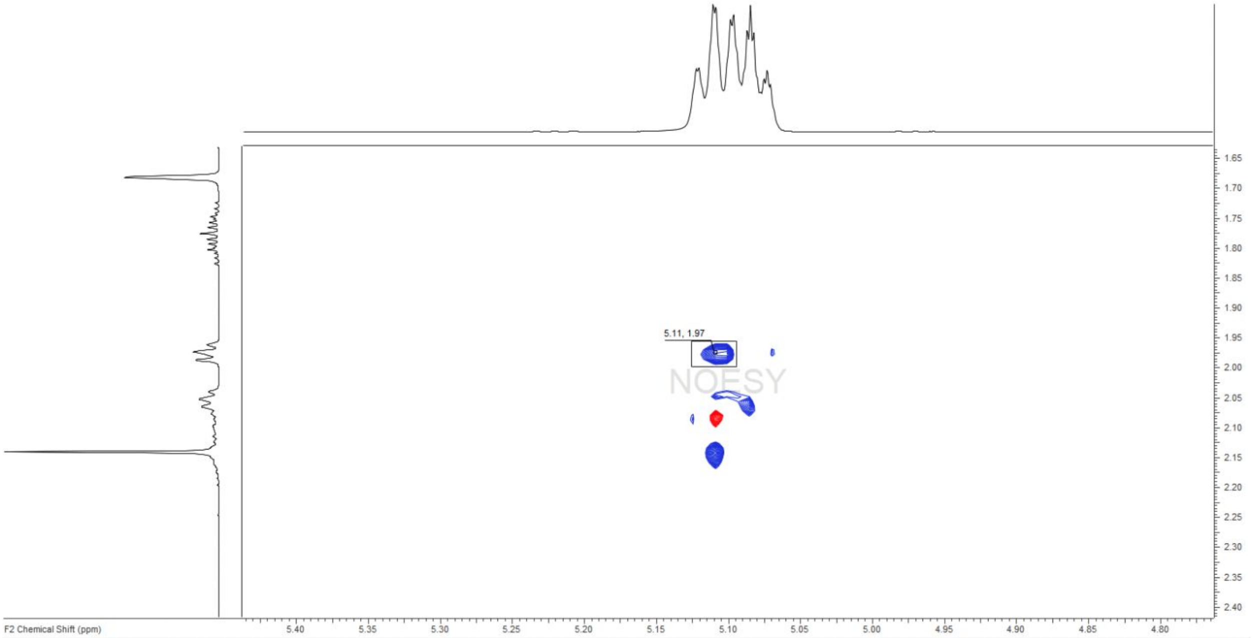

Figure S98: Tuaimenal A (**10**) zoomed NOESY NMR spectrum (500 MHz, $CDCl_3$).

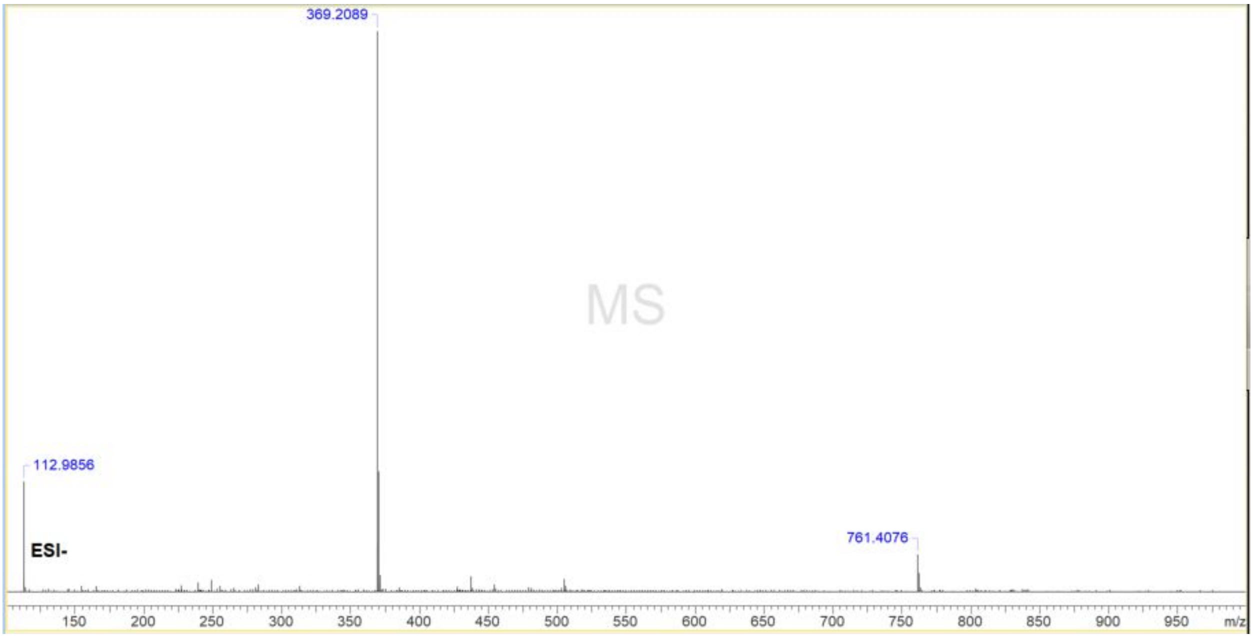

Figure S99: Tuaimenal A (**10**) HRESIMS (neg).

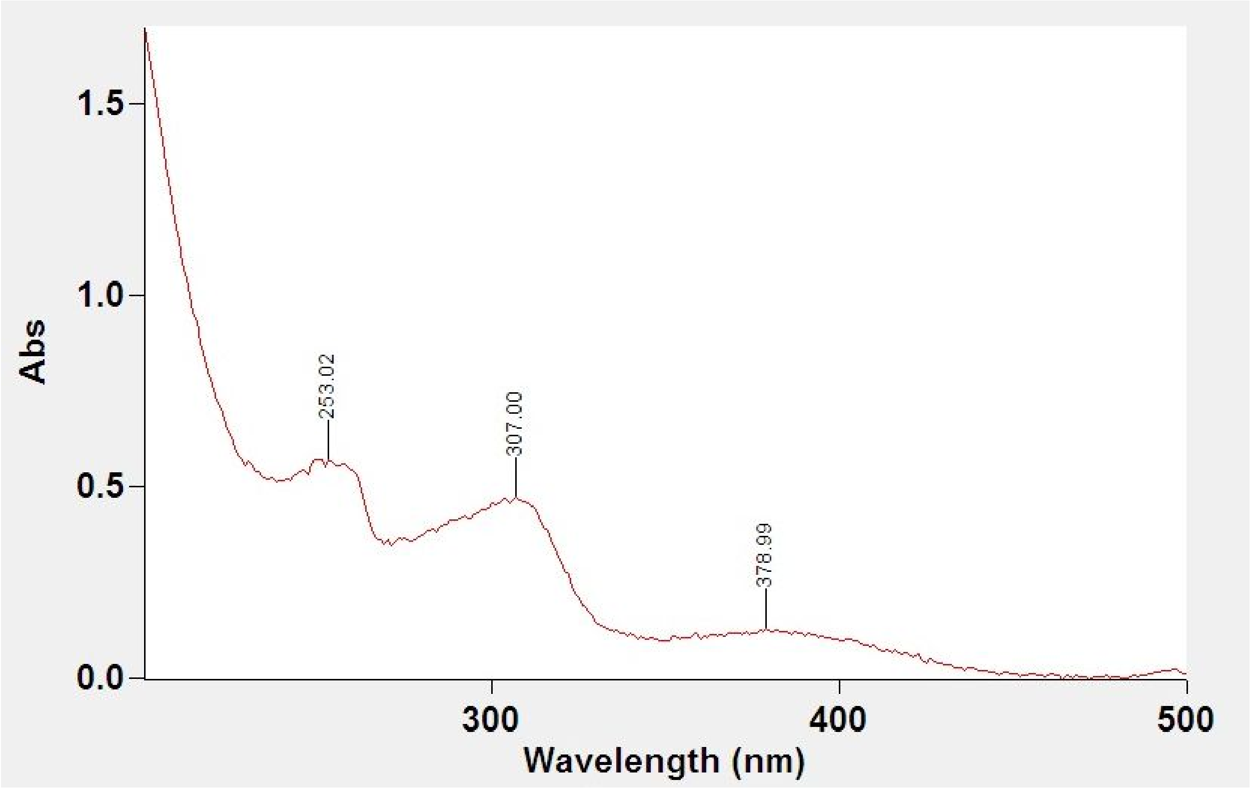

Figure S100: Tuaimenal A (**10**) UV λ_{max} (C_2H_3N).

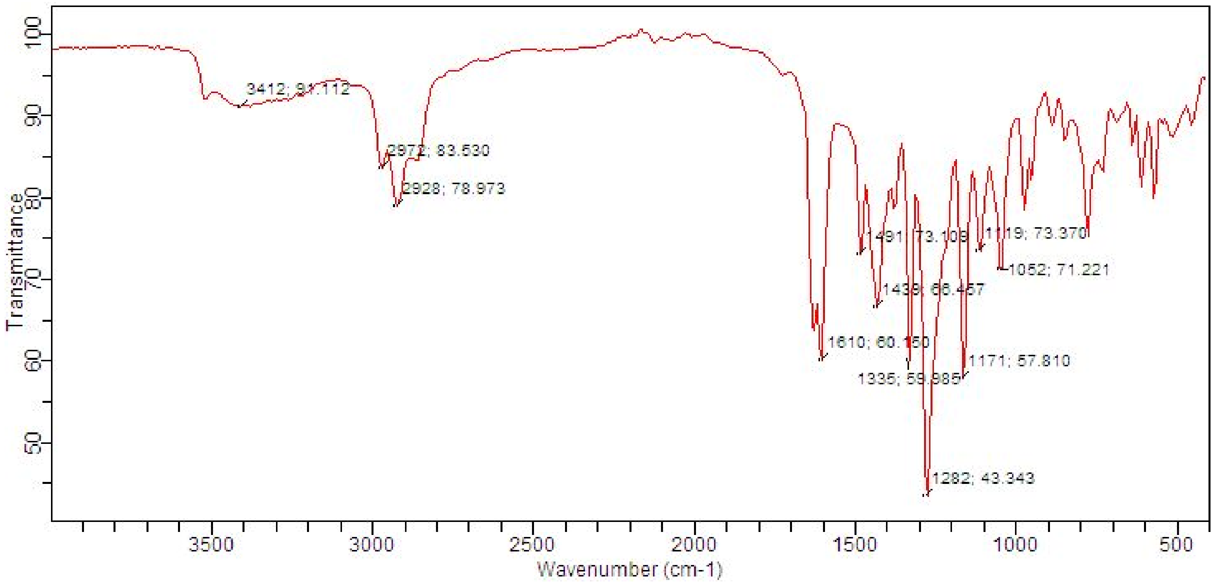

Figure S101: Tuaimenal A (**10**) IR spectrum (thin film).

Table S1: Comparison of tuaimenal A (**10**) ^{1}H and ^{13}C NMR spectra to literature.

**Tuaimenal A**[a]				
Pos	Literature δ_C[b], type	Literature δ_H[c]	Experimental δ_C[b], type	Experimental δ_H[c]
1	132.3, C		132.3, C	
2	151.4, C		151.4, C	
3	111.7, C		111.7, C	
4	158.2, C		158.2, C	
5	107.5, C		107.6, C	
6	118.7, C		118.7, C	
7	116.4, CH	6.86, d (10.1)	116.4, CH	6.86, d (10.2)
8	132.4, CH	5.80, d (10.1)	132.4, CH	5.80, d (9.1)
9	79.1, C		79.1, C	
10	40.2, CH_2	1.78, m	40.2, CH_2	1.78, m
11	22.6, CH_2	2.13, m	22.6, CH_2	2.12, m
12	123.3, CH	5.11, t	123.3, CH	5.11, t
13	135.8, C		135.8, C	
14	39.6, CH_2	1.99, t	39.6, CH_2	1.97, t
15	26.6, CH_2	2.05, m	26.6, CH_2	2.05, m
16	124.1, CH	5.07, t	124.2, CH	5.08, t
17	131.4, CH		131.4, CH	
18	17.7, CH_3	1.59, s	17.6, CH_3	1.60, s
19	25.7, CH_3	1.68, s	25.7, CH_3	1.68, s
20	16.0, CH_3	1.58, s	16.0, CH_3	1.58, s
21	25.4, CH_3	1.43, s	25.4, CH_3	1.43, s
22	7.6, CH_3	2.14, s	7.5, CH_3	2.14, s
23	191.1, CH	10.08, s	191.0, CH	10.08, s
OH_a		12.31, s		12.31, s
OH_b		6.37, s		6.32, s

[a] $CDCl_3$, ppm; [b] 150 MHz; [c] 600 MHz, multiplicity, (J (Hz))

Table S2: Comparison of *Duva florida* steroids (**8** and **9**) ^{13}C NMR spectra to literature.

Pos	Cmpd **8** Literature $\delta_C{}^b$, type	Cmpd **8** Experimental $\delta_C{}^c$, type	Cmpd **9** Experimental $\delta_C{}^c$, type
1	127.0	127.3	126.4
2	113.5	113.8	112.6
3	156.7	156.0	153.3
4	115.9	116.2	115.2
5	139.7	138.9	138.3
6	30.8	30.9	29.7
7	28.9	29.1	27.6
8	40.4	40.6	38.8
9	45.2	45.2	43.7
10	133.5	132.9	133.0
11	27.8	28.2	26.7
12	41.3	41.5	39.9
13	44.6	44.2	42.8
14	57.0	56.9	55.4
15	29.2	29.4	23.9
16	24.8	25.1	28.2
17	57.4	57.7	56.0
18	12.4	12.7	12.0
20	37.2	36.9	35.4
21	18.9	18.9	18.3
22	34.0	32.6	31.1
23	36.3	32.4	31.0
24	167.5	178.6	175.0
25			51.5

[a] $CDCl_3$, ppm; [b]125 MHz; [c]150 MHz

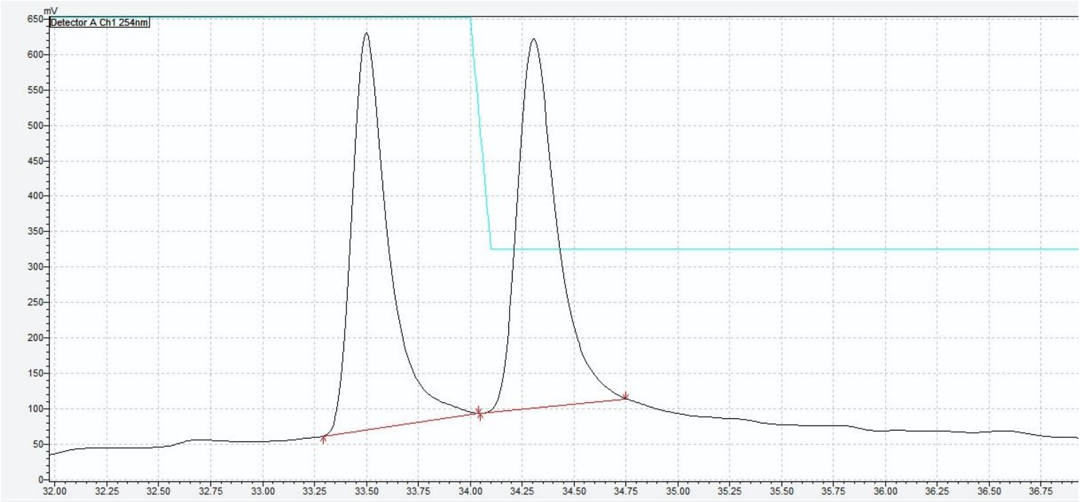

Results View - Peak Table

Peak Table | Compound | Group | Calibration Curve

Peak#	Ret. Time	Area	Height	Area%
1	33.499	6875194	560668	49.846
2	34.305	6917673	520516	50.154
Total		13792866	1081184	100.000

Figure S102: Integrated chromatogram displaying relative abundance of *R* and *S* enantiomers of tuaimenal E (**4**) separated utilizing a chiral HPLC column.
